# Inhibition of Microtubule Dynamics in Cancer Cells by Indole-Modified Latonduine Derivatives and Their Metal Complexes

**DOI:** 10.1021/acs.inorgchem.1c03154

**Published:** 2022-01-07

**Authors:** Christopher Wittmann, Anastasiia S. Sivchenko, Felix Bacher, Kelvin K. H. Tong, Navjot Guru, Thomas Wilson, Junior Gonzales, Hartmut Rauch, Susanne Kossatz, Thomas Reiner, Maria V. Babak, Vladimir B. Arion

**Affiliations:** † University of Vienna, Institute of Inorganic Chemistry, Währinger Strasse 42, A-1090 Vienna, Austria; ‡ Drug Discovery Lab, Department of Chemistry, City University of Hong Kong, 83 Tat Chee Avenue, Hong Kong SAR 999077, PR China; § Department of Radiology, Memorial Sloan Kettering Cancer Center, 417 East 68th Street, New York, New York 10065, United States; ∥ Department of Nuclear Medicine, University Hospital Klinikum Rechts der Isar, Technical University Munich, 81675 Munich, Germany; ⊥ TranslaTUM - Central Institute for Translational Cancer Research, D-81675 Munich, Germany; # Department of Chemistry, Technical University of Munich, D-85748 Munich, Germany; ∇ Department of Radiology, Weill Cornell Medical College, New York, New York 10021, United States; ○ Chemical Biology Program, Memorial Sloan Kettering Cancer Center, New York, New York 10065, United States

## Abstract

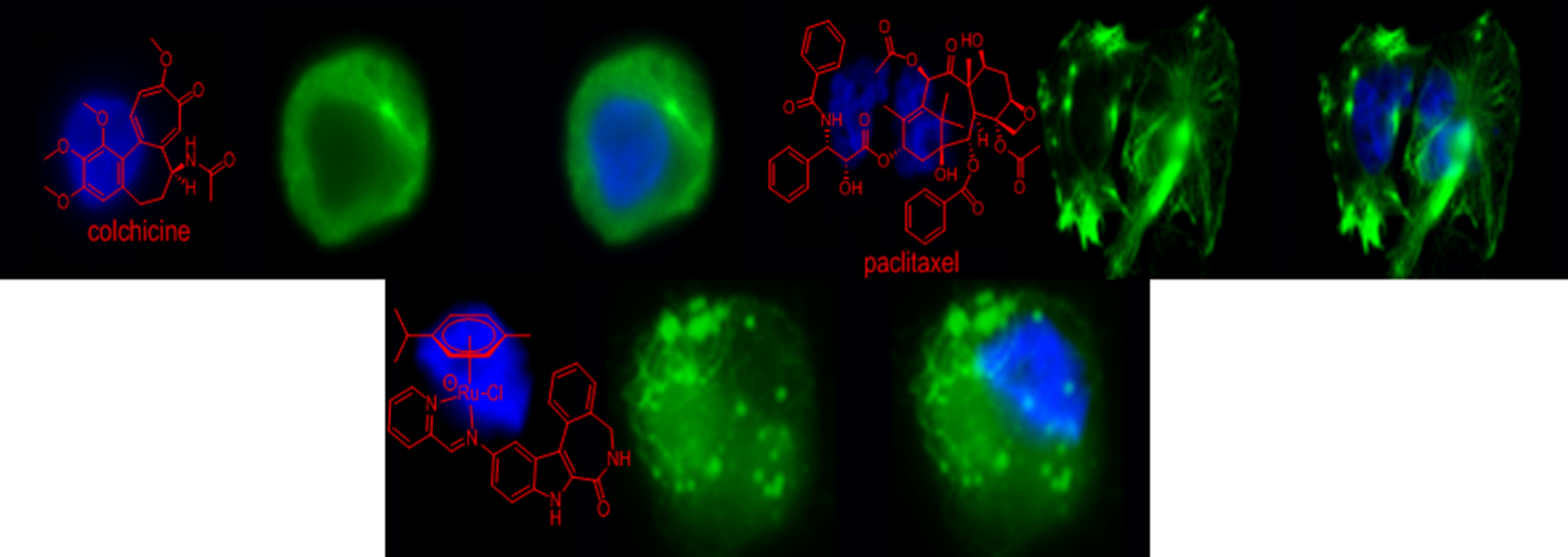

Indolo[2,3-*d*]benzazepines (indololatonduines) are rarely discussed in the literature. In this project, we prepared a series of novel indololatonduine derivatives and their Ru^II^ and Os^II^ complexes and investigated their microtubule-targeting properties in comparison with paclitaxel and colchicine. Compounds were fully characterized by spectroscopic techniques (^1^H NMR and UV–vis), ESI mass-spectrometry, and X-ray crystallography, and their purity was confirmed by elemental analysis. The stabilities of the compounds in DMSO and water were confirmed by ^1^H and ^13^C NMR and UV–vis spectroscopy. Novel indololatonduines demonstrated anticancer activity *in vitro* in a low micromolar concentration range, while their coordination to metal centers resulted in a decrease of cytotoxicity. The preliminary *in vivo* activity of the Ru^II^ complex was investigated. Fluorescence staining and *in vitro* tubulin polymerization assays revealed the prepared compounds to have excellent microtubule-destabilizing activities, even more potent than the well-known microtubule-destabilizing agent colchicine.

## Abstract 2

Several synthesized indololatonduine derivatives and their Ru^II^ and Os^II^ complexes were investigated for their microtubule-targeting properties in comparison with paclitaxel and colchicine. Fluorescence staining and *in vitro* tubulin polymerization assays indicate excellent microtubule-destabilizing activity. The compounds were even more potent than the well-known microtubule-destabilizing agent colchicine.

## Introduction

Microtubules, microfilaments, and intermediate filaments represent three major types of building blocks that make up the cell cytoskeleton.^1^ These proteins differ in their structural organization and function. In contrast to intermediate filaments, whose function is strictly structural,^2^ microfilaments and microtubules are highly dynamic structures. Microfilaments are flexible helical structures which are responsible for the cell movement and are predominantly composed of the most abundant protein actin.^3^ Microtubules are rigid hollow structures composed of α- and β-subunits of tubulin, responsible for maintaining cell shape, motility, and division. At any point of time, a subset of microtubules is rapidly growing by the addition of tubulin to their plus ends, while another subset is shrinking by depolymerization or pausing, thereby allowing rapid reorganization of cell cytoskeleton.^4,5^

**Scheme 1:**
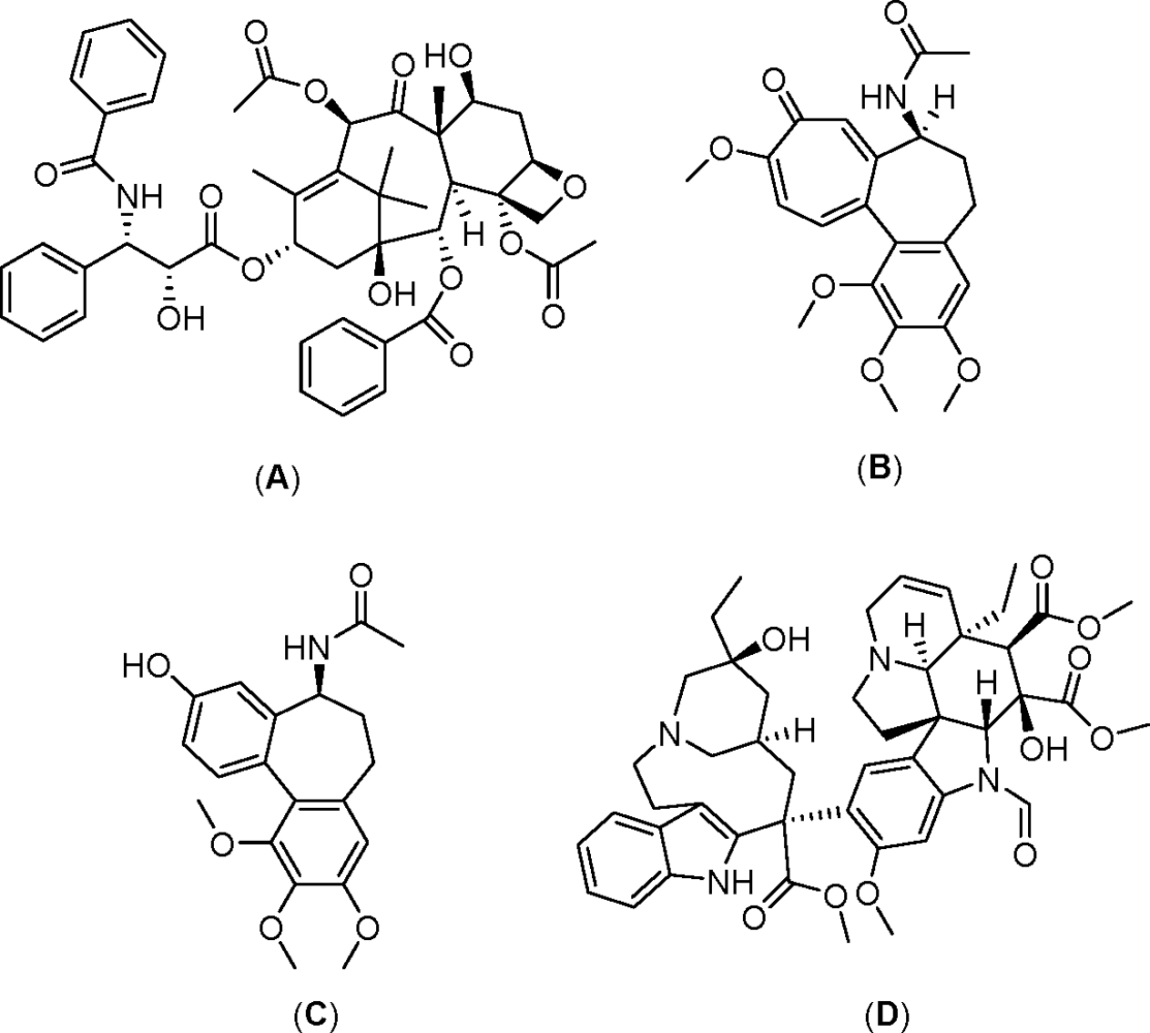
Chemical Structures of Microtubule-Stabilizing Drug Paclitaxel (**A**) and Microtubule-Destabilizing Compounds Colchicine (**B**), *N*-Acetylcolchinol (**C**), and Vincristine (**D**)

In particular, the process of skeleton reorganization is important in proliferating cells. When cells enter mitosis, microtubules do not arrange in the mitotic spindle. Hence, if dynamic instability is halted, the mitotic spindle is disrupted, leading to cell cycle arrest and cell death. The interference with microtubule dynamics has been investigated in the context of anticancer drug development, resulting in the emergence of several compound classes, including microtubule-stabilizing agents and microtubule-destabilizing agents. The microtubule-stabilizing agents, such as taxanes (paclitaxel, **A** in Scheme 1 and docetaxel), were shown to bind to polymerized tubulin microtubules, thereby shifting the equilibrium toward tubulin polymerization and protecting microtubules from disassembly.^6−8^ As a result of the increased microtubule polymer mass and disrupted microtubule dynamics, cancer cells were forced into mitotic arrest. In contrast, microtubule-destabilizing agents, such as colchicine (**B**), *N*-acetylcolchinol (**C**), and vinca alkaloids (vinblastine, vinorelbine, vincristine (**D**), and vindesine), enhanced tubulin depolymerization, which also resulted in the mitotic arrest.^7,8^ We note that colchicine and its analogues were shown to preferentially bind to the soluble monomeric tubulin, which halted microtubule dynamics upon addition to the microtubule ends, while binding of vinca alkaloids occurred directly at the exposed microtubule ends.^7^

**Scheme 2:**
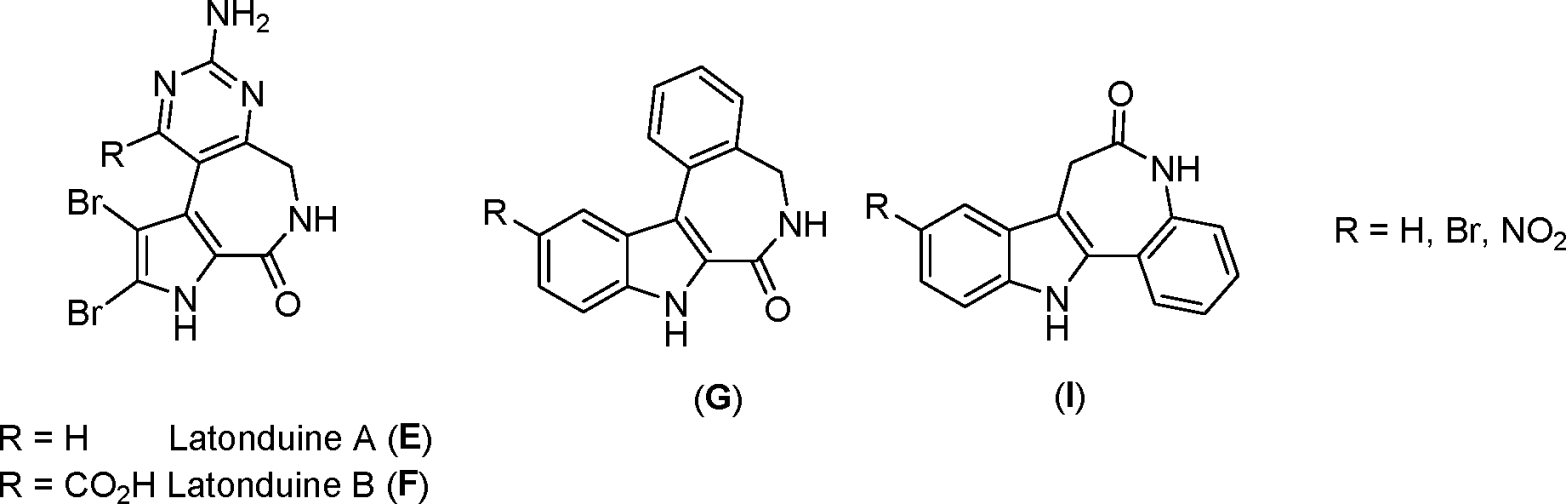
Chemical Structures of the Natural Products Latonduine A (**E**) and B (**F**) in Comparison with the Core Structures of Indolo[2,3-*d*]benzazepines (**G**) and Indolo[3,2-*d*]benzazepines (**I**, Paullones)

The systematic analysis of tubulin-targeting compounds revealed that many structures were characterized by a common structural element: an indole fragment.^9−11^ The indole core was present in tubulin-targeting compounds, obtained both from natural sources and synthetic small molecules.^9−11^ For example, marine natural products latonduine A and B (Scheme 2) did not show pronounced cytotoxic effects;^12,13^ however, their synthetic derivatives indolo[2,3-*d*]benzazepines (**G**, indololatonduines) demonstrated excellent antiproliferative activity in a nanomolar or submicromolar range,^14−17^ as well as improved *in vivo* effects on glioma grafted on the chick chorio-allantoic membrane in comparison with colchicine (**B**) and *N*-acetylcolchinol (**C**) (Scheme 1).^15^ The anticancer activity was related to their microtubule-destabilizing properties, achieved via tubulin binding at the colchicine-binding site.^17^ Interestingly, the anticancer activity of structurally similar indolo[3,2-*d*]benzazepines (**I**, paullones, Scheme 2) was linked to a different mechanism of action, namely inhibition of cyclin-dependent kinases (CDKs).^18,19^

**Scheme 3:**
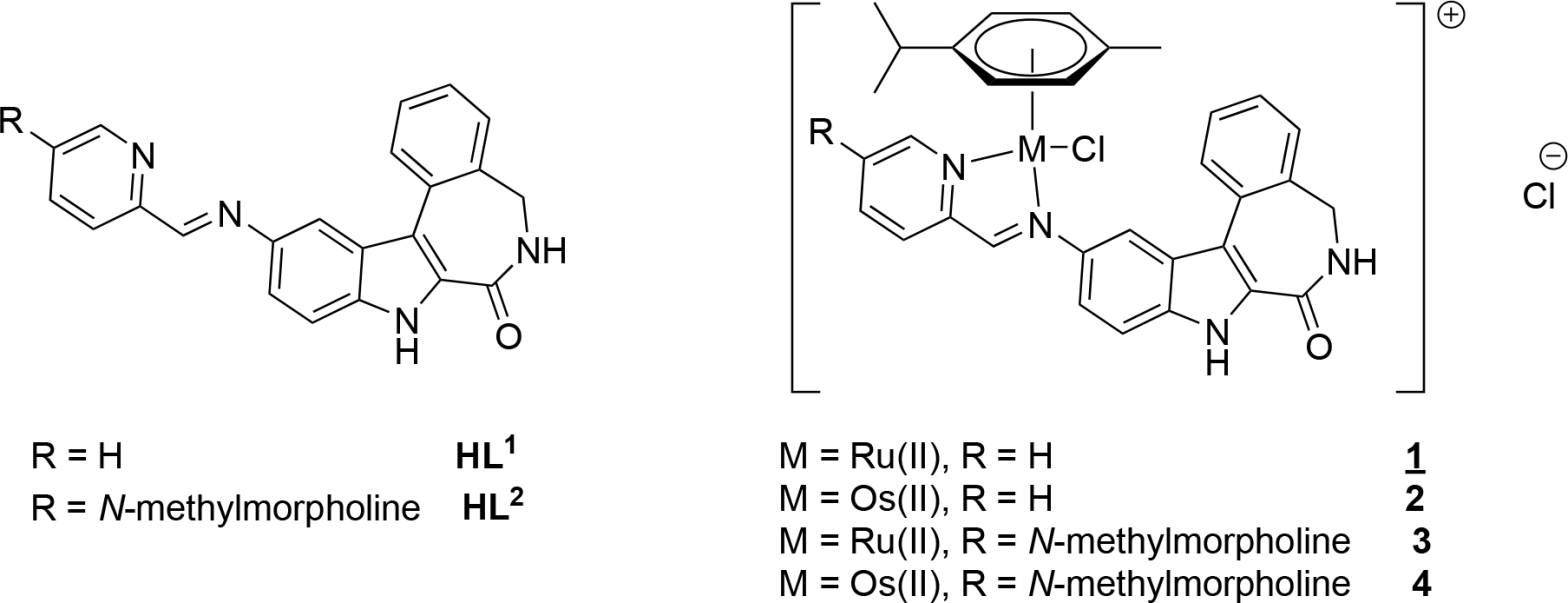
Chemical Structures of Indololatonduines **HL**^**1**^ and **HL**^**2**^ and the Corresponding Ru^II^ (**1** and **3**) and Os^II^ Complexes (**2** and **4**) Investigated in This Study The underlined number indicates compound studied by single crystal X-ray diffraction (SC-XRD).

Both microtubule-stabilizing and microtubule-destabilizing anticancer drugs are successfully used in clinics and experimental clinical trials. However, they are associated with several drawbacks, including severe neurotoxicity and drug resistance, in particular multidrug resistance (MDR) mediated by the Pgp efflux pump, as well as resistance associated with p53 mutations.^20−22^ Therefore, to battle the acquired resistance of cancer cells, novel tubulin-targeting agents are extensively developed. In this project, we aimed to prepare a novel series of indololatonduines and their metal complexes and investigate their tubulin-targeting properties (see Scheme 3). We have chosen a half-sandwich Ru(II) and Os(II) scaffold, since both organoruthenium^23,24^ and organoosmium^25^ anticancer complexes were shown to overcome MDR and exhibit a p53-independent mechanism of action.

The indole-fused latonduine backbone was modified to form a suitable bidentate ligand with and without a morpholine moiety, which typically translates into a more favorable pharmacological profile.^26−29^ Subsequently, the indololatonduines were coordinated to half-sandwich Ru^II^- and Os^II^-*p*-cymene moieties. The metal fragments were chosen based on the previous literature reports about related paullones with improved biological characteristics,^30−32^ additionally to the ability of various Ru complexes to target tubulin polymerization.^33,34^ We discuss the synthesis and structural features of novel compounds, their anticancer activity in cancer cell lines, as well as preliminary *in vivo* data. Importantly, we demonstrate that similar to the reported core latonduine structure^17^ the mechanism of action of novel compounds was linked to microtubule-destabilizing properties.

## Experimental Section

### Chemicals and Materials

2-Iodobenzonitrile and ethyl 5-nitro-1*H*-indole-2-carboxylate were purchased form ABCR (Germany). Borane (BH_3_) (1 M in THF), absolute *N*,*N*-dimethylformamide, *N*,*N*-dimethyl-4-aminopyridine (DMAP), di-*tert*-butyl-dicarbonate (Boc_2_O), absolute acetonitrile, palladium(II) acetate, sodium bicarbonate, aluminuim oxide, and 2-picolinaldehyde were received from Acros Organics. Ethoxy-methylchloride (EOMCl) was obtained from TCI. Sodium hydride in mineral oil, palladium on carbon (Pd/C), and Celite were bought from Sigma-Aldrich, while lithium hydroxide monohydrate and triphenylphosphine (PPh_3_) were bought from Alfa Aesar. Magnesium sulfate was bought from Fisher Chemicals. 1-Ethyl-3-(3-dimethylaminopropyl)carbodiimide hydrochloride (EDCI·HCl) was purchased from IRIS biotech. Silver(I) carbonate was purchased from Merck. The synthesis of 2-iodobenzylamine was carried out according to the literature procedure.^16^ [RuCl_2_(*p*-cymene)_2_]_2_^35^ as well as [OsCl_2_(*p*-cymene)_2_]_2_^36^ were also synthesized according to published protocols. The following reagents were used for biological experiments: glycerol (TCI chemicals), methanol (ACS, Anaqua), 1,4-dithio-dl-threitol (Alfa Aesar), glycine (Alfa Aesar), *N*,*N*,*N*′,*N*′-tetramethylethylenediamine (TEMED) (Alfa Aesar), TRIS (hydroxymethyl)aminomethane (Alfa Aesar), Triton X-100 (Alfa Aesar), sodium deoxycholate (TCI chemicals), tris(hydroxymethyl)aminomethane hydrochloride (Alfa Aesar), ammonium peroxodisulfate (TCI chemicals), ammonium persulfate (Alfa Aesar), bromophenol blue disodium salt (TCI chemicals), Tween 20 (TCI chemicals), SDS (MACKLIN), acrylamide/Bis Solution (Bio-Rad), sodium azide (Sigma-Aldrich), IGEPAL CA-630 Sigma, Ponceau S (Alfa Aesar), albumin, bovine (MP biomedicals), and Pierce Protease and Phosphatase Inhibitor Mini Tablets (Thermo Scientific). Anti-tubulin (DM1A) mouse monoclonal antibodies no. 3873 and anti-mouse IgG HRP-linked antibody were purchased from Cell Signaling Technology (no. 7076) and were used for Western Blot. Nitrocellulose membrane (roll, 0.45 μm, 30 cm × 3.5 m, no. 1620115) was used. Immunodetection was visualized by chemiluminescence, using Immobilon Crescendo Western HPR substrate (WBLUR0100), and analyzed using chemiluminescence imaging machine (ChemiDoc Touch Imaging System, BioRad). Cells were grown in tissue culture 75 cm^2^ (T-75) flasks and 6-well culture plates (polystyrene coating, Greiner Bio-One). Fluorescence microscopy used anti-tubulin (DM1A) mouse monoclonal antibodies (no. 3873), mounting solution (ProLong Gold Antifade Mountant, 1 drop), 4′,6-diamidino-2-phenylindole (DAPI, Invitrogene), and Thermo Fisher Alexa Fluor 488-conjugated secondary antibodies.

### Synthesis of Indololatonduines and Their Metal Complexes

#### Ethyl 5-nitro-1-(ethoxymethyl)-1*H*-indole-2-carboxylate (**J**) (see Scheme 4)

Ethyl 5-nitro-1*H*-indole-2-carboxylate (6.0 g, 25.6 mmol) was dissolved in absolute DMF (60 mL) under argon atmosphere. The solution was cooled to 0 °C, and NaH (60% in mineral oil; 1.54 g, 38.4 mmol) was added carefully, as the reaction is highly exothermic. The reaction mixture was stirred at room temperature for 1 h before it was cooled again to 0 °C. Then EOMCl (4.8 mL, 51.2 mmol) was added dropwise, and the content was stirred at room temperature overnight. The solvent was removed at 10 mbar and 60 °C, and the residue was taken up in water (40 mL). The aqueous suspension was extracted with DCM (4 × 40 mL). The combined organic phases were dried over MgSO_4_ and concentrated *in vacuo*. The crude product was purified on silica using hexane/ethyl acetate 9:1 as eluent, affording **J** as a white powder. Yield: 7.36 g, 98%. ^1^H NMR (500 MHz, DMSO-*d*_6_) δ 8.77 (d, *J* = 2.1 Hz, 1H), 8.22 (dd, *J* = 9.2, 2.2 Hz, 1H), 7.93 (d, *J* = 9.3 Hz, 1H), 7.62 (s, 1H), 6.03 (s, 2H), 4.37 (q, *J* = 7.1 Hz, 2H), 3.42 (q, *J* = 7.0 Hz, 2H), 1.35 (t, *J* = 7.1 Hz, 3H), 1.02 (t, *J* = 7.0 Hz, 3H). ESI-MS (acetonitrile/methanol +1% water), positive: *m*/*z* 315.05 [M + Na]^+^, 607.09 [2M + Na]^+^.

**Scheme 4:**
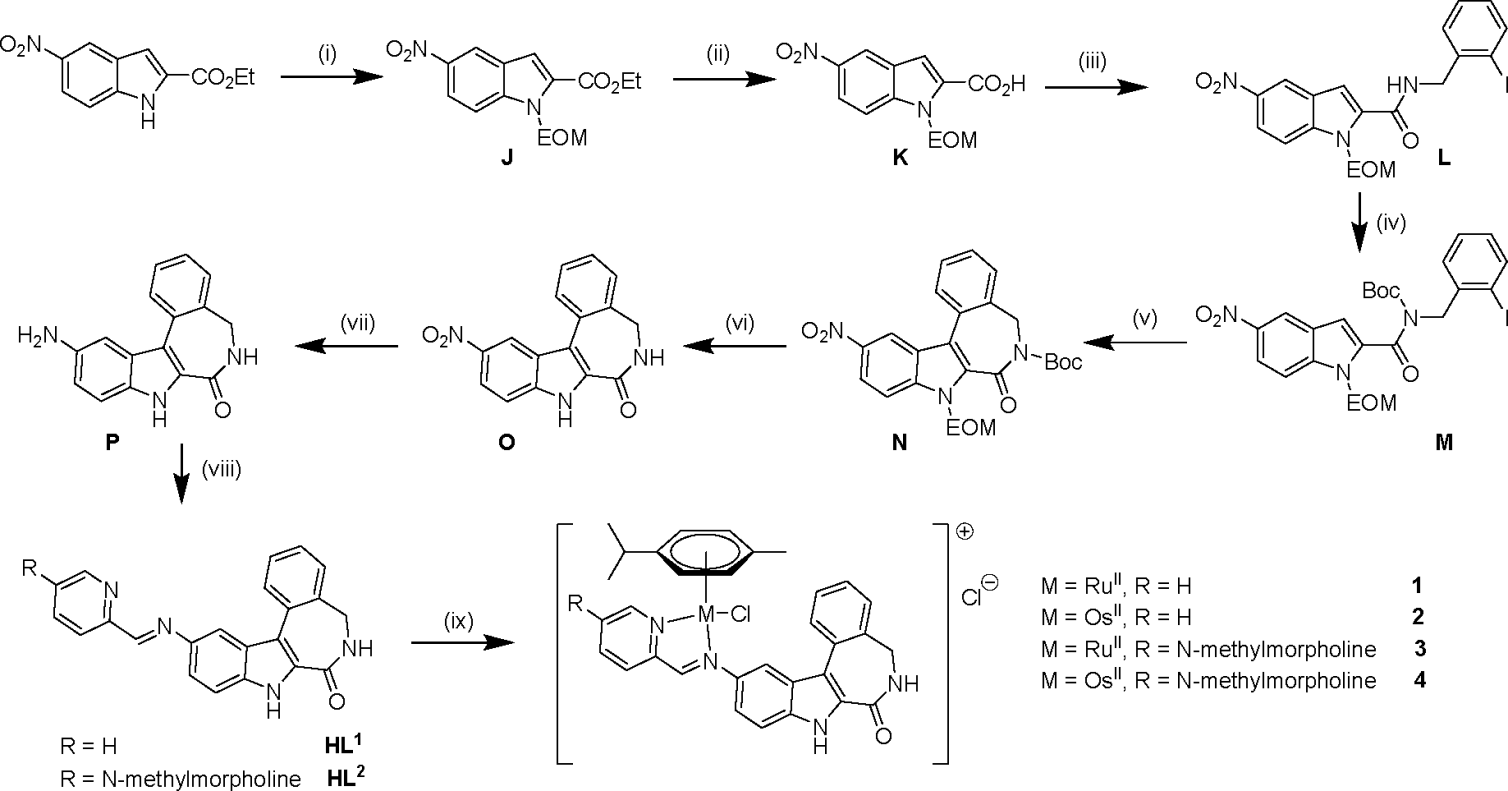
Synthesis Pathway for the Novel Latonduine Derivatives Reagents and conditions: (i) NaH, EOMCl, DMF_dry_, 0 °C to r.t., 16 h; (ii) LiOH, 1 M HCl, EtOH, 100 °C, 2 h; (iii) 2-iodobenzylamine, EDCI, DMAP, dry CHCl_3_, 0 °C to r.t., 16 h; (iv) Boc_2_O, DMAP, dry MeCN, 0 °C to r.t., 16 h; (v) Pd(OAc)_2_, PPh_3_, Ag_2_CO_3_, dry DMF, 100 °C, 2 h; (vi) HCl, EtOH, 100°C, 4 h; (vii) Pd/C, H_2_, dry THF, 20 h; (viii) 2-formylpyridine or 5-(*N*-methylmorpholine)-2-formylpyridine, dry EtOH, 85 °C, 16 h; (ix) ^i^PrOH, CHCl_3_, [M^II^Cl(μ-Cl)(η^6^-*p*-cymene)]_2_ (M = Ru and Os), 60 °C, 1 h, light protection.

#### 5-Nitro-1-(ethoxymethyl)-1*H*-indole-2-carboxylic Acid (**K**)

To a solution of species **J** (4.55 g, 15.6 mmol) in ethanol (200 mL) at 50 °C was added a solution of LiOH·H_2_O (0.79 g, 18.7 mmol) in water (7 mL). The reaction mixture was heated to reflux for 2 h, and then the solvent was removed under reduced pressure. The residue was taken up in water (50 mL) and acidified with 1 M HCl (35 mL). The white voluminous precipitate was directly extracted with ethyl acetate (3 × 80 mL). The combined organic phases were dried over MgSO_4_ and concentrated *in vacuo*. The isolated crude product was recrystallized from ethanol (95 mL) to give light yellow needles. Yield: 3.78 g, 92%. ^1^H NMR (500 MHz, DMSO-*d*_6_) δ 13.52 (s, 1H), 8.75 (d, *J* = 2.3 Hz, 1H), 8.21 (dd, *J* = 9.2, 2.3 Hz, 1H), 7.90 (d, *J* = 9.3 Hz, 1H), 7.56 (s, 1H), 6.06 (s, 2H), 3.44–3.39 (m, 2H), 1.02 (t, *J* = 7.0 Hz, 3H). ESI-MS (acetonitrile/methanol + 1% water), negative: *m*/*z* 263.05 [M – H]^−^, 549.11 [2M – 2H + Na]^−^.

#### 5-Nitro-1-(ethoxymethyl)-N-(2-iodobenzyl)-1*H*-indole-2-carboxamide (**L**)

Species **K** (4.74 g, 17.9 mmol), 2-iodobenzylamine (3.80 g, 16.3 mmol), EDCI·HCl (3.43 g, 17.9 mmol), and DMAP (2.2 g, 17.9 mmol) were dissolved in dry chloroform (170 mL) at 0 °C under an argon atmosphere. The solution was stirred at 0 °C for 4 h and then at room temperature overnight. The precipitate was filtered off as a white solid, washed with diethyl ether, and air-dried. Yield: 7.15 g, 92%. ^1^H NMR (500 MHz, DMSO-*d*_6_) δ 9.41 (t, *J* = 5.8 Hz, 1H), 8.76 (d, *J* = 2.3 Hz, 1H), 8.18 (dd, *J* = 9.2, 2.3 Hz, 1H), 7.92–7.84 (m, 2H), 7.49 (s, 1H), 7.39 (dd, *J* = 11.2, 4.9 Hz, 1H), 7.33 (d, *J* = 6.2 Hz, 1H), 7.06 (dd, *J* = 11.2, 3.9 Hz, 1H), 6.03 (s, 2H), 4.44 (d, *J* = 5.7 Hz, 2H), 3.40–3.37 (m, 2H), 1.01 (t, *J* = 7.0 Hz, 3H). ESI-MS (acetonitrile/methanol + 1% water), negative: *m*/*z* 478.03 [M – H]^−^.

#### *tert*-Butyl-(5-nitro-1-(ethoxymethyl)-1*H*-indole-2-carbonyl)(2-iodobenzyl)carbamate (**M**)

To a solution of **L** (7.15 g, 14.9 mmol) in dry acetonitrile (140 mL) Boc_2_O (5.86 g, 26.8 mmol) and DMAP (1.82 g, 14.9 mmol) were added under argon atmosphere. The reaction mixture was stirred at room temperature overnight. The solvent was evaporated and the residue was extracted with a mixture of ethyl acetate (120 mL) and water (80 mL). The aqueous phase was separated and extracted with ethyl acetate (2 × 120 mL). The combined organic phases were dried over MgSO_4_ and concentrated *in vacuo*. The isolated crude product was purified on silica using hexane/ethyl acetate 78:22 as eluent resulting in a yellow powder of Boc-protected species **M**. Yield: 7.5 g, 87%. ^1^H NMR (500 MHz, DMSO-*d*_6_) δ 8.75 (d, *J* = 2.3 Hz, 1H), 8.20 (dd, *J* = 9.2, 2.3 Hz, 1H), 7.93 (dd, *J* = 11.9, 5.2 Hz, 2H), 7.42 (dd, *J* = 11.8, 4.4 Hz, 1H), 7.32 (s, 1H), 7.21 (d, *J* = 7.8 Hz, 1H), 7.08 (t, *J* = 6.8 Hz, 1H), 5.81 (s, 2H), 4.90 (s, 2H), 3.46 (q, *J* = 7.0 Hz, 2H), 1.11–1.06 (m, 12H). ESI-MS (acetonitrile/methanol + 1% water), positive: *m*/*z* 925.33 [2 M + Na]^+^, 941.24 [2 M + K]^+^.

#### *tert*-Butyl-11-nitro-8-(ethoxymethyl)-dihydroindolo[2,3-*d*]benzazepin-7-one (**N**)

To a solution of **M** (4.92 g, 8.4 mmol) in dry DMF (80 mL) under argon atmosphere were added palladium(II) acetate (0.47 g, 2.1 mmol), triphenylphosphine (1.10 g, 4.2 mmol), and silver(I) carbonate (5.80 g, 21.0 mmol), and the mixture was stirred at 100 °C for 2 h. The solvent was removed at 10 mbar and 60 °C, and the black residue was taken up in DCM (160 mL). The suspension was filtered over Celite and rinsed with DCM (2 × 30 mL). The solvent was removed, and the crude product was purified on silica using hexane/ethyl acetate 78:22 as eluent. After slow evaporation of the solvent, yellow crystals of X-ray diffraction quality were obtained. Yield: 5.3 g, 93%. ^1^H NMR (500 MHz, DMSO-*d*_6_) δ 8.86 (d, *J* = 2.2 Hz, 1H), 8.34 (dd, *J* = 9.2, 2.2 Hz, 1H), 8.11 (d, *J* = 7.5 Hz, 1H), 8.06 (d, *J* = 9.3 Hz, 1H), 7.69 (td, *J* = 7.6, 1.3 Hz, 1H), 7.61 (d, *J* = 6.6 Hz, 1H), 7.54 (td, *J* = 7.5, 0.9 Hz, 1H), 6.03 (dd, *J* = 29.5, 10.8 Hz, 2H), 5.12 (d, *J* = 15.2 Hz, 1H), 4.31 (d, *J* = 15.1 Hz, 1H), 3.53 (dq, *J* = 9.4, 7.0 Hz, 1H), 3.45–3.38 (m, 1H), 1.48 (s, 9H), 1.05 (t, *J* = 7.0 Hz, 3H). ESI-MS (acetonitrile/methanol + 1% water), positive: *m*/*z* 925.34 [2M + Na]^+^.

#### 11-Nitro-5,8-dihydroindolo[2,3-*d*]benzazepin-7(6H)-one (**O**)

A solution of **N** (1.00 g, 2.3 mmol) in ethanol/HCl_conc_ 4:1 (100 mL) was stirred at 100 °C for 4 h. After cooling down to room temperature, the solution was neutralized with saturated aqueous solution of NaHCO_3_ (∼150 mL). The precipitate that formed was filtered off and washed with water. The crude product was purified on silica by using DCM/MeOH 98:2 as eluent and isolated as a yellow powder. Yield: 0.40 g, 60%. ^1^H NMR (500 MHz, DMSO-*d*_6_) δ 12.50 (s, 1H), 10.81 (t, *J* = 5.6 Hz, 1H), 8.85 (d, *J* = 2.1 Hz, 1H), 8.24 (dd, *J* = 9.1, 2.2 Hz, 1H), 8.01 (d, *J* = 7.5 Hz, 1H), 7.78 (d, *J* = 9.1 Hz, 1H), 7.62 (td, *J* = 7.5, 1.5 Hz, 1H), 7.57–7.44 (m, 2H), 4.42–4.34 (m, 1H), 4.13 (d, *J* = 14.2 Hz, 1H). ESI-MS (acetonitrile/methanol + 1% water), negative: *m*/*z* 292.00.[M – H]^−^.

#### 11-Amino-5,8-dihydroindolo[2,3-*d*]benzazepin-7(6*H*)-one (**P**)

Species **O** (0.10 g, 0.341 mmol) and 10% Pd/C (0.1 equiv) were suspended in dry THF (40 mL) under argon atmosphere. The solution was stirred under a H_2_ atmosphere at 3 bar at room temperature for 20 h. The solution was filtered over Celite, and the solvent was removed under reduced pressure. The crude product was purified on silica by using DCM/MeOH 95:5 as eluent. After slow evaporation of the solvent, colorless crystals of X-ray diffraction quality were isolated. Yield: 0.09 g, 99%. ^1^H NMR (500 MHz, DMSO-*d*_6_) δ 11.65 (s, 1H), 8.34 (t, *J* = 5.4 Hz, 1H), 7.95 (d, *J* = 7.5 Hz, 1H), 7.62–7.46 (m, 2H), 7.36 (dd, *J* = 7.9, 6.9 Hz, 1H), 7.32 (d, *J* = 8.7 Hz, 1H), 7.23 (d, *J* = 1.6 Hz, 1H), 6.81 (dd, *J* = 8.7, 2.0 Hz, 1H), 4.86 (s, 2H), 4.10 (d, *J* = 4.9 Hz, 2H). ESI-MS (acetonitrile/methanol + 1% water), positive: *m*/*z* 264.11 [M + H]^+^.

#### **HL**^**1**^·H_2_O

Amine **P** (0.22 g, 0.84 mmol) was suspended in anoxic ethanol (10 mL) in a 25 mL Schlenk tube. Then 2-formylpyridine (120 μL, 1.26 mmol) was added, and the mixture was stirred at 85 °C overnight. The next day, after cooling to room temperature, the slightly brown precipitate was filtered off and washed with ethanol. Yield: 0.29 g, 98%. Anal. Calcd for C_22_H_16_N_4_O·H_2_O (*M*_r_ = 370.40), %: C, 71.34; H, 4.90; N, 15.13. Found, %: C, 70.83; H, 4.54; N, 14.89. ^1^H NMR (600 MHz, DMSO-*d*_6_) δ 12.17 (s, 1H, H^8^), 8.76 (s, 1H, H14), 8.74–8.69 (m, 1H, H^17^), 8.44 (t, *J* = 5.4 Hz, 1H, H^6^), 8.20 (d, *J* = 7.9 Hz, 1H, H^20^), 8.03 (d, *J* = 7.5 Hz, 1H, H^1^), 7.98–7.91 (m, 2H, H^19^, H^12^), 7.60 (d, *J* = 8.7 Hz, 1H, H^9^), 7.55–7.45 (m, 4H, H^2^, H^4^, H^18^, H^10^), 7.39–7.31 (m, 1H, H^3^), 4.10 (d, *J* = 5.3 Hz, 2H, H^5^). ^13^C NMR (151 MHz, DMSO-*d*_6_) δ 163.4 (Cq, C^7^), 158.4 (CH, C^14^), 154.5 (Cq, C^15^), 149.6 (CH, C^17^), 143.9 (Cq, C^11^), 137.0 (CH, C^19^), 136.9 (Cq, C^4a^), 135.9 (Cq, C^8a^), 133.1 (Cq, C^12c^), 130.9 (Cq, C^7a^), 128.3 (CH, C^4^), 128.2 (CH, C^2^), 127.4 (CH, C^1^), 126.5 (CH, C^3^), 125.2 (CH, C^18^), 124.9 (Cq, C^12a^), 120.9 (CH, C^20^), 118.9 (CH, C^10^), 116.7 (Cq, C^12b^), 113.6 (CH, C^12^), 113.4 (CH, C^9^), 44.6 (CH_2_, C^5^). For the atom numbering scheme, see Chart S1. ESI-MS (acetonitrile/methanol + 1% water), positive: *m*/*z* 353.26 [M + H]^+^.

#### **HL**^**2**^·0.5C_2_H_5_OH

Amine **P** (0.21 g, 0.80 mmol) and 5-methylmorpholinyl-2-formylpyridine (0.17 mg, 0.80 mmol) were suspended in anoxic ethanol (10 mL) in a 25 mL Schlenk tube and stirred at 85 °C overnight. The next day, after the solution was cooled to room temperature, the gray-white precipitate was filtered off and washed with ethanol. Yield: 0.29 g, 79%. Anal. Calcd for C_27_H_25_N_5_O_2_·0.5C_2_H_5_OH (*M*_r_ = 474.55), %: C, 70.86; H, 5.95; N, 14.75. Found, %: C, 70.49; H, 5.88; N, 14.44. ^1^H NMR (600 MHz, DMSO-*d*_6_) δ 12.18 (s, 1H, H^12^), 8.75 (s, 1H, H^14^), 8.62 (s, 1H, H^17^), 8.45 (s, 1H, H^5^), 8.17 (d, *J* = 8.0 Hz, 1H, H^20^), 8.02 (d, *J* = 7.5 Hz, 1H, H^1^), 7.94 (s, 1H, H^12^), 7.87 (d, *J* = 7.5 Hz, 1H, H^19^), 7.60 (d, *J* = 8.7 Hz, 1H, H^9^), 7.52 (dd, *J* = 12.8, 5.1 Hz, 1H, H^2^), 7.49 (d, 1H, H^10^), 7.47 (s, 1H, H^4^), 7.35 (t, *J* = 7.2 Hz, 1H, H^3^), 4.09 (s, 2H, H^5^), 3.58 (d, *J* = 2.4 Hz, 2H, H^21^), 3.57 (s, 4H, H^24a^, H^24b^), 2.38 (s, 4H, H^23a^, H^23b^). ^13^C NMR (151 MHz, DMSO-*d*_6_) δ 163.4 (Cq, C^7^), 158.2 (CH, C^14^), 153.6 (Cq, C^15^), 149.9 (CH, C^17^), 144.0 (Cq, C^11^), 137.1 (CH, C^19^), 136.9 (Cq, C^4a^), 135.9 (Cq, C^8a^), 135.1 (Cq, C^18^) 133.2 (Cq, C^12c^), 131.0 (Cq, C^7a^), 128.5 (CH, C^2^), 128.5 (CH, C^4^), 127.4 (CH, C^1^), 126.2 (CH, C^3^), 124.9 (Cq, C^12a^), 120.6 (CH, C^20^), 118.7 (CH, C^10^), 116.7 (Cq, C^12b^), 113.4 (CH, C^12^), 113.4 (CH, C^9^), 66.2 (2 × CH_2_, C^23^), 59.1 (CH_2_, C^21^), 53.0 (2 × CH_2_, C^24^), 44.5 (CH_2_, C^5^). ESI-MS (acetonitrile/methanol + 1% water), positive: *m*/*z* 452.37 [M + H]^+^.

#### **1**·C_3_H_8_O·0.8H_2_O

To a solution of **HL**^**1**^ (100 mg, 0.28 mmol) in isopropanol (40 mL) was added a solution of [RuCl_2_(*p*-cymene)_2_]_2_ (87 mg, 0.14 mmol) in chloroform (1.5 mL). The resulting mixture was stirred at 50 °C for 1 h. After cooling to room temperature, the solution was placed in the fridge at 4 °C overnight. The next day the ocher-colored precipitate was filtered off and washed with isopropanol. Yield: 180 mg, 98%. Anal. Calcd for C_32_H_30_Cl_2_N_4_ORu·C_3_H_8_O·0.8H_2_O (*M*_r_ = 733.08), %: C, 57.34; H, 5.44; N, 7.64. Found, %: C, 57.35; H: 5.13; N: 7,54. ^1^H NMR (600 MHz, DMSO-*d*_6_) δ 12.51 (s, 1H, H^8^), 9.60 (d, *J* = 5.5 Hz, 1H, H^17^), 9.04 (s, broad, 1H, H^14^), 8.56 (t, *J* = 5.5 Hz, 1H, H^6^), 8.42 (s, 1H, H^12^), 8.34–8.22 (m, 2H, H^19^, H^20^), 8.05 (s, broad, 1H, H^1^), 7.91–7.84 (m, 2H, H^10^, H^18^), 7.74 (d, *J* = 8.8 Hz, 1H, H^9^), 7.60–7.49 (m, 2H, H^4^, H^2^), 7.41 (td, *J* = 7.5, 0.9 Hz, 1H, H^3^), 6.09 (d, *J* = 6.1 Hz, 1H, H^cy2^), 5.77 (d, *J* = 6.1 Hz, 1H, H^cy1^), 5.71 (d, *J* = 6.0 Hz, 1H, H^cy2^), 5.56 (d, *J* = 6.0 Hz, 1H, H^cy1^), 4.14 (d, *J* = 5.1 Hz, 2H, H^5^), 2.59–2.45 (m, 1H, H^cy3^ (overlapped DMSO-signal)), 2.17 (s, 3H, H^cy5^), 1.00 (dd, *J* = 11.8, 6.9 Hz, 6H, H^cy4^). ^13^C NMR (151 MHz, DMSO-*d*_6_) δ 166.5 (CH, C^14^), 163.1 (Cq, C^7^), 155.9 (CH, C^17^), 154.9 (Cq, C^15^), 146.0 (Cq, C^11^), 139.8 (CH, C^19^), 137.2 (Cq, C^4a^), 136.7 (Cq, C^8a^), 132.8 (Cq, C^12c^), 132.0 (Cq, C^7a^), 129.6 (CH, C^20^), 128.5 (CH, C^18^), 128.4 (CH, C^4^), 128.2 (CH, C^2^), 127.4 (CH, C^1^), 126.9 (CH, C^3^), 124.0 (Cq, C^12a^), 120.5 (CH, C^10^), 117.1 (Cq, C^12b^), 114.1 (CH, C^12^), 113.3 (CH, C^9^), 105.1 (Cq, C^cy2a^), 103.1 (Cq, C^cy1a^), 86.6 (CH, C^cy2^), 85.7 (CH, C^cy2^), 85.3 (CH, C^cy1^), 85.2 (CH, C^cy1^), 44.6 (CH_2_, C^5^), 30.5 (CH, C^cy3^), 21.9 (CH_3_, C^cy4^), 21.4 (CH_3_, C^cy4^), 18.3 (CH_3_, C^cy5^). For the atom numbering scheme, see Chart S1. ESI-MS (acetonitrile/methanol + 1% water), positive: *m*/*z* 623.19 [M – Cl]^+^. Single crystals of X-ray diffraction quality were obtained by slow diffusion of diethyl ether into a DMF solution of the product.

#### **2**·C_3_H_8_O·H_2_O

To a solution of **HL**^**1**^ (100 mg, 0.28 mmol) in isopropanol (40 mL) was added a solution of [OsCl_2_(*p*-cymene)_2_]_2_ (112 mg, 0.14 mmol) in chloroform (1.5 mL). The resulting mixture was stirred at 50 °C for 1 h. After cooling to room temperature, the flask was placed in a fridge at 4 °C overnight. The next day the dark brown precipitate was filtered off and washed with isopropanol. Yield: 148 mg, 71%. Anal. Calcd for C_32_H_30_Cl_2_N_4_OOs·C_3_H_8_O·H_2_O (*M*_r_ = 825.85), %: C, 50.90; H, 4.88; N, 6.78. Found, %: C, 51.04; H, 4.63; N, 6.73. ^1^H NMR (600 MHz, DMSO-*d*_6_) δ 12.51 (s, 1H, H^8^), 9.54 (d, *J* = 5.6 Hz, 1H, H^17^), 9.44 (s, broad, 1H, H^14^), 8.56 (t, *J* = 5.4 Hz, 1H, H^6^), 8.44–8.32 (m, 2H, H^12^, H^20^), 8.27 (dd, *J* = 11.6, 3.9 Hz, 1H, H^19^), 8.03 (s, broad, 1H, H^1^), 7.83 (ddd, *J* = 7.4, 5.8, 1.4 Hz, 1H, H^18^), 7.78 (dd, *J* = 8.8, 2.0 Hz, 1H, H^10^), 7.72 (d, *J* = 8.8 Hz, 1H, H^9^), 7.58–7.50 (m, 2H, H^4^, H^2^), 7.43–7.37 (m, 1H, H^3^), 6.38 (d, *J* = 5.8 Hz, 1H, H^cy2^), 5.98 (d, *J* = 5.8 Hz, 1H, H^cy1^), 5.91 (d, *J* = 5.7 Hz, 1H, H^cy2^), 5.71 (d, *J* = 5.6 Hz, 1H, H^cy1^), 4.14 (d, *J* = 5.0 Hz, 2H, H^5^), 2.46–2.39 (m, 1H, H^cy3^), 2.26 (s, 3H, H^cy5^), 0.93 (dt, *J* = 27.7, 13.8 Hz, 6H, H^cy4^). ^13^C NMR (151 MHz, DMSO-*d*_6_) δ 167.4 (CH, C^14^), 163.1 (Cq, C^7^), 156.3 (Cq, C^15^), 155.6 (CH, C^17^), 146.0 (Cq, C^11^), 140.0 (CH, C^19^), 137.2 (Cq, C^4a^), 136.8 (Cq, C^8a^), 132.7 (Cq, C^12c^), 132.0 (Cq, C^7a^), 129.4 (CH, C^20^), 129.3 (CH, C^18^), 128.4 (CH, C^2^), 128.2 CH, C^4^), 127.3 (CH, C^1^), 126.9 (CH, C^3^), 123.9 (Cq, C^12a^), 120.9 (CH, C^10^), 117.0 (Cq, C^12b^), 114.5 (CH, C^12^), 113.4 (CH, C^9^), 97.0 (Cq, C^cy1a^), 96.6 (Cq, C^cy2a^), 78.8 (CH, C^cy2^), 77.7 (CH, C^cy2^), 75.9 (CH, C^cy1^), 75.6 (CH, C^cy1^), 44.6 (CH_2_, H^5^), 30.7 (CH, C^cy3^), 22.1 (CH_3_, C^cy4^), 21.8 (CH_3_, C^cy4^), 18.3 (CH_3_, C^cy5^). ESI-MS (acetonitrile/methanol + 1% water), positive: *m*/*z* 713.24 [M – Cl]^+^.

#### **3**·2H_2_O

To a suspension of **HL**^**2**^ (90 mg, 0.20 mmol) in isopropanol (30 mL) at 50 °C was added a solution of [RuCl_2_(*p*-cymene)_2_]_2_ (61 mg, 0.10 mmol) in chloroform (1.5 mL). The resulting mixture was stirred at 50 °C for 1 h. After cooling to room temperature about half of the solvent was removed under reduced pressure, and the reaction mixture was allowed to stand at 4 °C overnight. The next day the ocher-colored precipitate was filtered off and washed with isopropanol. Yield: 139 mg, 92%. Anal. Calcd for C_37_H_39_Cl_2_N_5_O_2_Ru·2H_2_O (*M*_r_ = 793.74), %: C, 56.46; H, 5.46, N, 8.82. Found, %: C, 56.69; H, 5.12; N, 8.88 ^1^H NMR (600 MHz, DMSO-*d*_6_) δ 12.50 (s, 1H, H^8^), 9.48 (s, 1H, H^17^), 9.03 (s, 1H, H^14^), 8.57 (t, *J* = 4.9 Hz, 1H, H^6^), 8.40 (s, 1H, H^12^), 8.25 (s, 2H, H^19^, H^20^), 8.06 (s, 1H, H^1^), 7.88 (d, *J* = 8.3 Hz, 1H, H^10^), 7.73 (d, *J* = 8.5 Hz, 1H, H^9^), 7.56 (t, *J* = 6.2 Hz, 1H, H^2^), 7.53 (d, *J* = 7.4 Hz, 1H, H^4^), 7.40 (t, *J* = 7.3 Hz, 1H, H^3^), 6.10 (d, *J* = 5.0 Hz, 1H, H^cy2^), 5.78 (d, *J* = 6.0 Hz, 1H, H^cy1^), 5.72 (d, *J* = 5.4 Hz, 1H, H^cy2^), 5.52 (d, *J* = 5.3 Hz, 1H, H^cy1^), 4.14 (s, 2H, H^5^), 3.81 (q, *J* = 13.8 Hz, 2H, H^21^), 3.64 (s, 4H, H^24^), 2.53 (m, 1H, H^cy3^; DMSO overlap), 2.46 (4H, H^23^; DMSO overlap), 2.22–2.11 (m, 3H, H^cy5^), 1.03–0.92 (m, 6H, H^cy4^). ^13^C NMR (151 MHz, DMSO-*d*_6_) δ 166.5 (CH, C^14^), 163.0 (Cq, C^7^), 156.6 (Cq, C^17^), 153.6 (CH, C^15^), 145.9 (Cq, C^11^), 139.6 (CH, C^19^), 139.5 (Cq, C^18^), 137.0 (Cq, C^4a^), 136.5 (Cq, C^8a^), 132.7 (Cq, C^12c^), 131.8 (Cq, C^7a^), 129.0 (CH, C^20^), 128.3 (CH, C^4^), 128.1 CH, C^2^), 127.3 (CH, C^1^), 126.8 (CH, C^3^), 123.9 (Cq, C^12a^), 120.3 (CH, C^10^), 116.9 (Cq, C^12b^), 113.9 (CH, C^12^), 113.2 (CH, C^9^), 104.7 (Cq, C^cy2a^), 102.8 (Cq, C^cy1a^), 86.8 (CH, C^cy2^), 85.3 (CH, C^cy1^), 66.0 (2 × CH_2_, C^24^), 58.2 (CH_2_, C^21^), 52.9 (2 × CH_2_, C^23^), 44.4 (CH_2_, C^5^), 30.6 (CH, C^cy3^), 21.9 (2 × CH_3_, C^cy4^), 18.1 (CH_3_, C^cy5^). ESI-MS (acetonitrile/methanol + 1% water), positive: *m*/*z* 713.24 [M – Cl]^+^.

#### **4**·H_2_O

To a suspension of **HL**^**2**^ (90 mg, 0.20 mmol) in isopropanol (30 mL) at 50 °C was added a solution of [OsCl_2_(*p*-cymene)_2_]_2_ (79 mg, 0.10 mmol) in chloroform (1.5 mL). The resulting mixture was stirred at 50 °C for 1 h. After cooling to room temperature about half of the solvent was removed under reduced pressure, and the reaction mixture was allowed to stand at 4 °C overnight. The next day the dark brown precipitate was filtered off and washed with isopropanol. Yield: 145 mg, 85%. Anal. Calcd for C_37_H_39_Cl_2_N_5_O_2_Os·H_2_O (*M*_r_ = 864.89), %: C, 51.38; H, 4.78; N, 8,10. Found, %: C, 51.14; H, 4.68; N, 7.90. ^1^H NMR (600 MHz, DMSO-*d*_6_) δ 12.51 (s, 1H, H^8^), 9.42 (s, 2H, H^14^, H^17^), 8.57 (t, *J* = 5.0 Hz, 1H, H^6^), 8.35 (s, 1H, H^20^), 8.33 (s, 1H, H^12^), 8.23 (d, *J* = 6.7 Hz, 1H, H^19^), 8.03 (s, 1H, H^1^), 7.77 (d, *J* = 8.7 Hz, 1H, H^10^), 7.72 (d, *J* = 8.7 Hz, 1H, H^9^), 7.57 (d, *J* = 12.3 Hz, 1H, H^2^), 7.54 (d, *J* = 7.3 Hz, 1H, H^4^), 7.40 (t, *J* = 7.3 Hz, 1H, H^3^), 6.39 (s, 1H, H^cy2^), 5.99 (d, *J* = 5.3 Hz, 1H, H^cy1^), 5.92 (d, *J* = 4.1 Hz, 1H, H^cy1^), 5.68 (s, 1H, H^cy2^), 4.13 (s, 2H, H^5^), 3.80 (q, *J* = 14.5 Hz, 2H, H^21^, solvent overlap), 3.64 (s, 2 × 2H, H^24^), 2.47 (s, 2 × 2H, H^23^, DMSO overlap), 2.41 (m, 1H, H^cy3^, DMSO overlap), 2.26 (s, 3H, H^cy5^), 0.93 (dd, *J* = 24.1, 5.3 Hz, 2 × 3H, H^cy4^). ^13^C NMR (151 MHz, DMSO-*d*_6_) δ 167.1 (CH, C^14^), 163.1 (Cq, C^7^), 155.3 (Cq, C^17^), 155.1 (CH, C^15^), 146.0 (Cq, C^11^), 140.5 (Cq, C^11^), 139.9 (CH, C^19^), 137.2 (Cq, C^4a^), 136.7 (Cq, C^8a^), 132.8 (Cq, C^12c^), 132.0 (Cq, ^C7a^), 129.0 (CH, C^20^), 128.4 (CH, C^4^), 128.8 (CH, C^2^), 127.4 (CH, C^1^), 126.9 (CH, C^3^), 124.0 (Cq, C^12a^), 120.8 (CH, C^10^), 117.0 (Cq, C^12b^), 114.5 (CH, C^12^), 113.4 (CH, C^9^), 96.7 (Cq, C^cy1a^), 96.3 (Cq, C^cy2a^), 79.2 (CH, C^cy2^), 77.3 (CH, C^cy1^), 76.1 (CH, C^cy2^), 75.6 (CH, C^cy1^), 66.1 (2 × CH_2_, C^24^), 58.1 (CH_2_, C^21^), 53.0 (2 × CH_2_, C^23^), 44.6 (CH_2_, C^5^), 30.7 (CH, C^cy3^), 22.2 (CH, C^cy4^), 21.6 (CH, C^cy4^), 18.2 (CH_3_, C^cy5^). ESI-MS (acetonitrile/methanol + 1% water), positive: *m*/*z* 812.24 [M – Cl]^+^.

ESI mass spectra as well as ^1^H and ^13^C NMR spectra are collected in Figures S1–S31.

### Additional Determination of Purity of Compound **1**

Reverse-phase (RP) HPLC analysis of compound **1** was carried out on a Shimadzu HPLC system equipped with a DGU-20A degasser, SPD-M20A UV detector, LC-20AB pump system, and a CBM-20A communication BUS module using RP-HPLC columns (Atlantis T3 C18, 5 μm, 4.6 × 250 mm^2^, P/N: 186003748). ESI mass spectra for compound **1** were recorded with a Waters Acquity UPLC (Milford, CA) with electrospray ionization SQ detector.

### Crystallographic Structure Determination

The measurements were carried out on a Bruker X8 APEXII CCD and Bruker D8 Venture diffractometers. Single crystals were positioned at 27, 27, and 30 mm from the detector, and 2899, 793, and 1260 frames were measured, each for 10, 60, and 10 s over 0.18, 0.5, and 1° scan width for **M**, **P**·H_2_O, and **1**·2DMF respectively. The data were processed using SAINT software.^37^ Crystal data, data collection parameters, and structure refinement details are given in Table 1. The structures were solved by direct methods and refined by full-matrix least-squares techniques. Non-H atoms were refined with anisotropic displacement parameters. H atoms were inserted in calculated positions and refined with a riding model. The following computer programs and hardware were used: structure solution, SHELXS and refinement, SHELXL;^38^ molecular diagrams, ORTEP;^39^ computer, Intel CoreDuo. Crystallographic data are available as CCDC 2109919 (**M**), 2109920 (**P**·H_2_O), and 2109921 (**1**·2DMF).

**Table 1:** Crystal Data and Details of Data Collection for **M**, **P**·H_2_O, and **1**·2DMF

compound	**M**	**P**·H_2_O	**1**·2DMF
empirical formula	C_24_H_25_N_3_O_6_	C_16_H_15_N_3_O_2_	C_38_H_44_Cl_2_N_6_O_3_Ru
fw	451.47	281.31	804.76
space group	triclinic, *P*1̅	monoclinic, *P*2_1_/*n*	orthorhombic, *Pna*2_1_
*a*, Å	9.8567(16)	4.0263(6)	23.5688(18)
*b*, Å	11.0047(18)	20.419(3)	9.7990(8)
*c*, Å	11.0179(19)	16.175(2)	16.0641(11)
α, deg	87.985(6)		
β, deg	78.250(6)	92.135(8)	
γ, deg	68.552(6)		
*V* [Å^3^]	1088.0(3)	1328.9(3)	3710.0(5)
*Z*	2	4	4
λ [Å]	0.71073	0.71073	0.71073
ρ_calcd_, g cm^–3^	1.378	1.406	1.310
crystal size, mm^3^	0.20 × 0.20 × 0.15	0.09 × 0.08 × 0.02	0.08 × 0.06 × 0.01
*T*, K	138(2)	140(2)	120(2)
μ, mm^–1^	0.100	0.095	0.602
*R*_1_^a^	0.0408	0.0562	0.0660
*wR*_2_^b^	0.1015	0.1313	0.1833
GOF^c^	1.006	1.096	1.084

a *R*_1_ = Σ||*F*_0_| – |*F*_c_||/Σ|*F*_0_|.

b *wR*_2_ = {Σ[*w*(*F*_0_^2^ – *F*_c_^2^)^2^]/Σ[*w*(*F*_0_^2^)^2^]}^1/2^.

c GOF = {Σ[*w*(*F*_0_^2^ – *F*_c_^2^)^2^]/(*n* – *p*)}^1/2^, where *n* is the number of reflections and *p* is the total number of parameters refined.

### Cell Lines and Culture Conditions

The human breast adenocarcinoma MDA-MB-231 cell line was purchased from ATCC. The human hepatocellular carcinoma LM3 (HCCLM3) cell line was kindly offered by Prof. Kan Man Hui (Singapore), and the human glioma cell line U-87 MG was offered by Dr. Tan Boon Toh (Singapore). All cells were cultured in DMEM (with high glucose, 4.0 mM l-glutamine, without sodium pyruvate (Hyclone)) containing 10% fetal bovine serum (FBS; Cytiva HyClone Fetal Bovine Serum, South American Origin) and 1% pen/strep (ThermoFisher). Adherent cells were grown in T-75 flasks (Greiner Bio-One). All cell lines were grown at 37 °C in a humidified atmosphere of 95% air and 5% CO_2_. Experiments were carried out on cells within 30 passages. The amount of actual Ru concentration in the stock solutions was determined by ICP-OES.

### Inhibition of Cell Viability Assay

The cytotoxicity of the compounds was determined by colorimetric microculture assay (MTT assay). The cells were harvested from culture flasks by trypsinization and seeded into Cellstar 96-well microculture plates (Greiner Bio-One) at the seeding density of 6 × 10^4^ cells/well. The cells were allowed to resume exponential growth for 24 h, then exposed to drugs at different concentrations in media for 72 h. All drug stock solutions were prepared in DMSO and further diluted with complete medium so that the final concentration of DMSO in medium did not exceed 1% (*v*/*v*) at which cell viability was not inhibited.^40,41^ Subsequently, 100 μL aliquots of the drug solution at eight concentrations were added to each well. After exposure for 72 h, drug solutions were replaced with 100 μL of 3-(4,5-dimethylthiazol-2-yl)-2,5-diphenyltetrazolium bromide (MTT) in media (5 mg mL^–1^) and incubated for additional 75 min. Subsequently, the medium was aspirated, and the purple formazan crystals formed in viable cells were dissolved in 100 μL of DMSO per well. Optical densities were measured at 570 nm with a microplate reader. The quantity of viable cells was expressed in terms of treated/control (T/C) values by comparison to untreated control cells, and 50% inhibitory concentrations (IC_50_) were calculated from concentration-effect curves by interpolation using GraphPad Prism software (version 5.01). The evaluation was based on averages from at least three independent experiments, each comprising three replicates per concentration level.

### Tubulin Polymerization Assay by Western Blotting

MDA-MB-231 cells were seeded into Cellstar 6-well plates (Greiner Bio-One) at a density of 3 × 10^5^ cells/well. After the cells were allowed to resume exponential growth for 48 h, they were exposed to compounds of interest at different concentrations for 12 h (6 wells per sample). Subsequently, cells were washed 3 times with ice-cold phosphate-buffered saline (PBS) and 100 μL of cold hypotonic buffer (1 mmol/L MgCl_2_, 2 mmol/L EGTA, 0.5% (*v*/*v*) Shell Nonidet P-40, and 50 mmol/L Tris–HCl (pH 6.8), supplemented with Pierce Protease and Phosphatase Inhibitor Tablet (1 unit per 10 mL of hypotonic buffer) were added directly into plates and incubated at 37 °C for 5 min. Then plates were placed on the ice and scraped with cell scrapers. To increase protein yield, the lysates from 6 wells were combined together in 1.5 mL Eppendorf microtubes, briefly mixed (Vortex Genie mixer, setting 10), and centrifuged at 14 400 × *g* for 10 min at 4 °C. After centrifugation, supernatants containing soluble (depolymerized) tubulin fraction were carefully separated from the pellet with polymerized tubulin. Pellets were subsequently lysed with modified RIPA buffers (10 μL of modified RIPA buffer (150 mM NaCl, 0.5% sodium deoxycholate, 2.0% IGEPAL CA-630, 0.5% sodium deoxycholate, 0.4% SDS, 50 mM Tris, Pierce Protease and Phosphatase Inhibitor Tablet (1 unit per 10 mL of RIPA buffer) with 2 μL of Invitrogen TURBO DNase (2 U/μL)) and resuspended until the pellets were fully dissolved. Then an additional 50 μL of modified RIPA buffer (150 mM NaCl, 0.5% sodium deoxycholate, 2.0% IGEPAL CA-630, 0.5% sodium deoxycholate, 0.4% SDS, 50 mM Tris, pH 8.0, 5 mM of EDTA (pH 8.0), Pierce Protease and Phosphatase Inhibitor Tablet (1 unit per 10 mL of RIPA buffer)) was added and resuspended few times and mixed on Vortex Genie mixer, setting 10. After pellets were completely dissolved, the pellet lysates were centrifuged at 14400 × g at 4 °C for 10 min and then the supernatants were carefully placed in the separate tube (these lysates are further referred to as a “polymerized tubulin” fraction). The protein content in both soluble and polymerized fractions was determined by BCA assay using Thermo Scientific Pierce BCA Protein Assay Kit. Equal quantities of proteins (15 μg) were reconstituted in loading buffer 4× Laemmli Buffer [0.4 M DDT SDS 8.0% (*w*/*v*), bromophenol blue 6 mM, glycerol 4.4 M] and heated at 95 °C for 5 min. Subsequently, the protein mixtures were resolved on a 10% SDS-PAGE gel by electrophoresis (90 V for 30 min followed by 120 V for 60 min) and transferred onto a 0.45 μm nitrocellulose membrane (100 V const., 350 mA for 1 h). The protein bands were visualized with Ponceau S stain solution, and the nitrocellulose membranes were cut into strips based on the protein ladder. The membranes were washed with TBST wash buffer (0.1% Tween-20 in 1× TBS) three times for 5 min. Subsequently, they were blocked in 5% (*w*/*v*) BSA in wash buffer for 1 h and subsequently incubated with the appropriate primary antibodies in 5% BSA (*w*/*v*) in TBST wash buffer at 4 °C overnight. The membranes were washed with a wash buffer 3 times for 5 min. After incubation with horseradish peroxidase-conjugated secondary antibodies (RT, 2 h), the membranes were washed with a wash buffer 3 times for 5 min. Immune complexes were detected with Immobilon Crescendo Western HPR substrate (Millipore) and analyzed using chemiluminescence imaging machine (ChemiDoc Touch Imaging System, BioRad). Total Protein staining by Coomassie Brilliant Blue G-250 was used as a loading control. The following antibodies were used: anti-α-tubulin (DM1A) mouse monoclonal antibodies (no. 3873 (1:2500)) and anti-mouse IgG HRP-linked antibody (1:5000) from Cell Signaling Technology. Experiments were repeated at least 3 times. Normalized densitometric values of the Western Blot images were obtained using ImageLab 6.1 software (Bio-Rad). Values are expressed as *P*% (percent of the polymerized tubulin within the total tubulin fraction) and represent mean ± SD.

### Fluorescence Microscopy

Coverslips were soaked in 95% ethanol for 10 min and gently placed inside 6-well culture plates containing 1× PBS (1 mL). The PBS was aspirated, and the plates were washed with 1× PBS (1 mL) one more time. After the coverslips were dried, gelatin (0.2%, 400 μL) which was prewarmed to 37 °C was carefully added on top of the coverslips and incubated (37 °C, 5% CO_2_) for 30 min. Then gelatin was removed, and the coverslips were washed twice with 1× PBS (2 × 1 mL), dried, and exposed to UV light for 15 min. Subsequently, gelatin-coated coverslips were placed into each well of a 6-well culture plate (Greiner Bio-One) and MDA-MB-231 cells were seeded onto gelatin-coated coverslips at a density of 1.5 × 10^5^ cells/mL (1 mL per well). After the cells were allowed to resume exponential growth for 24 h, the cell culture medium was aspirated and replaced with the drug solutions in media at the desired concentrations. The cells were then incubated with drug solutions for 12 h in cell culture incubator (37 °C, 5% CO_2_) in the darkness. After the drug solutions were aspirated and the coverslips were washed with 1× PBS (2 × 1 mL), cells were fixed with PFA solutions diluted in 1× PBS (4%, 800 μL) for 20 min and carefully removed. Permeabilization buffer (0.1% Tween 20 in 1× PBS, 800 μL) was prewarmed to 37 °C, gently added to each well, and incubated at room temperature for 15 min. The buffer was aspirated and washed with 1× PBS (3 × 1 mL), followed by the addition of blocking buffer (5% BSA in 1X PBS-T, 800 μL), which was incubated at room temperature for 45 min. After the removal of blocking buffer, primary antibody (CST anti-α-tubulin (DM1A) mouse mAb, diluted at 1:2000 in blocking buffer, 700 μL) was added and incubated at 4 °C overnight. The coverslips were washed with 1× PBS-T (3 × 1000 μL) using a shaker on low speed for 5 min. Secondary antibody (Thermo Fisher Alexa Fluor 488-conjugated secondary Ab anti-mouse IgG, diluted at 1:1000 in blocking buffer, 700 μL) was added and incubated at room temperature in darkness for 1.5 h, after which the coverslips were washed by using a procedure similar to that in the previous step. DAPI (300 nM in 1× PBS, 350 μL) solution was carefully added to ensure complete coverage of cells for 10 min to counterstain the nucleus. The coverslips were washed with 1× PBS (3 × 1 mL), and the remaining liquid was carefully removed using the edge of KimWipes. Mounting solution (ProLong Gold Antifade Mountant, 1 drop) was used to mount coverslips on to microscope glass slides and cured at 4 °C overnight. The samples were protected from photodegradation by covering with aluminum foil prior to imaging. Fluorescence images were obtained using a fluorescein isothiocyanate (FITC) or DAPI filter with a Nikon Monochrome Camera Qi1Mc via a 100× oil immersion objective. The images were processed and analyzed using the NIS Elements BR imaging software (version 5.30.03)

### Pilot *In Vivo* Experiments

Animal studies were conducted in compliance with the protocols approved by the International Animal Care and Use Committee (IACUC) of Memorial Sloan Kettering Cancer Center (MSKCC). Nude athymic mice (Hsd:Athymic Nude-Foxn1^nu^, 6–8 weeks old) were purchased from Envigo U.S. The mice were randomized into 6 groups (*n* = 3 per group) and intravenously (i.v.) injected with 1, 5, 10, 15, 20, or 25 mg/kg of complex **1** in 100 μL of PEG-300/PBS (4% *v*/*v*). For the experiments with MDA-MB-468 tumors, 100 μL of 1:1 L-15 media/Matrigel (Corning, NY) containing 5 million cells was subcutaneously injected at the right shoulder of 12 mice. Animals were randomly assigned into two groups when the tumors reached a size of ca. 100 mm^3^. The treatment group was administered a single intravenous injection of 10 mg/kg **1** in 100 μL of 4% PEG-300/PBS. The control group was administered 100 μL of the vehicle (PEG-300/PBS 4%, *v*/*v*). For the experiments with LX22 tumor cells, 100 μL of 1:1 L-15 media/Matrigel (Corning, NY) containing 2.5 million cells was subcutaneously injected at the right shoulder of 8 mice. Animals were randomly assigned into two groups after 1 week. Treatment was carried out every other day by intravenous injection of 7.5 mg/kg **1** in 100 μL of 4% PEG-300/PBS. The control group was similarly administered 100 μL of the vehicle (PEG-300/PBS, 4% *v*/*v*). For both experiments, the mice were monitored every day. Their weights were recorded daily, and tumor size was manually measured with a caliper. At the end point, mice were euthanized based on study time line criteria, weight loss (>10% of preinjection weight), moribund appearance/behavior, a tumor size of 1000 mm^3^, or necrosis.

All procedures apart from tail vein injection were conducted under 2.5% isoflurane inhalation anesthesia in 2.5 L medical air flow.

## Results and Discussion

### Synthesis

11-Nitro-substituted indole-fused latonduine derivatives were prepared by following recently published synthesis pathways.^14,16^ However, due to the low solubility of intermediate compounds **J**–**N**, with NO_2_ group as substituent in the indole ring, in a variety of solvents, the protocols previously developed for analogous unsubstituted and bromo-substituted intermediates^14,16^ had to be adapted. With the alterations carried out, yields were maintained in the ranges reported for other derivatives.^14,16^ First, protection of the starting ethyl 5-nitro-indole-2-carboxylate with ethoxymethyl chloride afforded species **J** in almost quantitative yield. Subsequently, **J** was hydrolyzed using lithium hydroxide followed by acidic work up and recrystallization from ethanol to give **K** in 92% yield (Scheme 4). Amide coupling of species **K** with 2-iodobenzylamine^14,16^ to carbamide **L** was carried out in dry chloroform in the presence of EDCI·HCl and DMAP in 92% yield. Pure carbamide **L** precipitated overnight from the reaction mixture was isolated after washing with diethyl ether with excellent purity. Boc-protection of the amide nitrogen in **L** delivered **M** in 87% yield. Intramolecular Heck-cyclization of **M** was carried out in dry DMF in the presence of Pd(OAc)_2_, PPh_3_ and Ag_2_CO_3_ in 1:2:10 molar ratio at 100 °C affording crystalline **N** in 93% yield. Complete deprotection of indole-fused latonduine **N** was realized with 12 M HCl in ethanol (1:4) to give **O** in 60% yield after chromatographic separation from starting material on silica. When the deprotection reaction was carried out in dioxane/1 M HCl as reported for Br-substituted and unsubstituted derivatives,^14,16^ only cleavage of the Boc protection group was found, while EOM remained intact. Further reduction of the nitro group was attempted by using iron powder under acidic conditions.^42^ However, no product was obtained, likely due to the generation of an iron complex, which could be observed by the appearance of its typical red color. In contrast, reduction with hydrogen in the presence of Pd/C catalyst^43−45^ afforded desired product **P**. By careful optimization of the procedure published by Lu et al.^45^ pure amine **P** could be isolated in quantitative yield. This latter compound crystallized upon concentration of the solution to give X-ray diffraction quality crystals. Attempts failed to accomplish deprotection and reduction in one step by using iron powder or stannous chloride in acidic medium as reported in the literature.^42,46^

The following Schiff-base reactions of amine **P** with 2-formylpyridine and its 5-methylmorpholinyl derivative were conducted by following a protocol explored previously for related compounds.^14,27^ Attempts to perform the Schiff base condensation reactions of **P** with 2-acetylpyridine under the same conditions failed, perhaps due to the steric hindrance induced by the methyl group and hydrogen atoms of the indole ring which is strong enough to hinder the formation of a new imine bond. Organoruthenium(II) and -osmium(II) complexes **1**–**4** were synthesized by straightforward reactions of **HL**^**1**^ and **HL**^**2**^ with [M^II^Cl_2_(η^6^-*p*-cymene)], where M = Ru and Os, in isopropanol/chloroform in 71–98% yields. Spectroscopic characterization data (^1^H and ^13^C NMR, ESI-MS, and UV–vis) are presented in the Supporting Information. Complexes **1**–**4** are chiral at the metal(II) center. Since other chiral centers are missing, the two enantiomers likely coexist in solution as a racemic mixture. Further evidence is provided by single-crystal X-ray diffraction of **1**·2DMF.

### X-ray Crystallography

The results of X-ray diffraction studies of species **M**, **P**, and **1**·2DMF are shown in Figures S32, S33, and 1, respectively. The complex crystallized as a racemate in the noncentrosymmetric orthorhombic space group *Pna*2_1_ and adopted the typical three-leg piano-stool geometry. Bidentate ligand **HL**^**1**^ coordinated to ruthenium via nitrogen atoms N13 and N20, and the chlorido coligand were the legs. The *p*-cymene acted as the piano’s stool, in the same manner as reported previously for a similar complex with a paullone core.^47^ The nonplanarity of the latonduine core was due to the presence of one sp^3^-hybridized methylene group in the seven-membered azepinone ring. The latonduine core formed with the bidentate moiety coordinated to ruthenium a dihedral angle of 57° to avoid steric collision with the *p*-cymene moiety.

**Figure 1:**
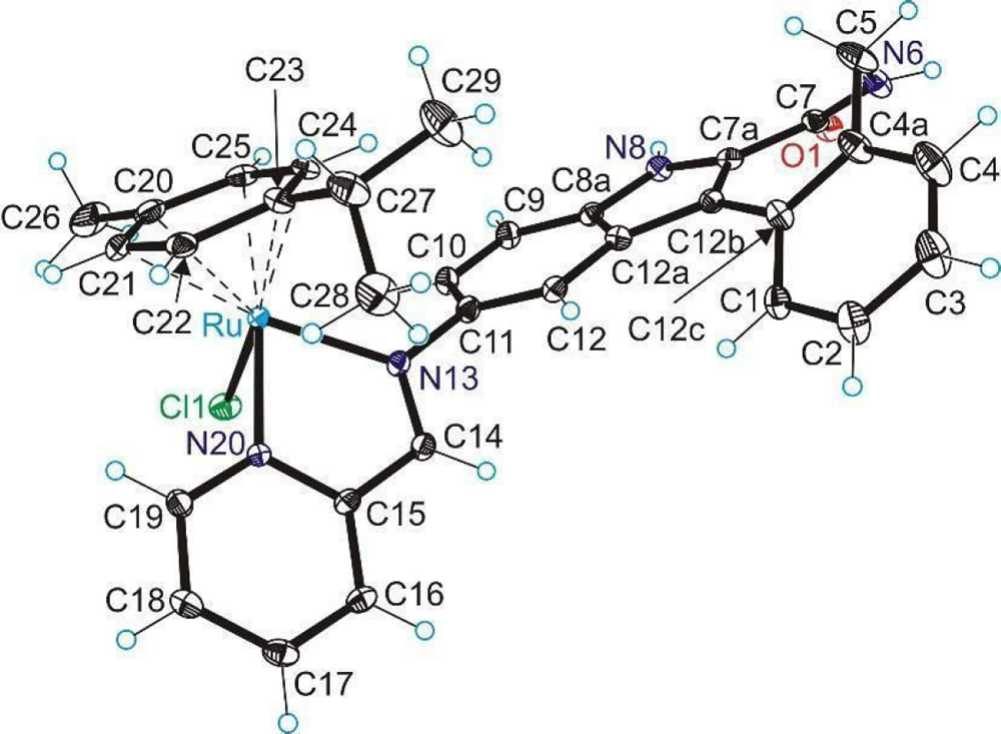
ORTEP view of the complex cation [Ru^II^Cl(η^6^-*p*-cymene)(HL^1^)]^+^ in **1**·2DMF with thermal ellipsoids at 30% probability level. Selected bond distances (Å), bond angles (deg), and dihedral angles: Ru–N13 2.072(4), Ru–N20 2.094(3), Ru–Cl1 2.3900(11), Ru–C20 2.238(5), Ru–C21 2.205(5), Ru–C22 2.159(4), Ru–C23 2.213(5), Ru–C24 2.172(4), Ru–C25 2.186(4), Ru–*p*-cymene ring centroid 1.6852(17); N13–Ru–N20 76.96(14), N13–Ru–Cl1 86.59(11), N20–Ru–Cl1 84.20(10); Θ_Ru–N13–C11–C10_ 57.3(5).

### Molecular Descriptors

By using the online pharmacokinetic program SwissADME^48^ some physicochemical parameters, lipophilicity, and aqueous solubility have been calculated and summarized in Table S1, along with experimentally determined solubility and pH values of aqueous solutions containing DMSO of the two ligands and four complexes. Organic ligands **HL**^**1**^ and **HL**^**2**^, in contrast to complexes **1**–**4**, have molecular weight values of ≤500 g/mol, and according to Lipinski’s rules^49^ are good candidates to accurately assess their drug-likeness. These values are also in agreement with their facility to cross the plasmatic membrane and reach the intracellular environment. All the compounds reported in this work have Log *P* parameters of less than 5, which is related to hydrophobicity of a given molecule.^49^ The number of hydrogen donors and the number of hydrogen bond acceptors for the synthesized compounds are less than 5 and less than 10, respectively, also in accordance with Lipinski’s rules. Aqueous solubility (Log *S*) is an important parameter for absorption and distribution of a drug in the organism. The Log *S* values (determined by the topological method)^50^ indicate that this algorithm predicts to some extent the solubility of organic compounds **HL**^**1**^ and **HL**^**2**^, but it failed to predict the aqueous solubility of metal complexes **1**–**4**. The latter can be classified as insoluble based on calculations, but the compounds are in fact soluble in aqueous solutions containing 1% DMSO (see Table S1). The measured pH values for complexes **1**–**4** in 1% DMSO/H_2_O were slightly acidic, while those (uncorrected) for **HL**^**1**^ and **HL**^**2**^ in DMSO/H_2_O 1:1 were slightly basic at room temperature (Table S1).

### Stability Studies

Prior to the *in vitro* experiments, we carried out kinetic studies by ^1^H NMR and UV–vis spectroscopy to ensure the stability of the compounds in solvent media. Since the compounds of interest required the addition of 1% DMSO into the aqueous cell culture medium, the stabilities of **HL**^**1**^, **1**, and **2** in DMSO-*d*_6_ were monitored for 96 h by ^1^H NMR spectroscopy (Figures S34–S36). No changes in the spectra were observed over the whole time period, indicating high stability of the tested compounds in DMSO. It should be noted that DMSO-assisted exchange of chlorido- or N-coordinated ligands is fairly common in half-sandwich Ru and Os complexes and that coordination of DMSO to a metal center is characterized by a significant downfield shift from 2.5 ppm for free DMSO to 2.8–3.0 for *S*- or *O*-coordinated DMSO ligands,^51,52^ as well as by the appearance of a new set of signals corresponding to arene fragment.^53^ As can be seen from Figures S35 and S36, no such changes were observed over 96 h, indicating the high stability of the ligands toward DMSO substitution. In addition, we carried out ^1^H–^15^N HMBC experiments, the results of which are shown in Figure S37. These results are in line with the ^1^H and ^13^C NMR spectra of **1** indicating that coordination of bidentate ligand **HL**^**1**^ to Ru(II) was preserved in solution (DMSO-*d*_6_) and was realized via nitrogen atoms N13 and N16. Upon coordination, we observed an upfield shift of the ^15^N resonances of N13 and N16 in **HL**^**1**^ from 337.1 and 318.4 ppm, respectively, to 275.1 and 246.0 ppm in **1**. These atoms revealed long-range couplings to the hydrogen atoms H14 and H17 both in **HL**^**1**^ and **1**. Similar upfield shifts were observed upon coordination of nitrogen donor atoms to transition metals.^54^

For ligand **HL**^**1**^, hydrolysis kinetics were analyzed in DMSO/D_2_O 1:1 and monitored by ^1^H NMR spectroscopy. Again, no changes were observed indicating that Schiff base **HL**^**1**^ remained intact under these conditions (see Figure S38). Next, we assessed the stabilities of complexes **1** and **2** in 1% DMSO/H_2_O for 72 h using UV–vis spectroscopy. Complexes **1** and **2** did not show any changes in the UV–vis spectra over 72 h (Figures S39 and S40). The ^1^H NMR spectrum of **1** in 5% DMSO-*d*_6_/D_2_O also remained unchanged over 12 h (Figure S41). To verify UV–vis data, the stability of complex **1** was additionally tested by analytical HPLC-HR-MS using a 5–95% acetonitrile/water gradient with 0.1% formic acid over a period of 15 min. A single peak at around 10 min corresponding to the [**1** – Cl]^+^ peak (*m*/*z* = 623.12) was observed (Figure S42) in agreement with other experiments. Therefore, we assumed that all compounds of interest did not undergo any transformations in aqueous solutions.

### Anticancer Activity

On the basis of our hypothesis that the anticancer mechanism of action of novel latonduine derivatives with the indole fragment might be related to the microtubule targeting and the inhibition of mitosis, we have chosen several cancer cell lines, namely, breast cancer cell line MDA-MB-231, hepatocellular carcinoma cell line LM3, and glioma cell line U-87 MG. These cancer types are characterized by especially aggressive behavior in patients and high rates of metastasis and proliferation.^55−57^ Therefore, these cell lines might be suitable models for the anticancer agents targeting the process of cell division. The *in vitro* anticancer activities of latonduines **HL**^**1**^ and **HL**^**2**^ and their respective Ru^II^ and Os^II^ complexes, **1**–**4**, were determined by the colorimetric MTT assay with an exposure time of 72 h. The IC_50_ values of compounds of interest are listed in Table 2, and the concentration–effect curves are depicted in Figure S43.

**Table 2:** Cytotoxicity of Latonduine Derivatives **HL**^**1**^ and **HL**^**2**^ and Corresponding Ru^II^ and Os^II^ Complexes in Comparison with Cisplatin, Sorafenib, and Tubulin-Targeting Drug Paclitaxel (Taxol)

			IC_50_ [μM]^a^	
compound	type	MDA-MB-231	LM3	U-87 MG
**HL**^**1**^	ligand	1.4 ± 0.4	5.6 ± 1.5	10 ± 4
**1**	Ru^II^	57 ± 9	113 ± 13	176 ± 26
**2**	Os^II^	82 ± 22	126 ± 41	144 ± 12
**HL**^**2**^	Ligand	1.4 ± 0.4	4.5 ± 1.8	>10
**3**	Ru^II^	90 ± 15	118 ± 32	>160
**4**	Os^II^	140 ± 19	215 ± 48	>250
cisplatin		21 ± 5	10 ± 3	n.d.^b^
sorafenib		n.d.	7.4 ± 2.7	n.d.
paclitaxel		0.006 ± 0.001	n.d.	n.d.

a The 50% inhibitory concentration (IC_50_) in human breast adenocarcinoma cell line MDA-MB-231, human hepatocellular carcinoma cell line LM3 (HCCLM3), and human glioma cell line U-87 MG was determined by the MTT assay after exposure for 72 h. The values are the means ± SD obtained from at least three independent experiments.

b n.d., not detected.

Both latonduine derivatives **HL**^**1**^ and **HL**^**2**^ demonstrated excellent cytotoxicity in all tested cell lines in a low micromolar range. It is believed that the presence of a morpholine fragment in the backbone of drug candidates might be associated with favorable pharmacological properties and improved biological activity.^26−29^ Herein, the incorporation of a morpholine fragment did not result in any significant differences in cytotoxicity of **HL**^**1**^ and **HL**^**2**^ in all tested cell lines. Both metal-free ligands were 2- to 15-fold more cytotoxic than cisplatin. However, in clinics cisplatin is usually not used for the treatment of these tumor types. To ensure a fair comparison of the novel compounds with clinically relevant drugs, we additionally tested the cytotoxicity of the microtubule-stabilizing drug paclitaxel (Taxol) in MDA-MB-231 and sorafenib in LM3, which are clinically used drugs for triple-negative breast cancer^58,59^ and hepatocellular carcinoma,^60,61^ respectively. It was revealed that the cytotoxicities of **HL**^**1**^ and **HL**^**2**^ were comparable to sorafenib, while the cytotoxicities of **HL**^**1**^ and **HL**^**2**^ were several orders of magnitude lower than that of paclitaxel. However, the nanomolar activity of paclitaxel is typically translated into a marked cytotoxicity in cancer patients, associated with sometimes unbearable side effects.^62,63^ In contrast to **HL**^**1**^ and **HL**^**2**^, their corresponding Ru^II^ and Os^II^ complexes demonstrated significantly lower cytotoxicities than those of cisplatin and sorafenib. In general, the coordination to Ru^II^ resulted in up to 40-fold decrease of cytotoxicity, while Os^II^ complexes were even less cytotoxic. We questioned whether the excellent anticancer activity of indole-derived latonduine derivatives was indeed related to the microtubule targeting and whether the coordination to metal centers resulted in the possible alteration of this mechanism of action. Therefore, we carried out a detailed investigation of microtubule targeting abilities of proligand **HL**^**1**^ and complexes **1** and **2** in MDA-MB-231 cells in comparison with known tubulin-targeting compounds.

### Investigation of Tubulin Targeting by Immunocytochemistry

**Figure 2:**
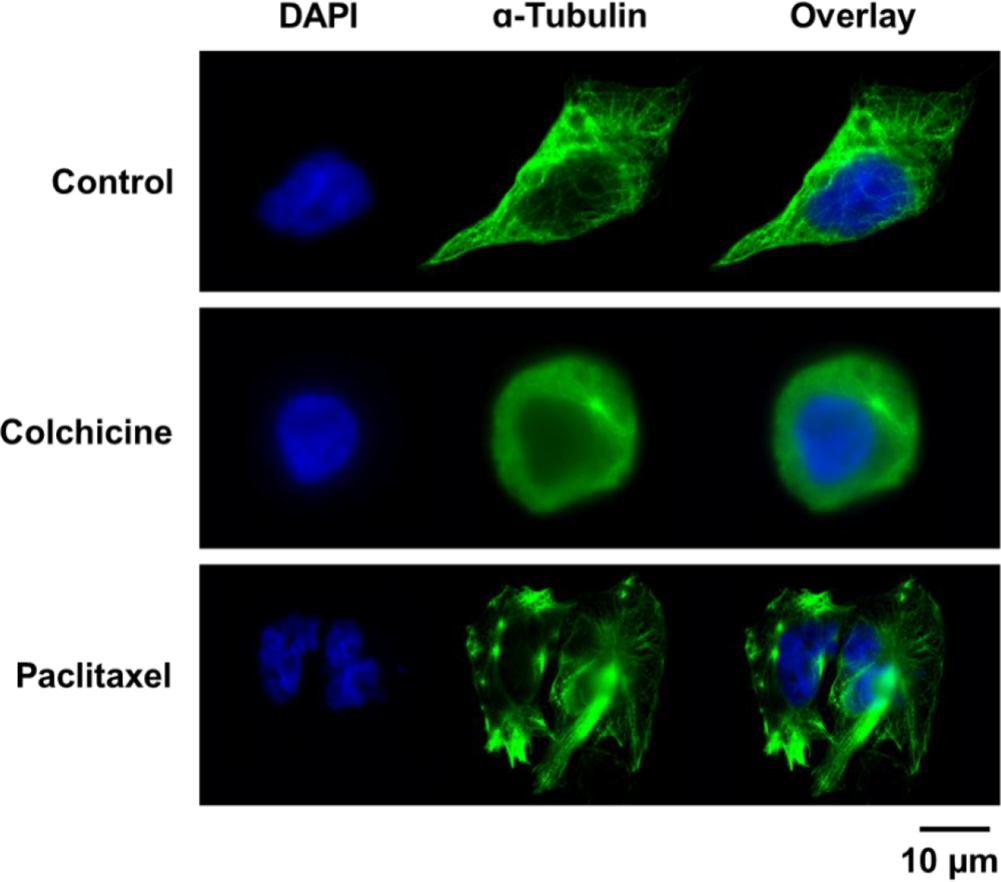
Fluorescence microscopy images of MDA-MB-231 cells treated with either paclitaxel (50 nM) or colchicine (50 nM) for 12 h. Cells were fixed and nuclei were stained with DAPI (blue), while tubulin was stained with Thermo Fisher Alexa Fluor 488-conjugated secondary antibody (green).

To visualize the microtubule polymerization status in drug-treated MDA-MB-231 cells, we carried out fluorescence imaging of α-tubulin. Initially, the studies were carried out with paclitaxel and colchicine, which are well-known microtubule-stabilizing and microtubule-destabilizing agents, respectively (Figure 2). The results were in a good agreement with those reported in the literature.^64,65^ While untreated cells demonstrated a pronounced microtubule network with a highly organized structure, treatment with the microtubule-destabilizing agent colchicine has led to a spherical cellular morphology with no defined microtubules. In addition, the intensity of α-tubulin staining in the colchicine-treated cells was greatly reduced. In contrast, in cells treated with the microtubule-stabilizing agent paclitaxel, microtubules were characterized by bright staining, multipolarity spindles, and thickly structured bundles.

**Figure 3:**
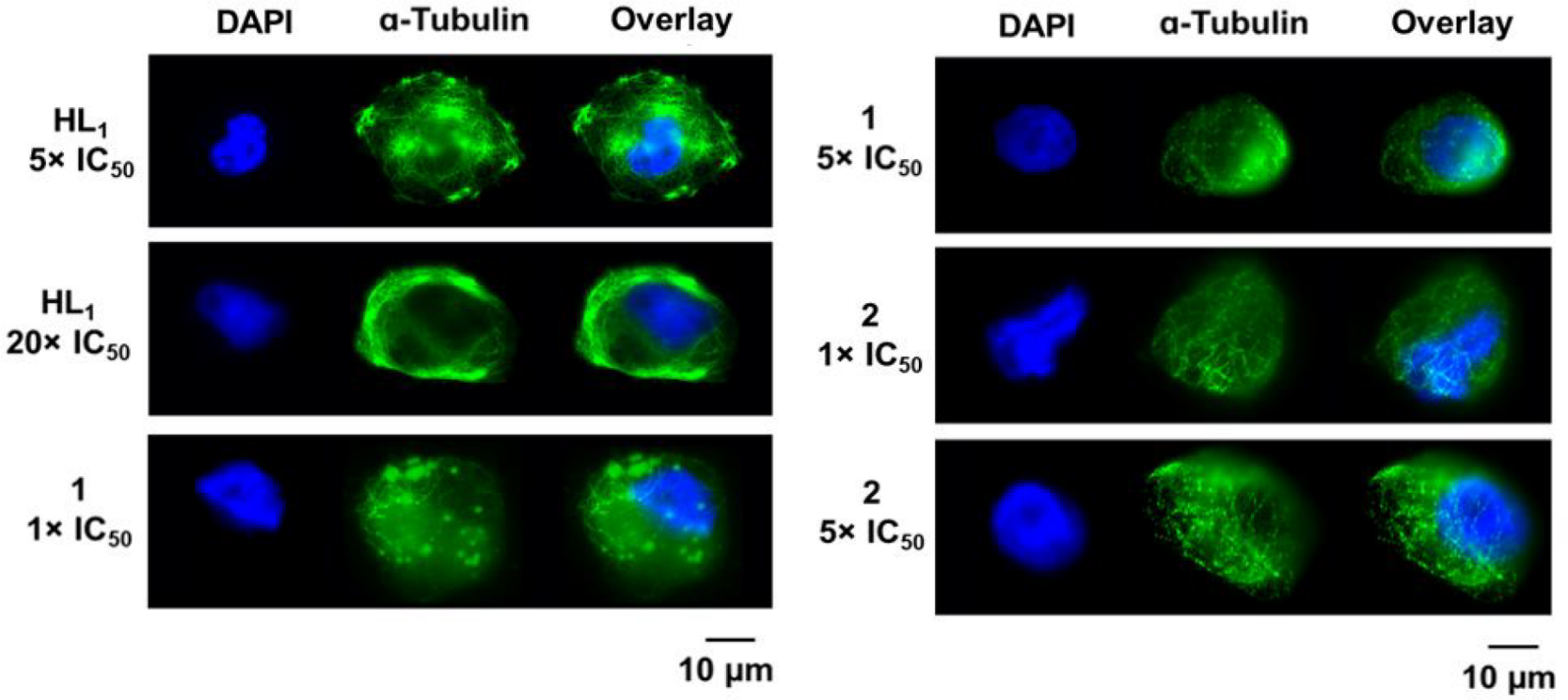
Fluorescence microscopy images of MDA-MB-231 cells treated for 12 h with **HL**^**1**^ (at 5 × IC_50_ and 20 × IC_50_ values obtained from the MTT experiment with 72 h exposure) and complexes **1** and **2** (at 1 × IC_50_ and 5 × IC_50_ values obtained from the MTT experiment with 72 h exposure). Cells were stained for nucleus (DAPI, blue), while tubulin is stained with Thermo Fisher Alexa Fluor 488-conjugated secondary antibody (green).

Subsequently, we monitored the dose-dependent tubulin-targeting effects of **HL**^**1**^, **1**, and **2**. MDA-MB-231 cells were treated with **HL**^**1**^ (at concentrations corresponding to 5 × IC_50_ and 20 × IC_50_ values) and complexes **1** and **2** (at concentrations corresponding to 1 × IC_50_ and 5 × IC_50_ values) for 12 h (Figure 3). The fluorescence images revealed the tubulin-targeting properties of all tested compounds reflected by the disrupted organization of microtubule network in agreement with the initial hypothesis. Cells treated with **HL**^**1**^ demonstrated bright patchy bundles, similar to paclitaxel,^66^ as well as the loss of cell morphology. Cells treated with complexes **1** and **2** also demonstrated the loss of cell morphology and disruption of the organized microtubule network, suggesting a microtubule-destabilizing behavior. On the basis of these images, we were not able to unambiguously confirm the exact mechanism of microtubule interference by **HL**^**1**^. Therefore, we subsequently investigated its effects on tubulin polymerization using Western blotting.

### Tubulin Polymerization Analyzed by Western Blotting

The *in vitro* tubulin polymerization assay is based on the fractionation of depolymerized (soluble) and polymerized α-tubulin fractions of drug-treated cells in hypotonic buffer conditions with their subsequent analysis by Western blotting.^33^ The hypotonic buffer was reported to keep assembled microtubules in a polymerized state and depolymerized tubulin in a soluble form. Therefore, these lysis conditions allow for the preservation of the degree of tubulin polymerization in drug-treated cells.^28^ Following the lysis, the supernatant fraction containing soluble tubulin and the pellet fraction containing polymerized tubulin should be separated by centrifugation and quantified. According to the published experimental procedures, the polymerized fraction was expected to be redissolved in hypotonic buffer prior to quantification.^67−69^ However, in our case the solubility of pellet fractions was very poor. In order to achieve correct quantification of protein content in polymerized fractions, we modified the experimental conditions and lysed polymerized tubulin pellets in RIPA buffer containing a DNase enzyme, which fully dissolved the pellet fraction. Subsequently, both soluble and polymerized fractions were quantified and resolved using gel electrophoresis. It should be noted that several tubulin-targeting agents were reported to affect actin and GAPDH proteins, which are typically used in Western blotting experiments as loading controls. Hence, we ensured equal loading using total protein staining with highly sensitive Coomassie Brilliant Blue dye.^70^

**Figure 4:**
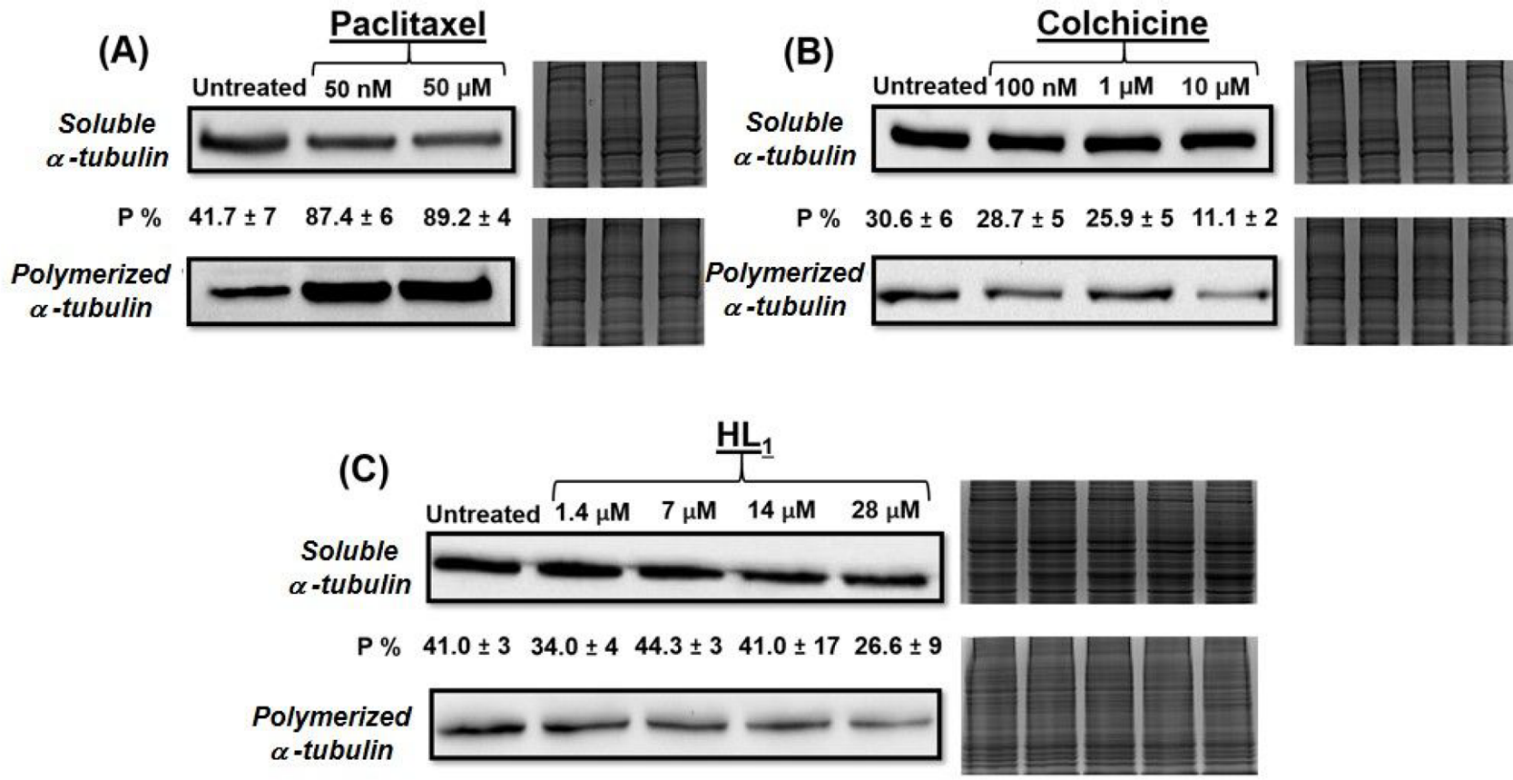
Western Blot analysis of α-tubulin protein in soluble and polymerized cell fractions. MDA-MB-231 cells were collected after incubation with compounds of interest at indicated concentrations for 12 h. Cells were lysed in a hypotonic buffer, and soluble and polymerized fractions were separated by centrifugation. The polymerized fraction was dissolved in RIPA buffer with DNase enzyme. The protein content was quantified by BCA assay, and equal amounts of proteins were resolved by gel electrophoresis. Soluble α-tubulin corresponds to the monomeric tubulin from supernatants. Polymerized α-tubulin corresponds to the polymerized tubulin fraction from pellets. The corresponding total protein staining using Coomassie Brilliant Blue G-250 dye represents the loading control. Semiquantitative band density analysis was carried out via ImageLab analysis. *P*% values represent a percentage of densitometric density of the polymerized α-tubulin compared to the total α-tubulin fractions (sum of the soluble (depolymerized) and polymerized α-tubulin fractions) normalized by the loading control. Each *P*% value is an average of at least three independent experiments and represents mean ± SD.

Initially, we verified the optimized experimental conditions using paclitaxel and colchicine. In the literature, the effects of paclitaxel and colchicine in cancer cells, including MDA-MB-231 cells, at 12 h incubation time, were demonstrated for a wide range of concentrations from low nanomolar to high micromolar. To demonstrate dose-dependent tubulin polymerization, we used colchicine in the concentration range between 100 nM and 10 μM (Figure 4A) and paclitaxel between 50 nM and 50 μM (Figure 4B). On the basis of its proposed mechanism of action, paclitaxel was expected to increase the fraction of polymerized tubulin (microtubule stabilization) and decrease the fraction of soluble tubulin, while colchicine was expected to act in an opposite manner (i.e., microtubule-destabilizing).^64,65^ Changes in the fractions were measured by semiquantitative densitometry. As expected, paclitaxel demonstrated a ca. 2-fold increase in polymerized tubulin fraction even at 50 nM (10 × IC_50_), while colchicine demonstrated a ca. 3-fold decrease of polymerized tubulin fraction at 10 μM (ca. 700 × IC_50_). Subsequently, we investigated the dose-dependent effects of **HL**^**1**^ (Figure 4C). Similar to paclitaxel and colchicine, MDA-MB-231 cells were treated with increasing concentrations of **HL**^**1**^ corresponding to 1 ×, 5 ×, 10 ×, and 20 × IC_50_. Western blotting experiments revealed a marked decrease of the polymerized tubulin fraction in cells treated with a high concentration of **HL**^**1**^, similar to colchicine. These results indicate that **HL**^**1**^ exhibited a microtubule-destabilizing mechanism of action, in agreement with the initial hypothesis. With respect to IC_50_ values, **HL**^**1**^ demonstrated a microtubule-destabilizing behavior at significantly lower concentrations than did colchicine, which might be beneficial for its further therapeutic development.

### Pilot *In Vivo* Experiments

Inspired by the observed tubulin-targeting effects of indole-modified latonduine derivatives and their complexes *in vitro*, we aimed to study their anticancer effects *in vivo*. To determine the maximum tolerated dose (MTD) of complex **1**, nude athymic mice (6–8 weeks old, *n* = 3 per group) were slowly i.v. injected with increasing concentrations of the compound of interest (1–15 mg/kg). When mice were injected with 10 mg/kg **1** in 100 μL of sterile PEG-300/PBS (4% *v*/*v*), no weight change or visible side effects were observed. However, when the dose of **1** was increased to 15 mg/kg, slight side effects, namely, swollen red eyes and, to a minor extent, uncontrolled convulsions, were observed. On the basis of these observations, the proposed MTD for the i.v. application was 10 mg/kg. To confirm that administration of complex **1** did not cause any abnormalities in kidneys or liver, all groups of animals were humanely sacrificed at day 7, and organ damage was assessed. The whole blood analysis revealed slightly elevated kidney (BUN and CREA) and liver markers (AST/ALT), while other markers were within acceptable range values (Table S2).

**Figure 5:**
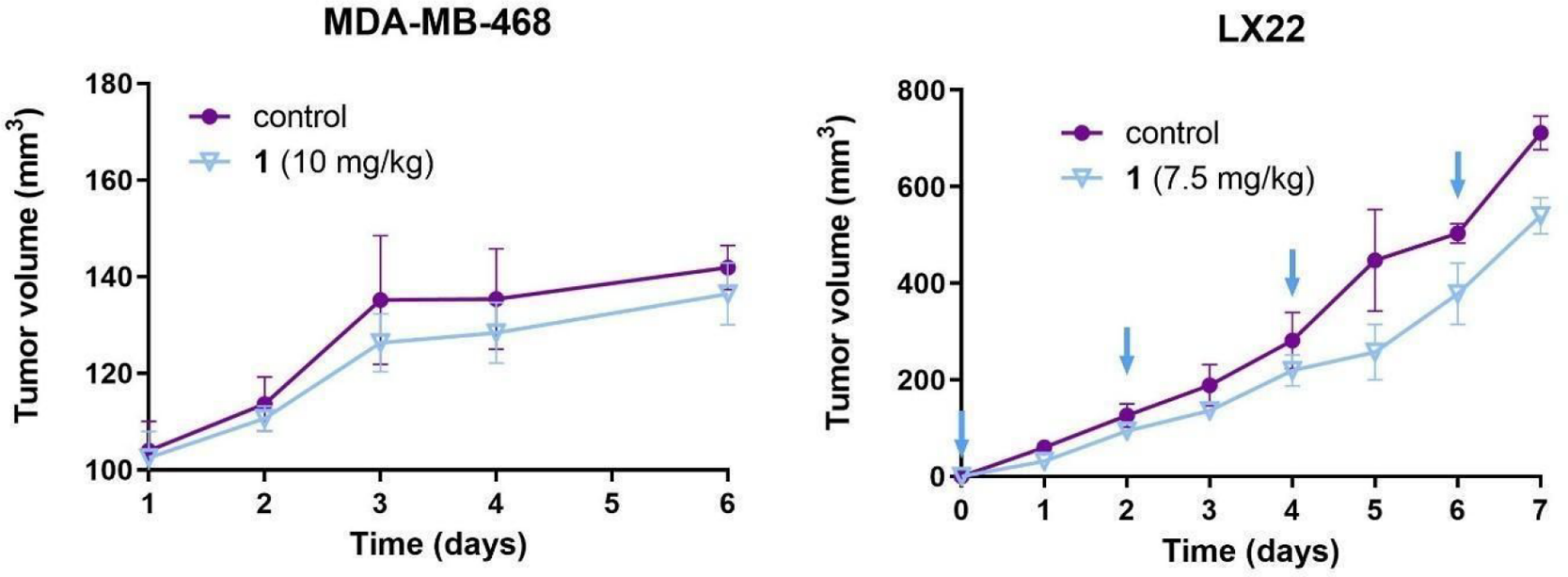
Growth curves of MDA-MB-468 and LX22 tumors starting from the first injection of complex **1**. The days of injections in the LX22 tumor model are indicated with arrows. Curves are means and standard deviations (*n* = 6 (MDA-MB-468) and *n* = 4 (LX22)) of tumor volume presented in mm^3^. Tumor dimensions were measured using a caliper, and tumor volumes were calculated according to the formula: *V* = Width × Length × Depth.

Subsequently, we investigated the effects of compound **1** on tumor growth in two different mouse tumor models, namely, MDA-MB-468 (breast) and LX22 (small cell lung) (Figure 5).^71,72^ Initially, human breast cancer MDA-MB-468 cells were subcutaneously implanted into immunodeficient athymic mice (*n* = 6 per group), and when tumors became palpable (∼100 mm^3^), they were treated with compound **1** as a single injection at day 0 (10 mg/kg, i.v. route) and subsequently monitored for 6 days. Unexpectedly, the majority of the tumors in a control group quickly developed ulcerating tumors; therefore, the mice were humanely euthanized. Even though no changes in tumor growth were observed between the control and treated groups, no mouse in the treated group developed tumor ulceration or necrosis.

Subsequently, we tried a second animal model, using rapidly growing human LX22 lung cancer cells. Cells were subcutaneously implanted into immunodeficient athymic mice (*n* = 4 per group), and mice were injected with either complex **1** (7.5 mg/kg, i.v. route) or the vehicle (4% PEG300/PBS) at days 0, 2, 4, and 6 after tumors reached 100 mm^3^. It was demonstrated that mice treated with complex **1** were characterized by 1.3-fold lower tumor volumes in comparison with those of the control group (540 ± 53 mm^3^ vs 712 ± 42 mm^3^, respectively). Additionally, the whole blood of drug-treated mice was analyzed for the possible side effects (see Table S3). The data showed no abnormalities in the blood chemistry of treated mice, suggesting no hematological toxicity induced by the repeated treatment of **1**.

## Conclusions

Microtubule-targeting drugs, such as paclitaxel, docetaxel, vinblastine, and others, are in clinical use for the treatment of cancers, for which cisplatin is not used. However, their use has several drawbacks, such as acquired resistance and severe side effects. In this project, we developed novel tubulin-targeting compounds using the scarcely discussed indolo[2,3-*d*]benzazepine scaffold. We demonstrated that two novel indololatonduines were highly cytotoxic, while their metal complexes were significantly less active. Both indololatonduines and their metal complexes demonstrated tubulin-targeting effects. In particular, indololatonduine **HL**^**1**^ showed excellent microtubule-destabilizing activity and was even more potent and less cytotoxic than colchicine. However, Ru^II^- and Os^II^-*p*-cymene complexes **1**–**4** revealed decreased activity, maybe due to loss of planarity of the molecules. Pilot *in vivo* studies revealed that complex **1** was well-tolerated in mice; however, the tumor growth was not significantly inhibited. These studies provide the basis for further optimization of the indololatonduine scaffold and its transition metal complexes for future development of microtubule-destabilizing drugs.

## Supporting Information Available

NMR numbering scheme, NMR spectra, ESI-MS data, additional X-ray crystallographic data, time-dependent ^1^H NMR spectra and UV–vis fluorescence data, HPLC-MS report, concentration effect curves, blood chemistry analysis of mice treated with complex **1** (PDF)

## Accession Codes

CCDC 2109919–2109921 contain the supplementary crystallographic data for this paper. These data can be obtained free of charge via www.ccdc.cam.ac.uk/data_request/cif, or by emailing data_request@ccdc.cam.ac.uk, or by contacting The Cambridge Crystallographic Data Centre, 12 Union Road, Cambridge CB2 1EZ, UK; fax: +44 1223 336033.
