# Supplementary material for: Inhibition of Microtubule Dynamics in Cancer Cells by Indole-Modified Latonduine Derivatives and Their Metal Complexes
Source: Inorg Chem. 2022 Jan 7;61(3):1456–70. doi: 10.1021/acs.inorgchem.1c03154 (PMC8790753; doi:10.1021/acs.inorgchem.1c03154)
Supplement: Supplementary file 1 — ic1c03154_si_001.pdf [file ic1c03154_si_001.pdf]

Supporting Information
for
Inhibition of microtubule dynamics in cancer cells by indole-modified latonduine derivatives and their metal complexes

Christopher Wittmann,[†] Anastasiia S. Sivchenko,[‡] Felix Bacher,[†] Kelvin K. H. Tong,[‡] Navjot Guru,[∆] Thomas Wilson,[∆] Junior Gonzales,[∆] Hartmut Rauch,[≠] Susanne Kossatz,[≠,⊥] Thomas Reiner[∆,◊,§,*], Maria V. Babak,[‡,*] Vladimir B. Arion[†,*]

[†]University of Vienna, Institute of Inorganic Chemistry, Währinger Strasse 42, A-1090 Vienna, Austria

[‡]Drug Discovery Lab, Department of Chemistry, City University of Hong Kong, 83 Tat Chee Avenue, Hong Kong SAR, 999077, People's Republic of China

[∆]Department of Radiology, Memorial Sloan Kettering Cancer Center, 417 E 68th Street, New York, NY, 10065, USA

[◊]Department of Radiology, Weill Cornell Medical College, New York, NY, USA

[§]Chemical Biology Program, Memorial Sloan Kettering Cancer Center, New York, NY, USA

[≠]Department of Nuclear Medicine, University Hospital Klinikum Rechts der Isar, Technical University Munich, Munich, Germany; TranslaTUM - Central Institute for Translational Cancer Research, D-81675 Munich, Germany

[⊥]Department of Chemistry, Technical University of Munich, D-85748 Munich, Germany

* corresponding authors

Email: vladimir.arion@univie.ac.at

Email: mbabak@cityu.edu.hk

Email: reinert@mskcc.org

Contents

NMR numbering scheme S3
NMR spectra S4
ESI-MS Data S23
Additional X-ray crystallographic data S35
Time-dependent ^{1}H NMR spectra and UV–vis fluorescence data S36
High Performance Liquid Chromatography-MS report of **1** S44
Concentration-effect curves S45
Analysis reports on mice treated with complex **1** S47

NMR numbering scheme

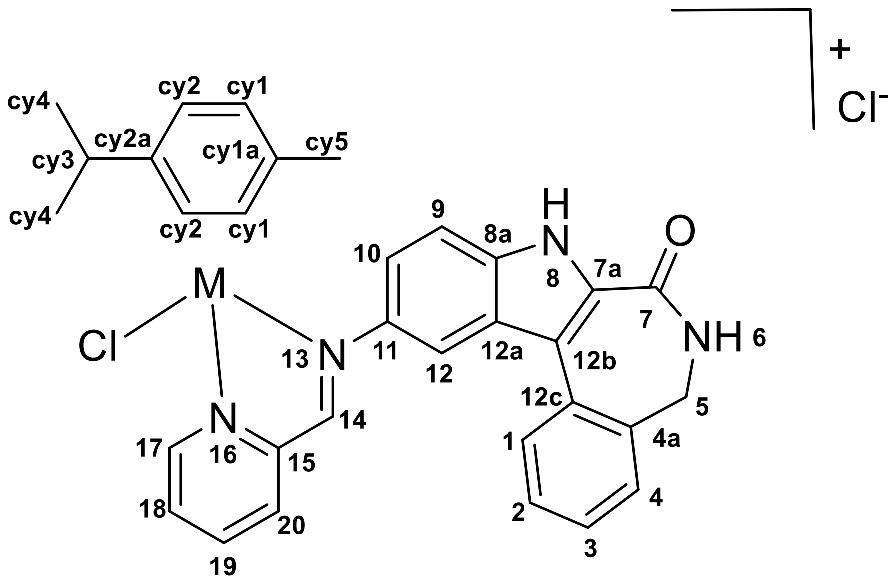

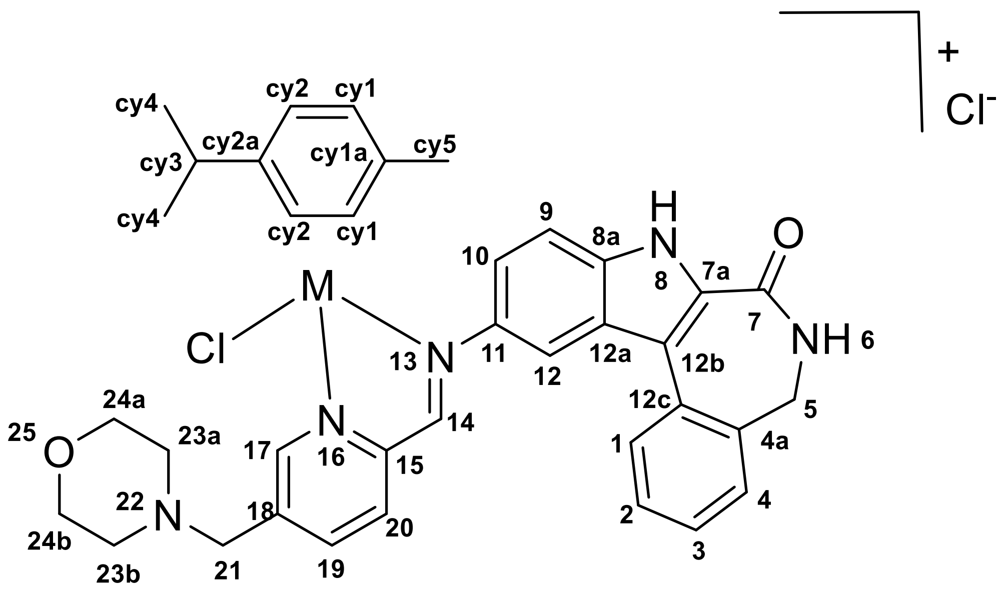

Chart S1. Numbering schemes for the novel metal-arene complexes with M = Ru^{II}, Os^{II}: (top) complexes **1** and **2**; (bottom) complexes **3** and **4**.

NMR spectra

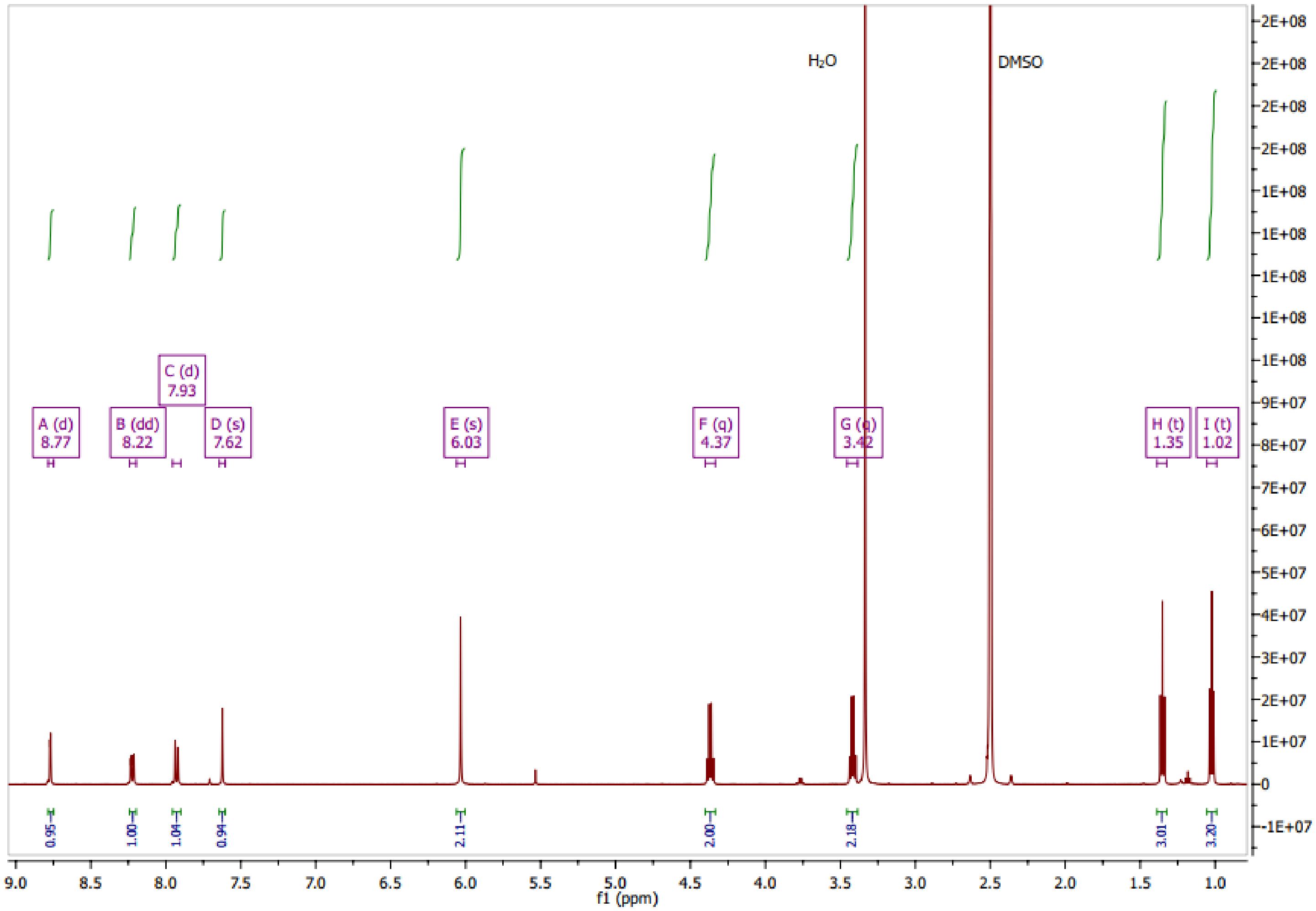

Figure S1. ^{1}H NMR spectrum of ethyl 5-nitro-1-(ethoxymethyl)-1*H*-indole-2-carboxylate (**J**).

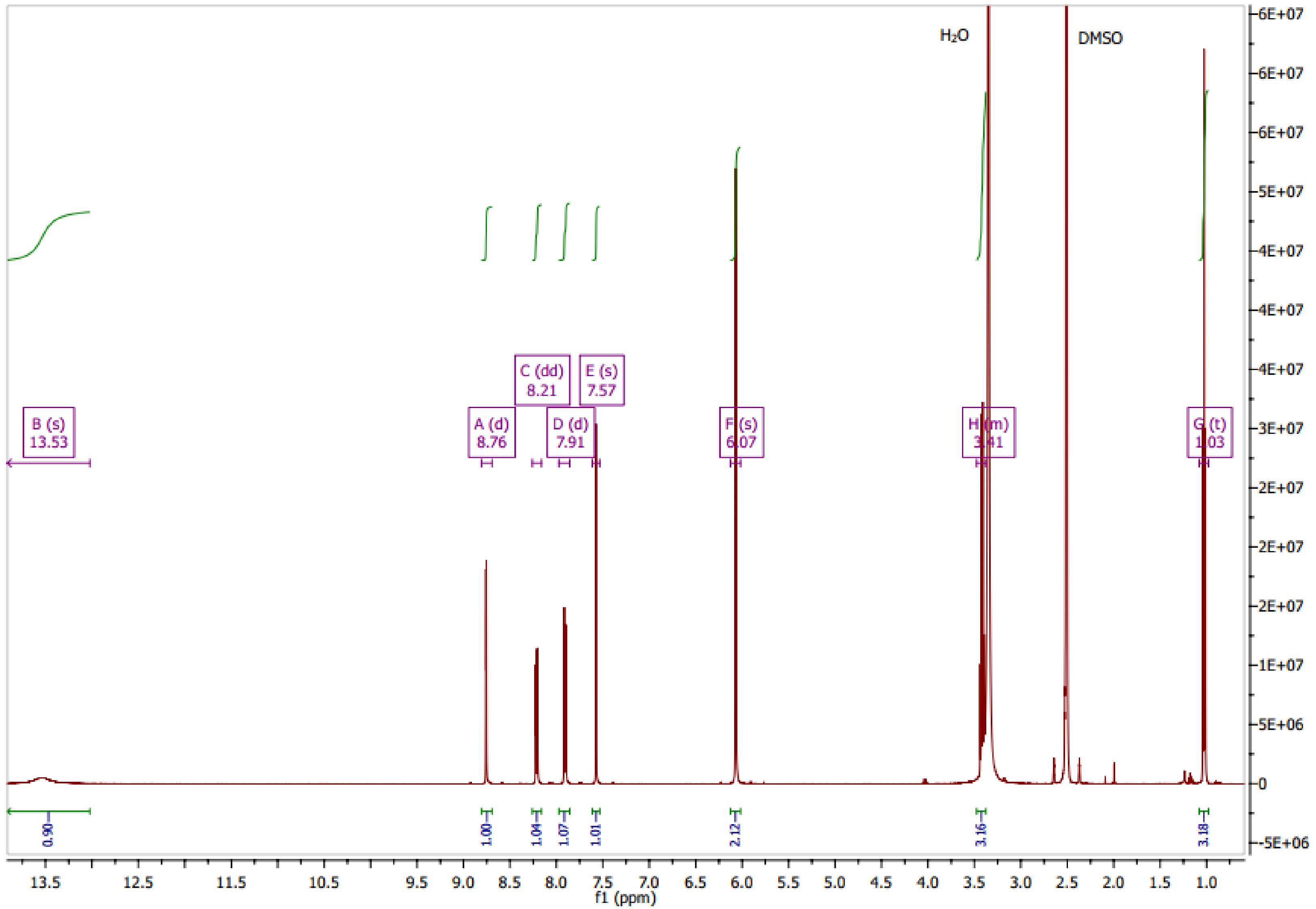

Figure S2. ^{1}H NMR spectrum of 5-nitro-1-(ethoxymethyl)-1*H*-indole-2-carboxylic acid (**K**).

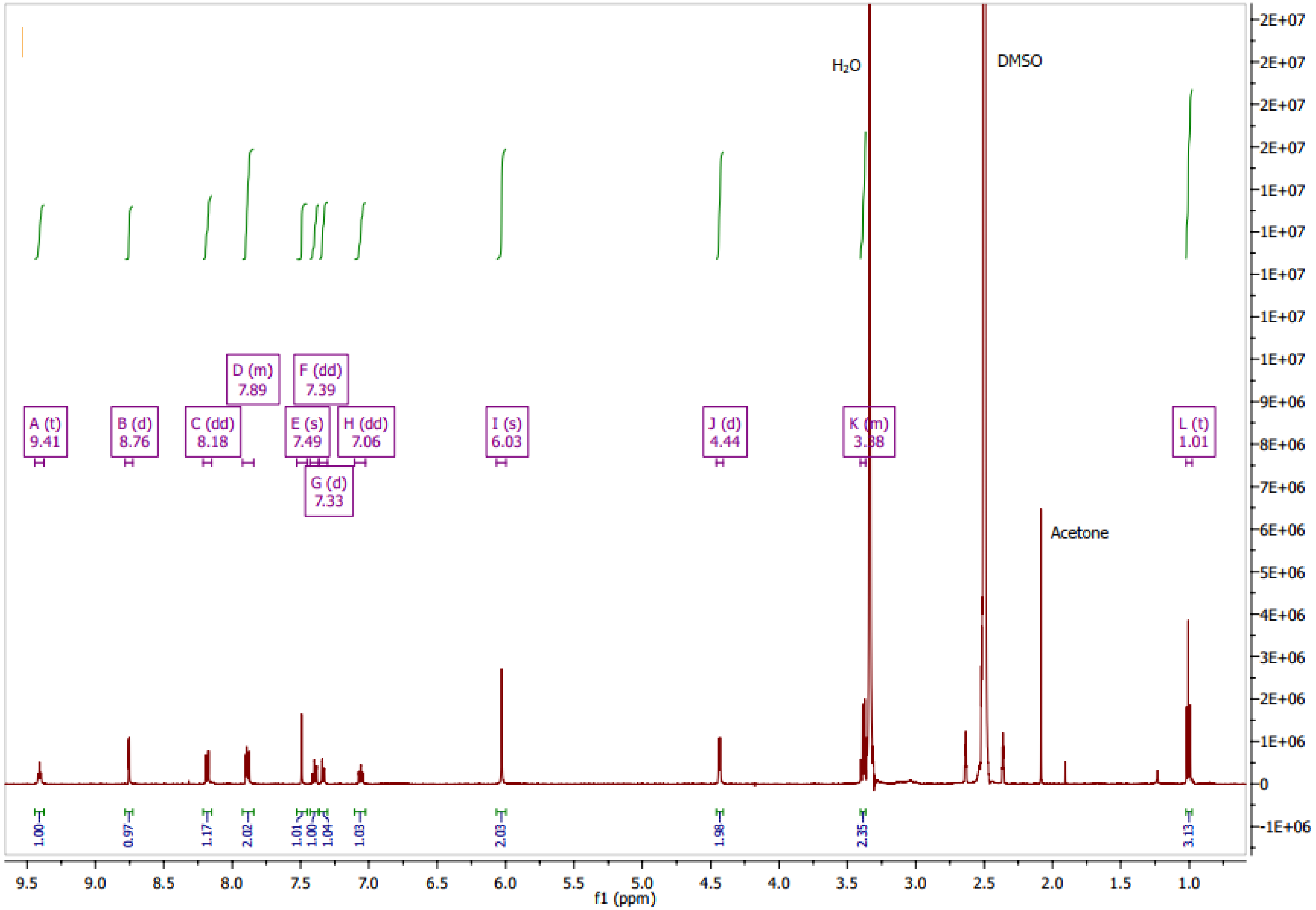

Figure S3. ^{1}H NMR spectrum of 5-nitro-1-(ethoxymethyl)-N-(2-iodobenzyl)-1*H*-indole-2-carboxamide (**L**).

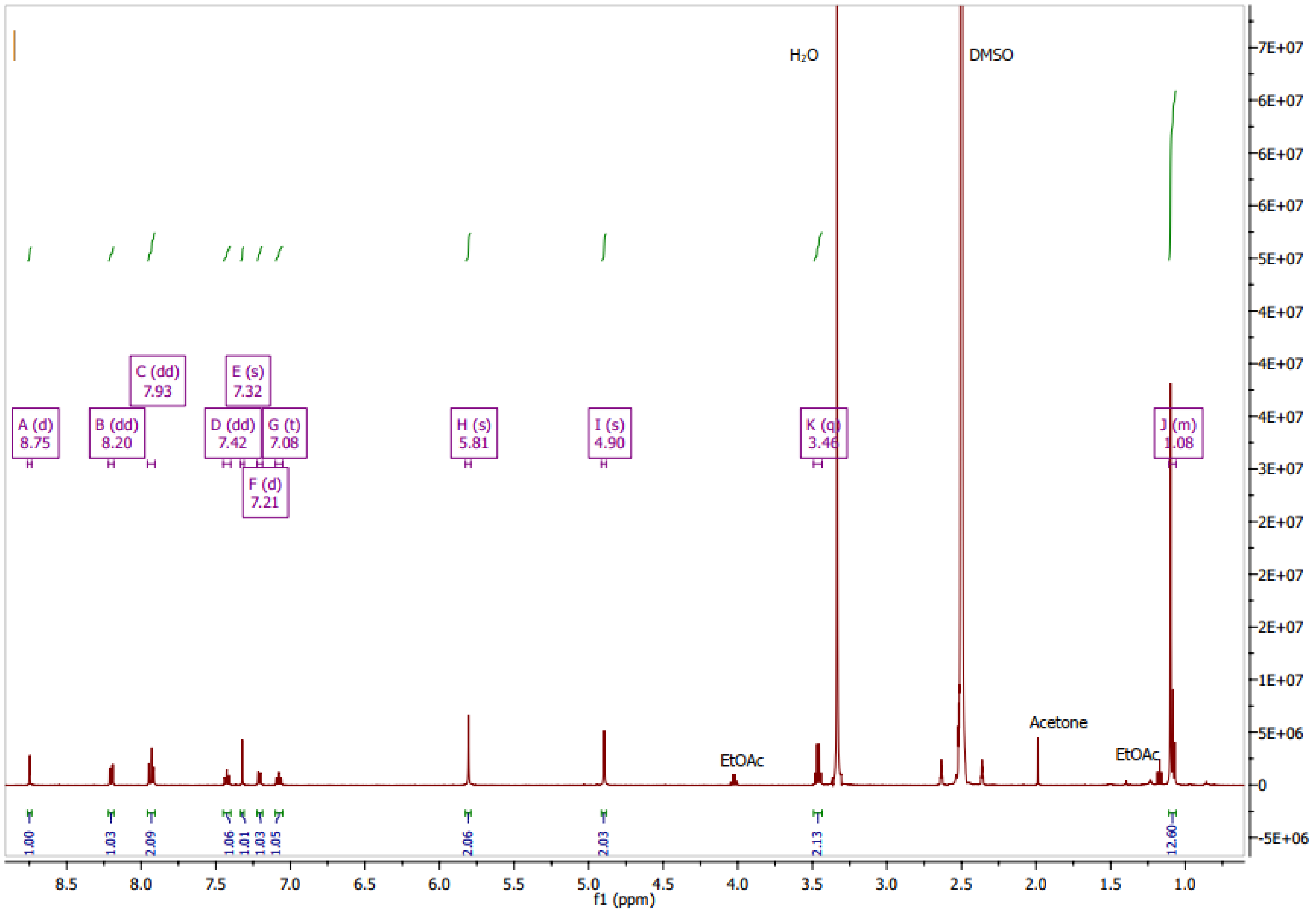

Figure S4. ^{1}H NMR spectrum of *tert*-butyl (5-nitro-1-(ethoxymethyl)-1*H*-indole-2-carbonyl)(2-iodobenzyl)carbamate (**M**).

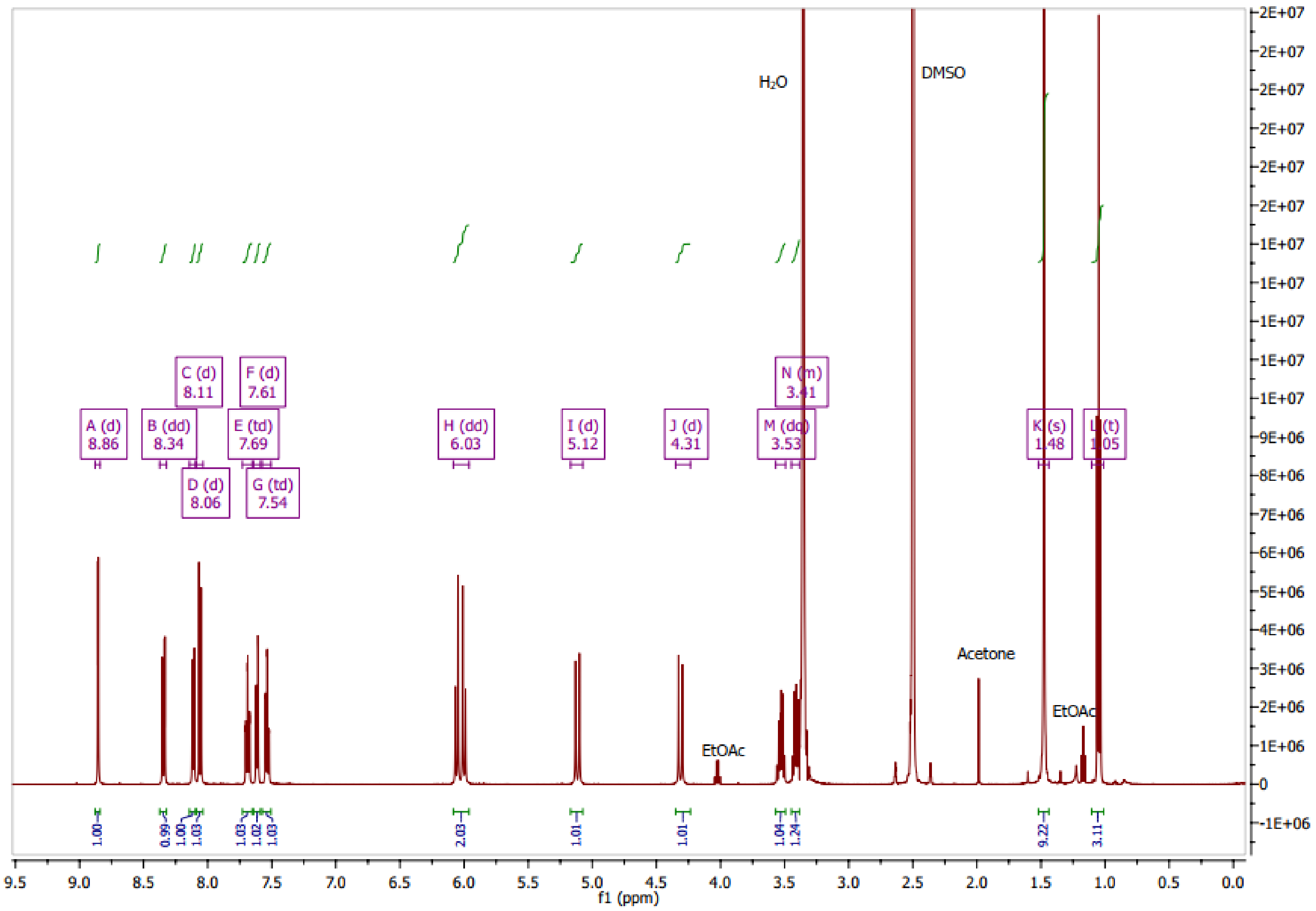

Figure S5. ^{1}H NMR spectrum of *tert*-butyl 11-nitro-8-(ethoxymethyl)-dihydroindolo[2,3-*d*]benzazepin-7-one (**N**).

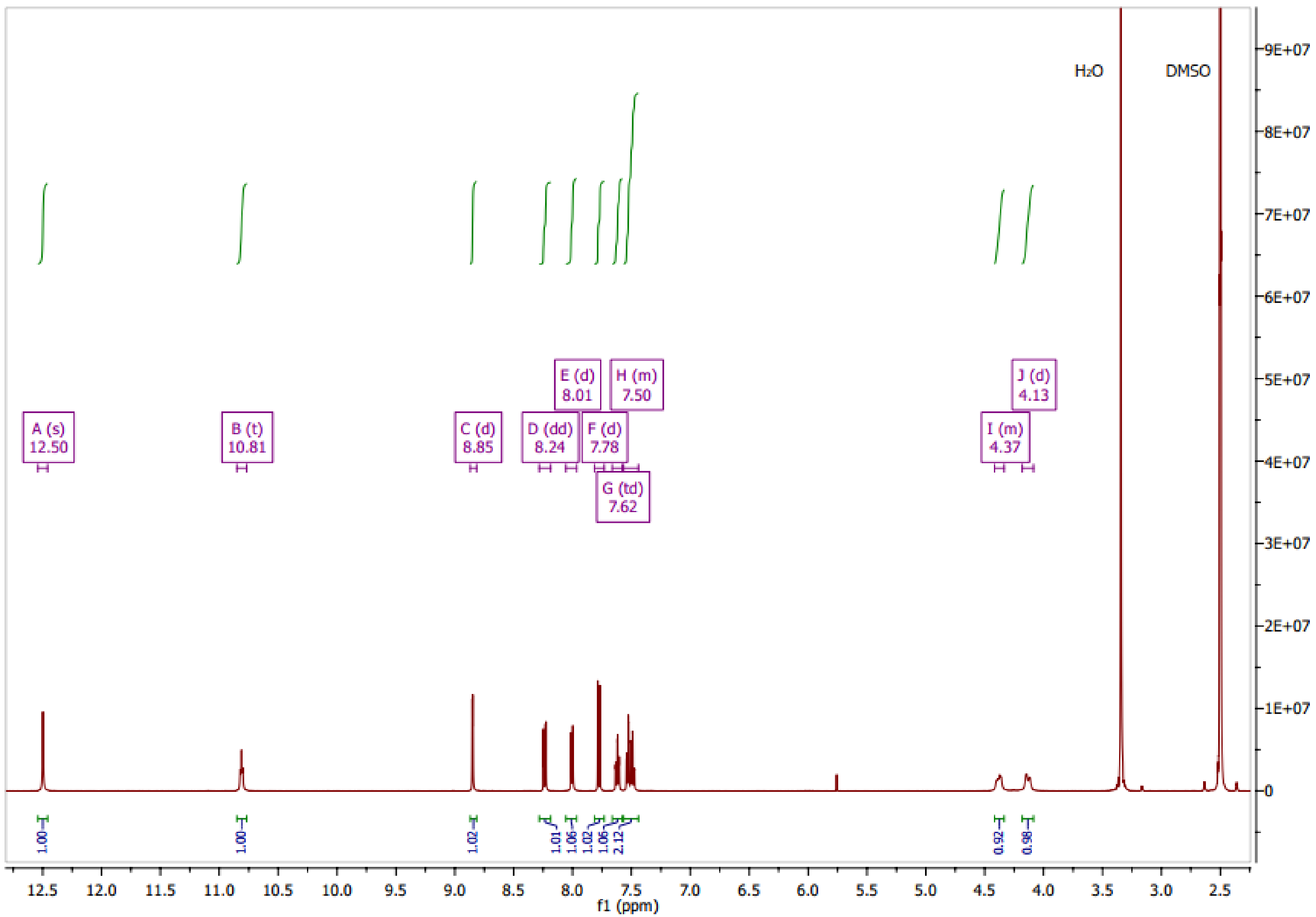

Figure S6. ^{1}H NMR spectrum of 11-nitro-5,8-dihydroindolo[2,3-*d*]benzazepin-7(6*H*)-one (**O**).

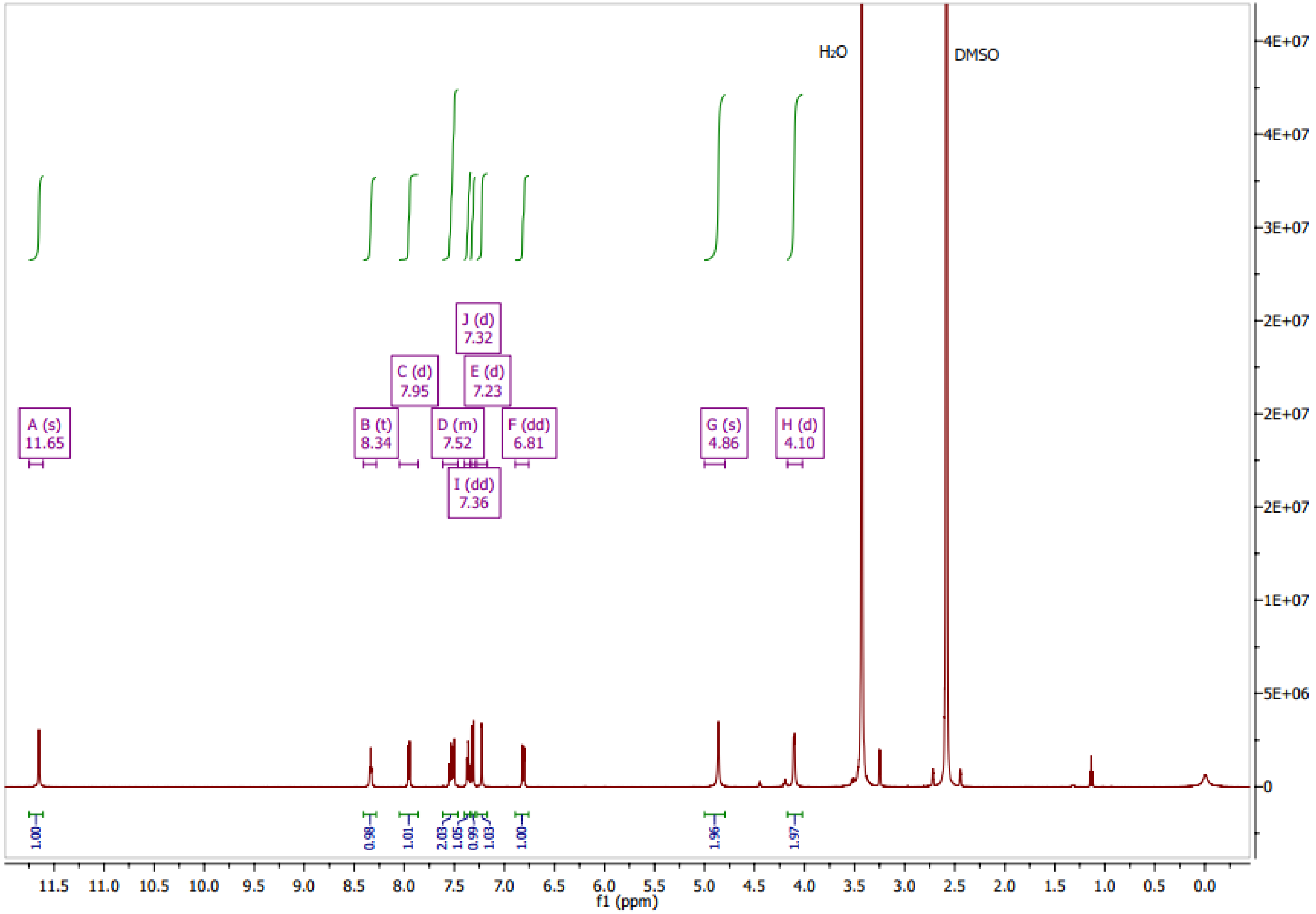

Figure S7. ^{1}H NMR spectrum of 11-amino-5,8-dihydroindolo[2,3-*d*]benzazepin-7(6*H*)-one (**P**).

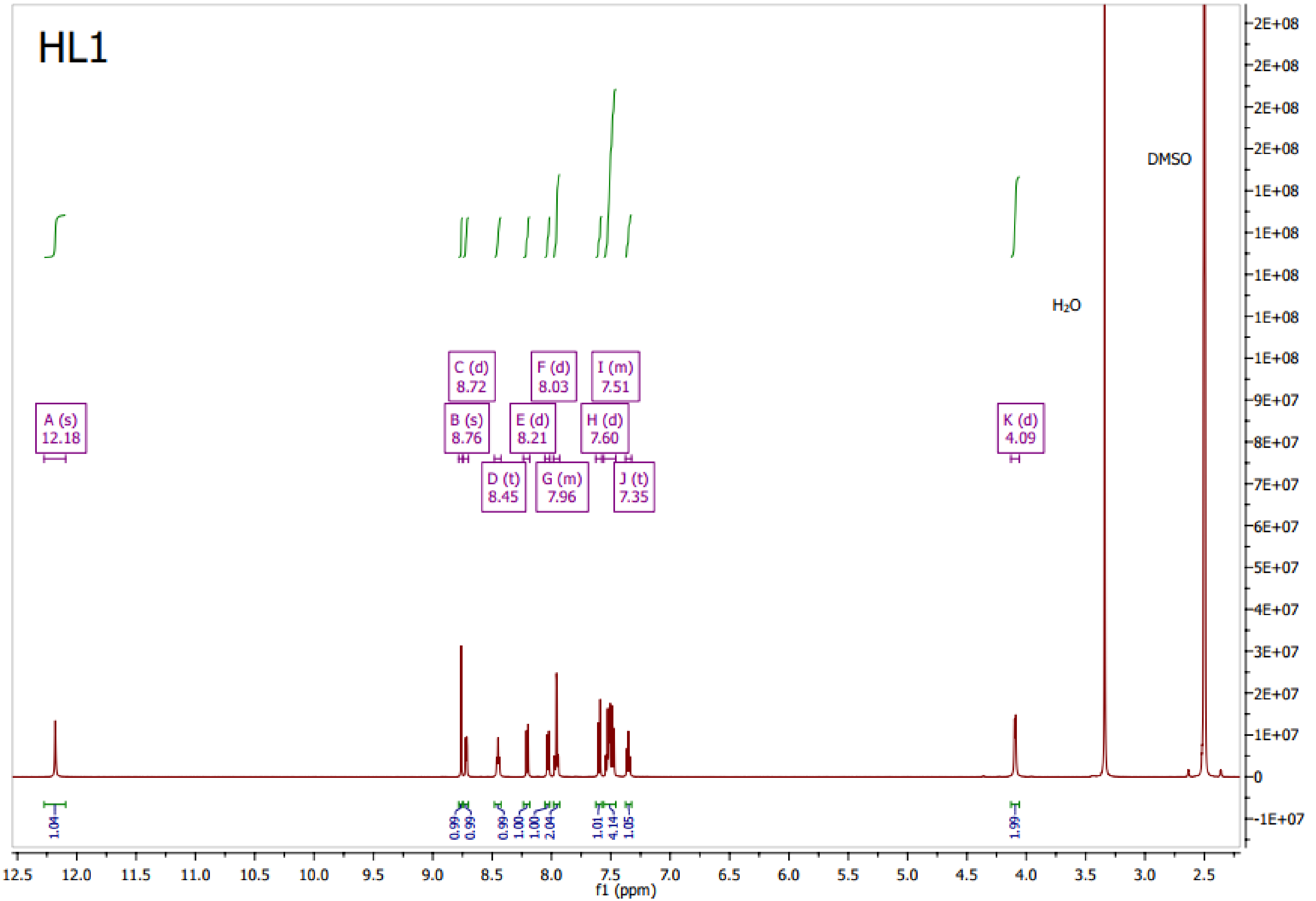

Figure S8. ^{1}H NMR spectrum of **HL¹**.

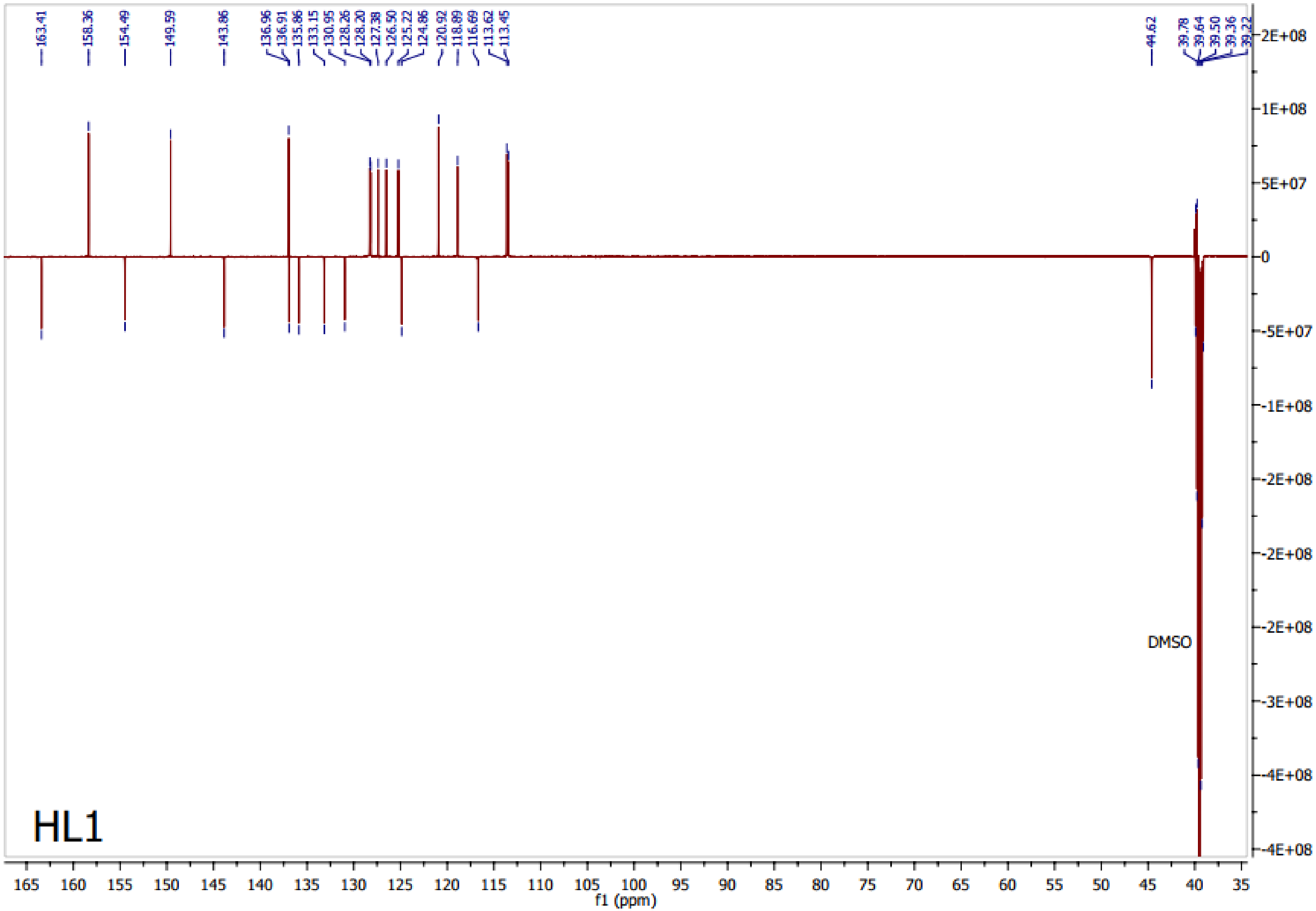

Figure S9. ^{13}C NMR spectrum of **HL[1]**.

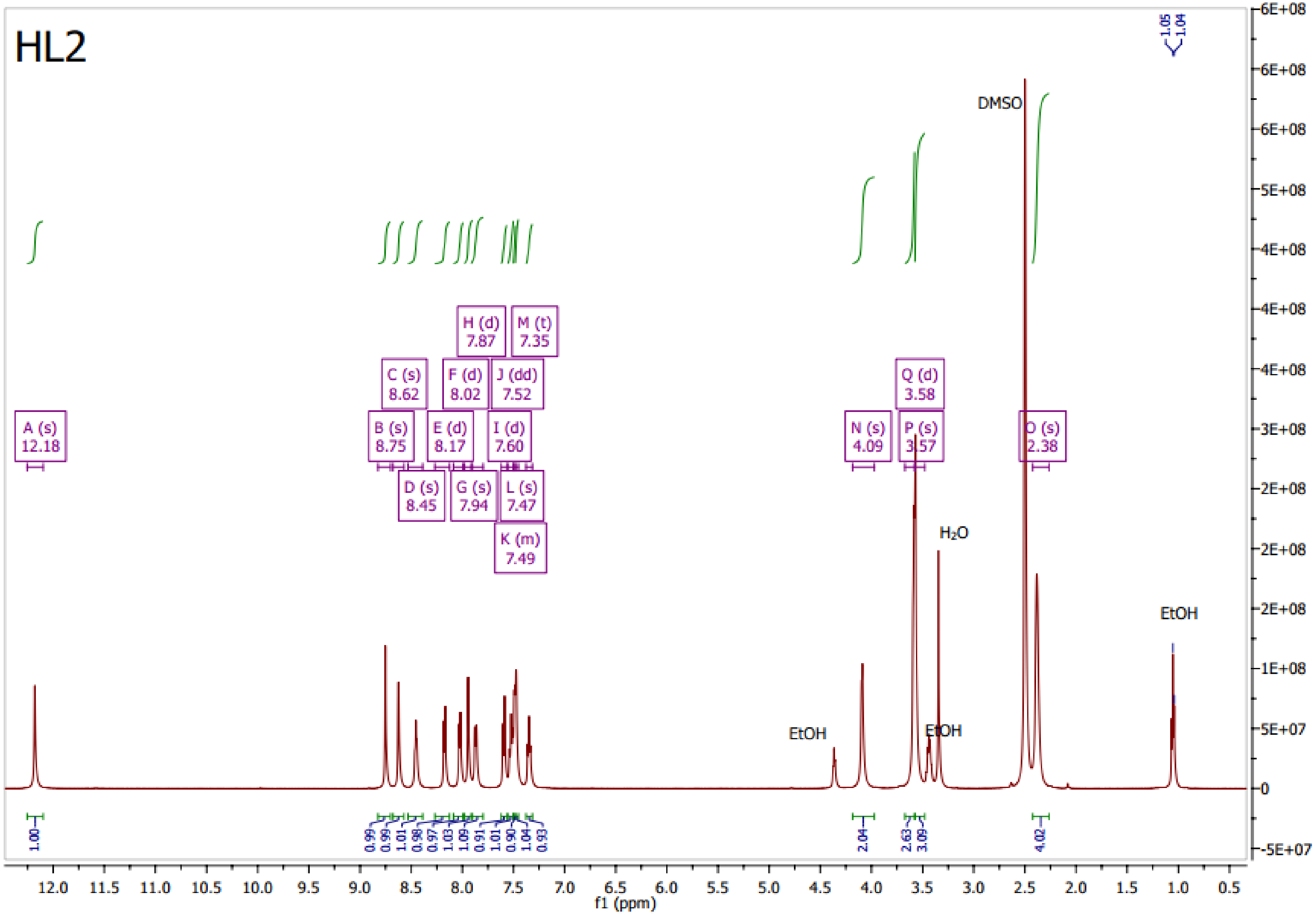

Figure S10. ^{1}H NMR spectrum of **HL²**.

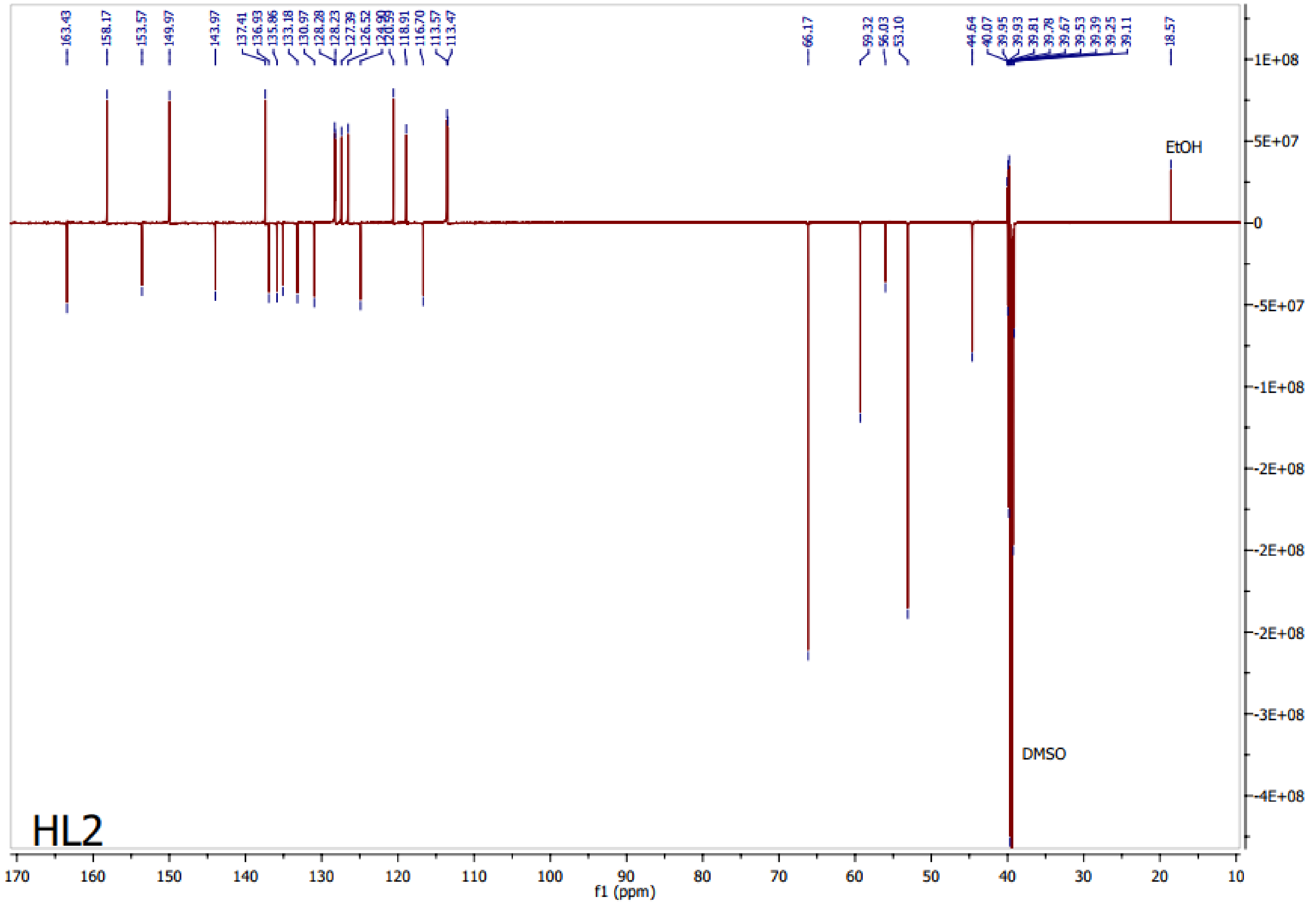

Figure S11. ^{13}C NMR spectrum of **HL^2**.

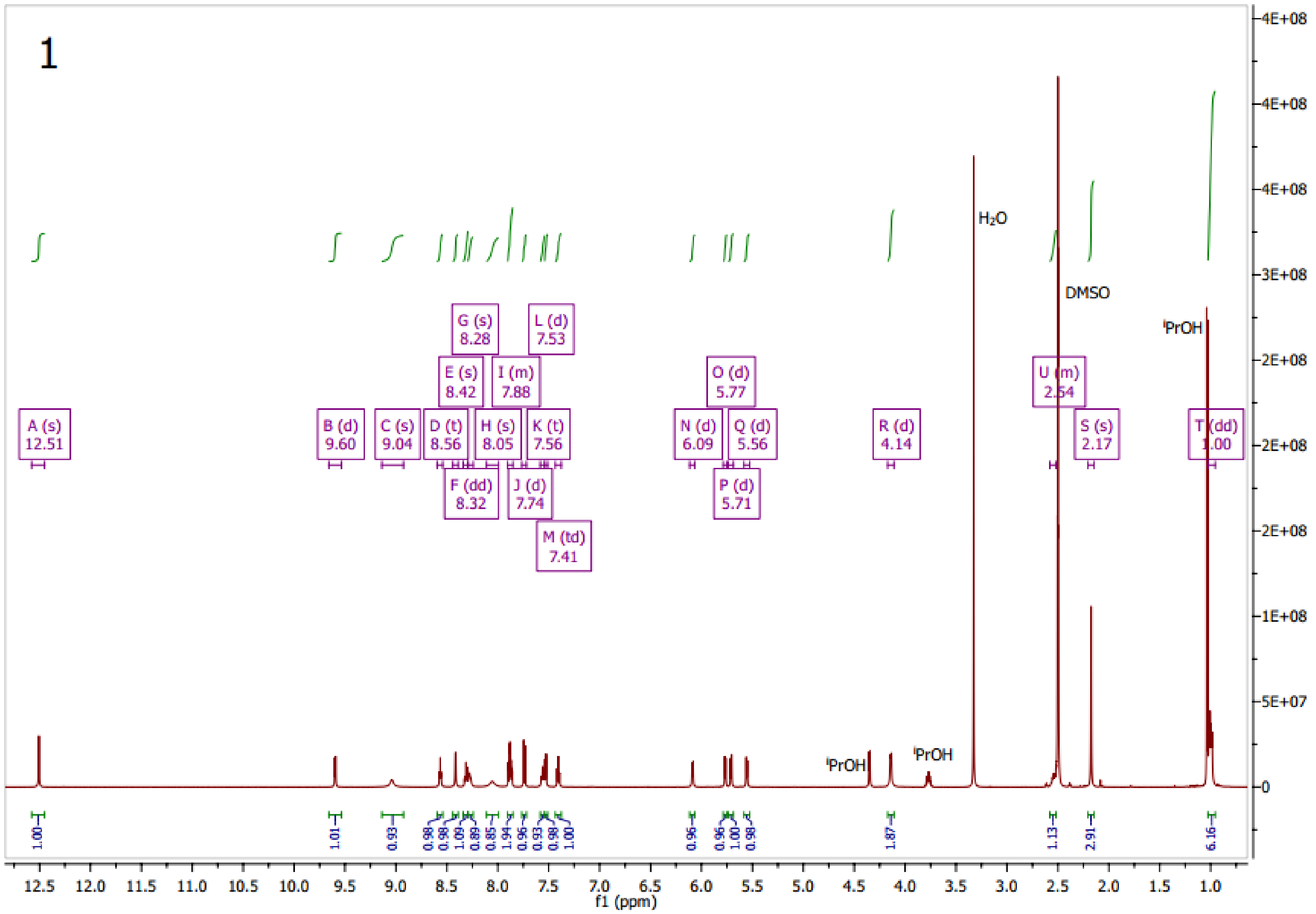

Figure S12. ^{1}H NMR spectrum of **1**.

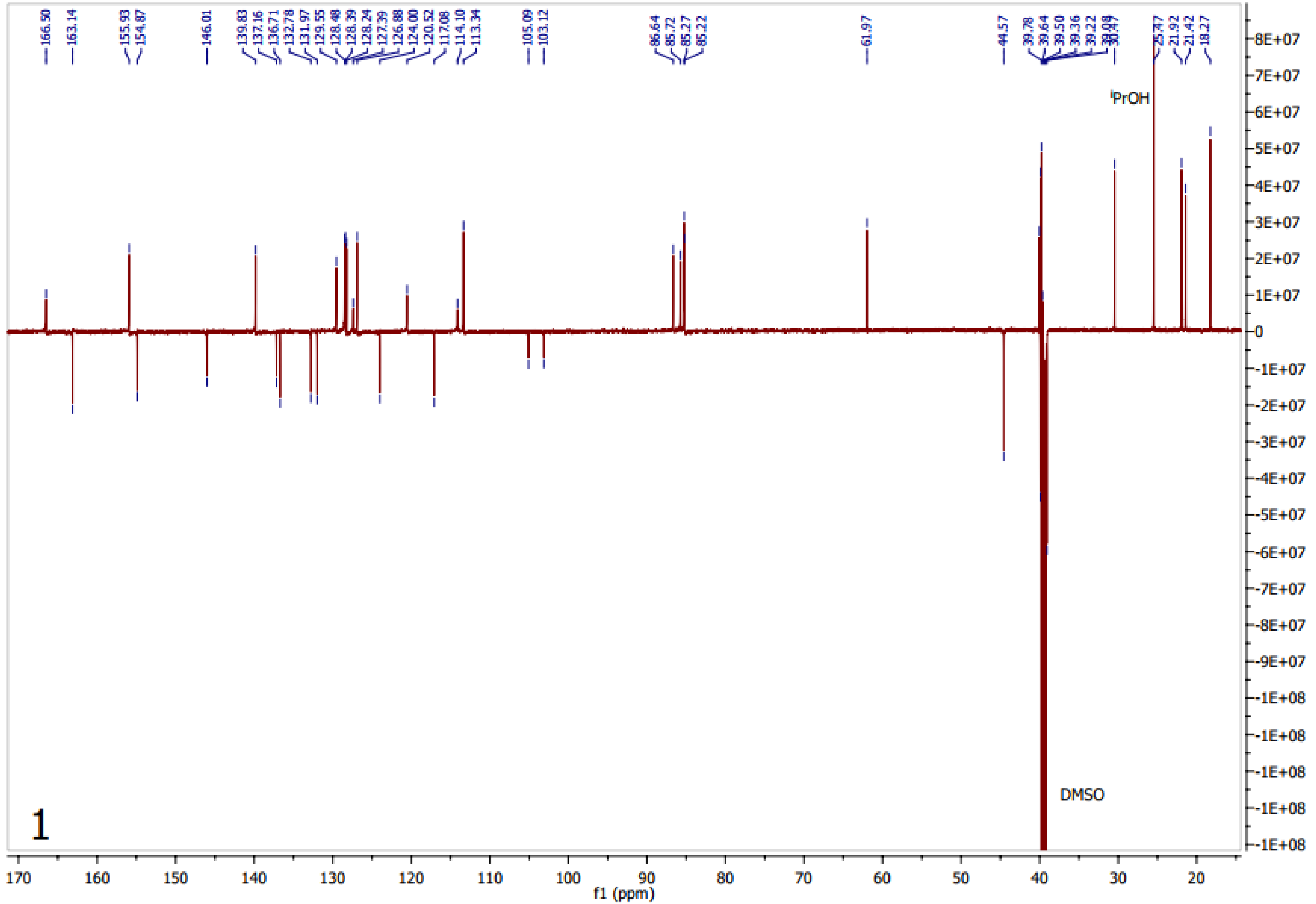

Figure S13. ^{13}C NMR spectrum of **1**.

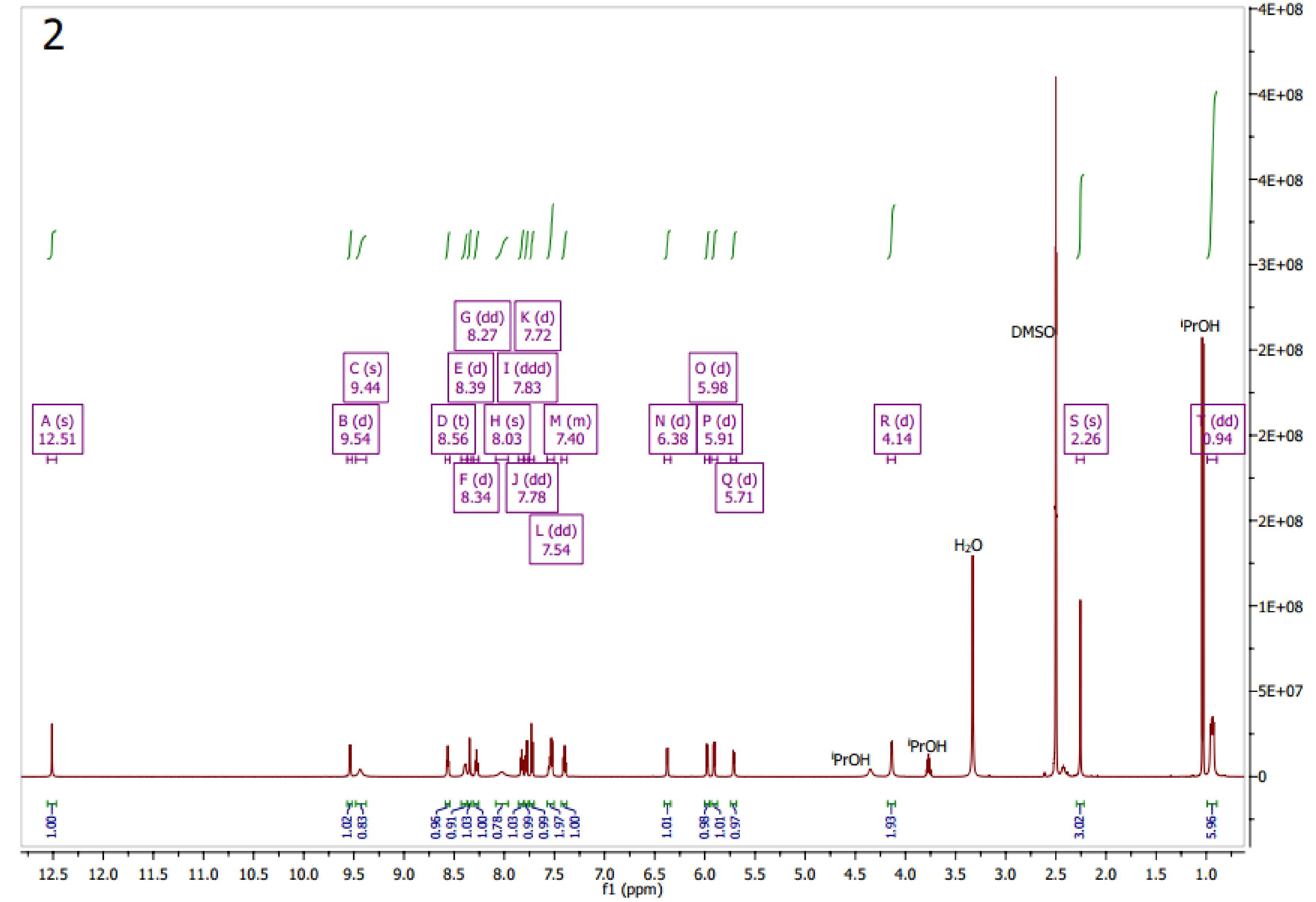

Figure S14. ^{1}H NMR spectrum of **2**.

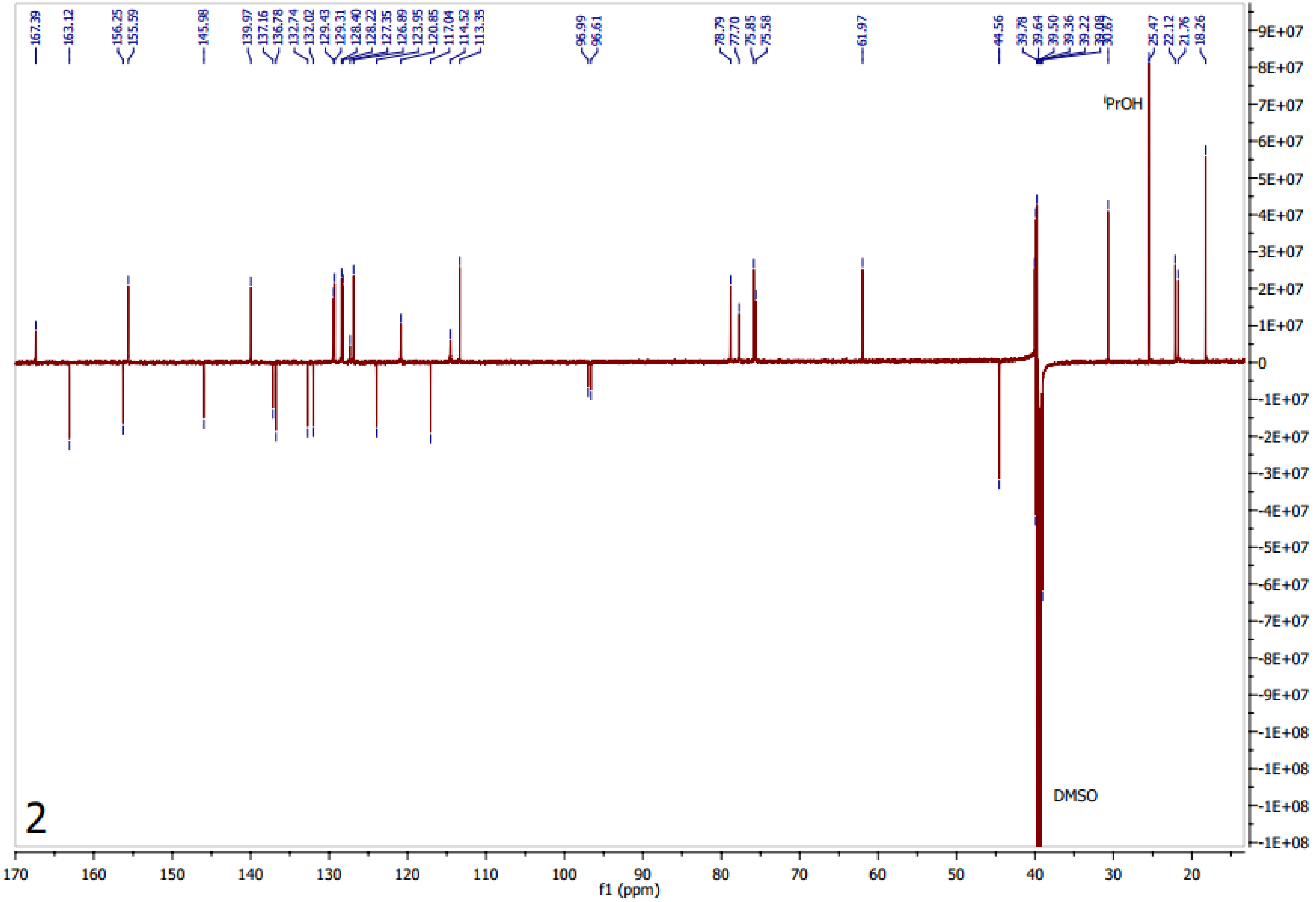

Figure S15. ^{13}C NMR spectrum of **2**.

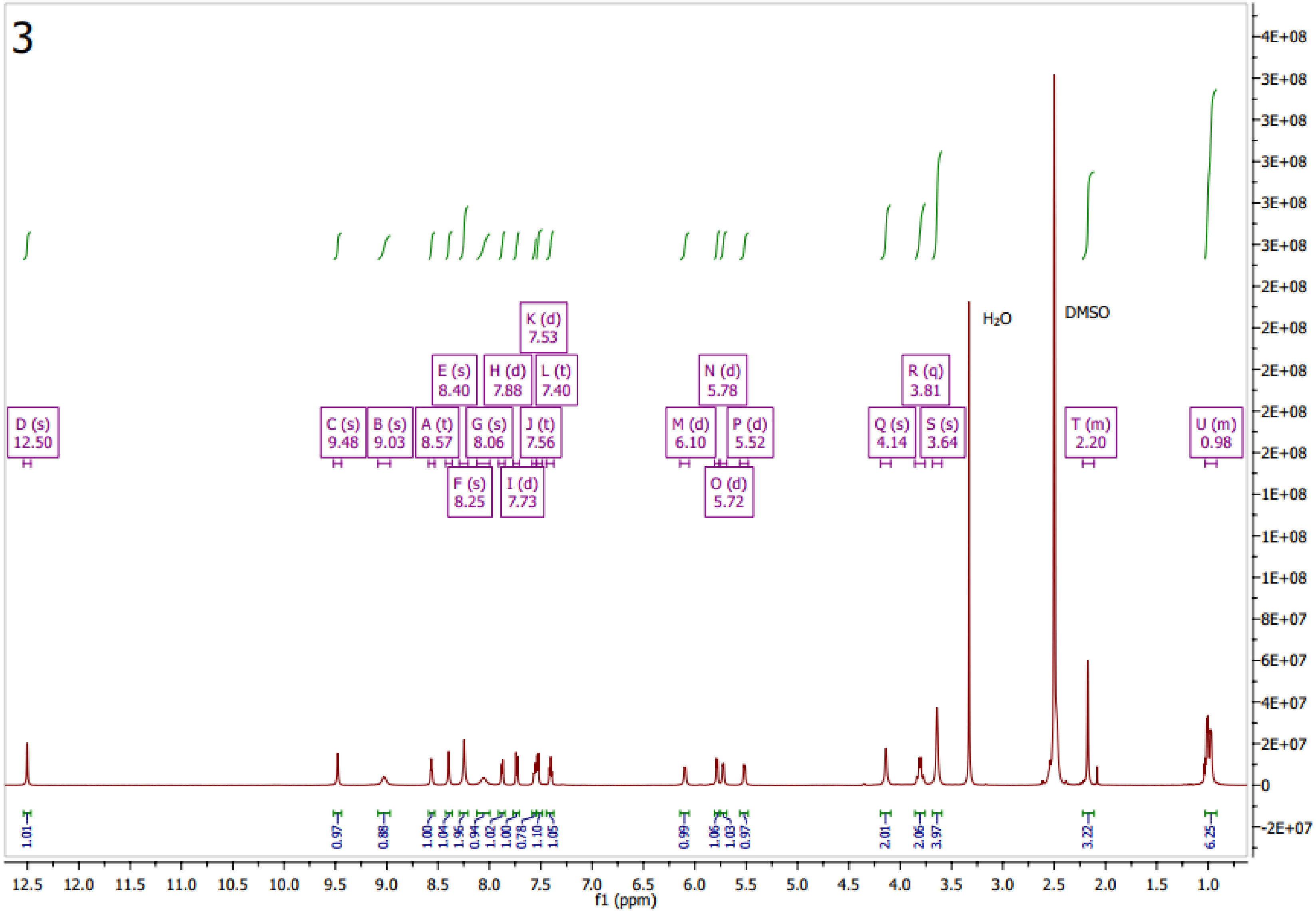

Figure S16. ^{1}H NMR spectrum of **3**.

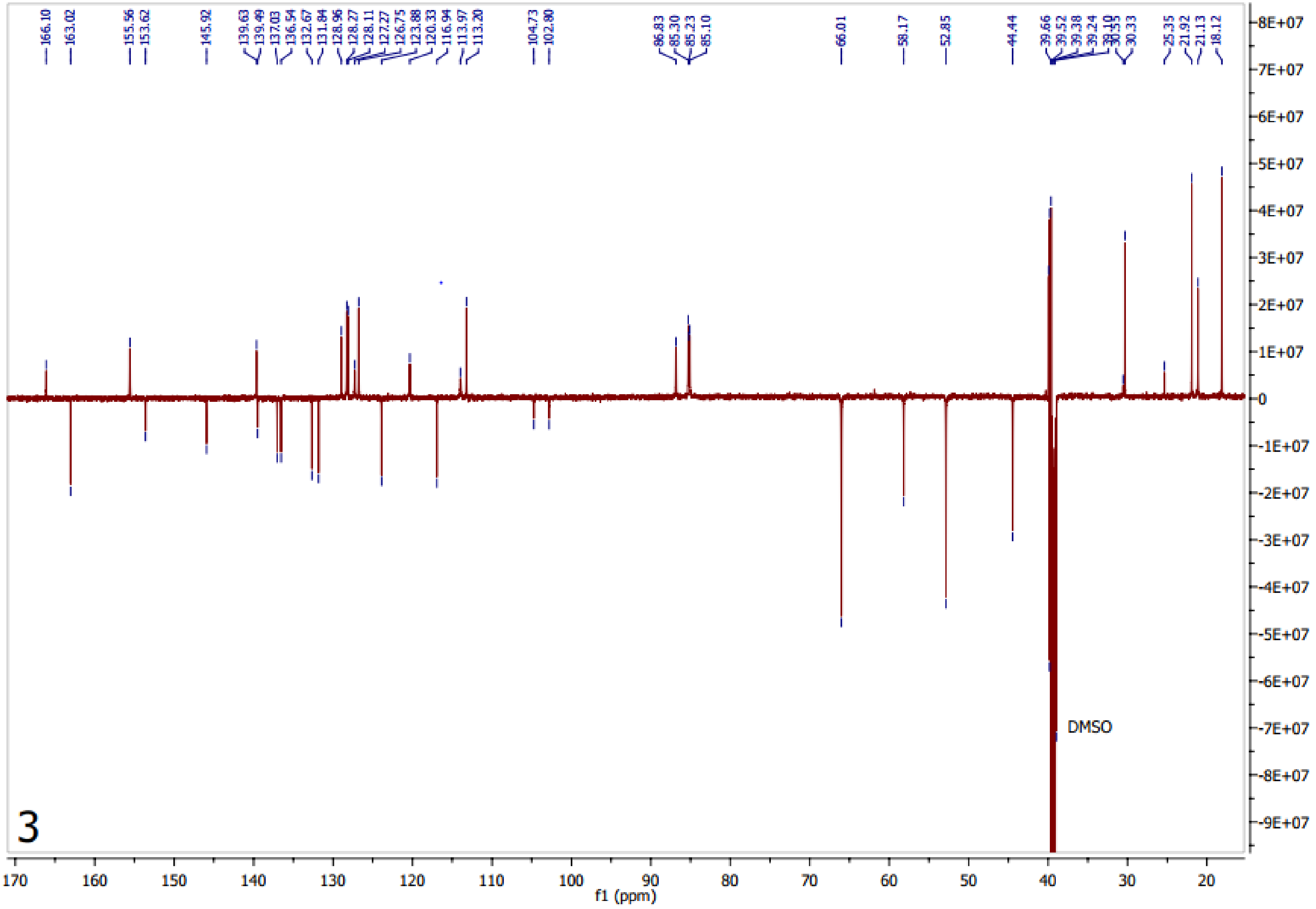

Figure S17. ^{13}C NMR spectrum of **3**.

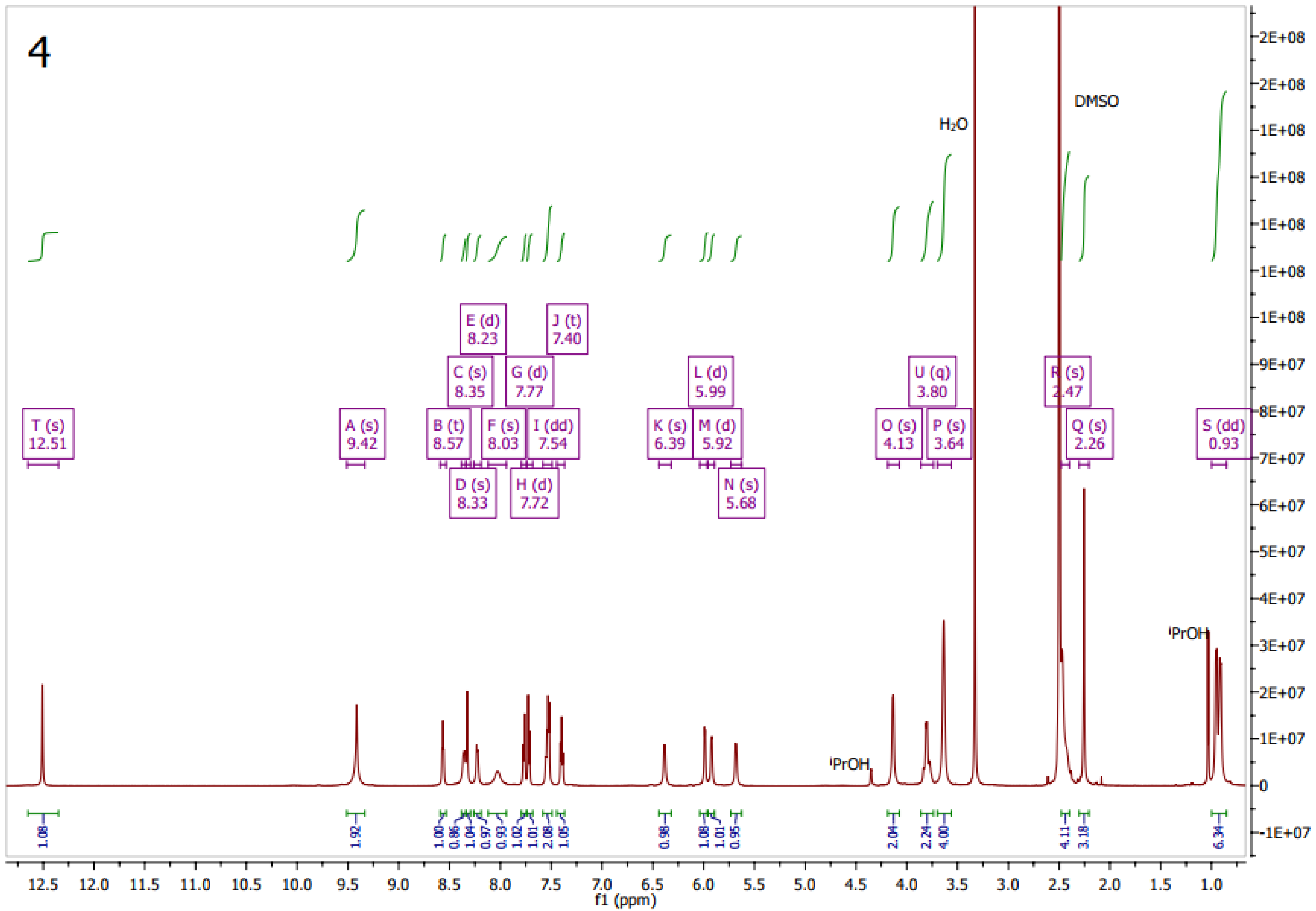

Figure S18. ^{1}H NMR spectrum of **4**.

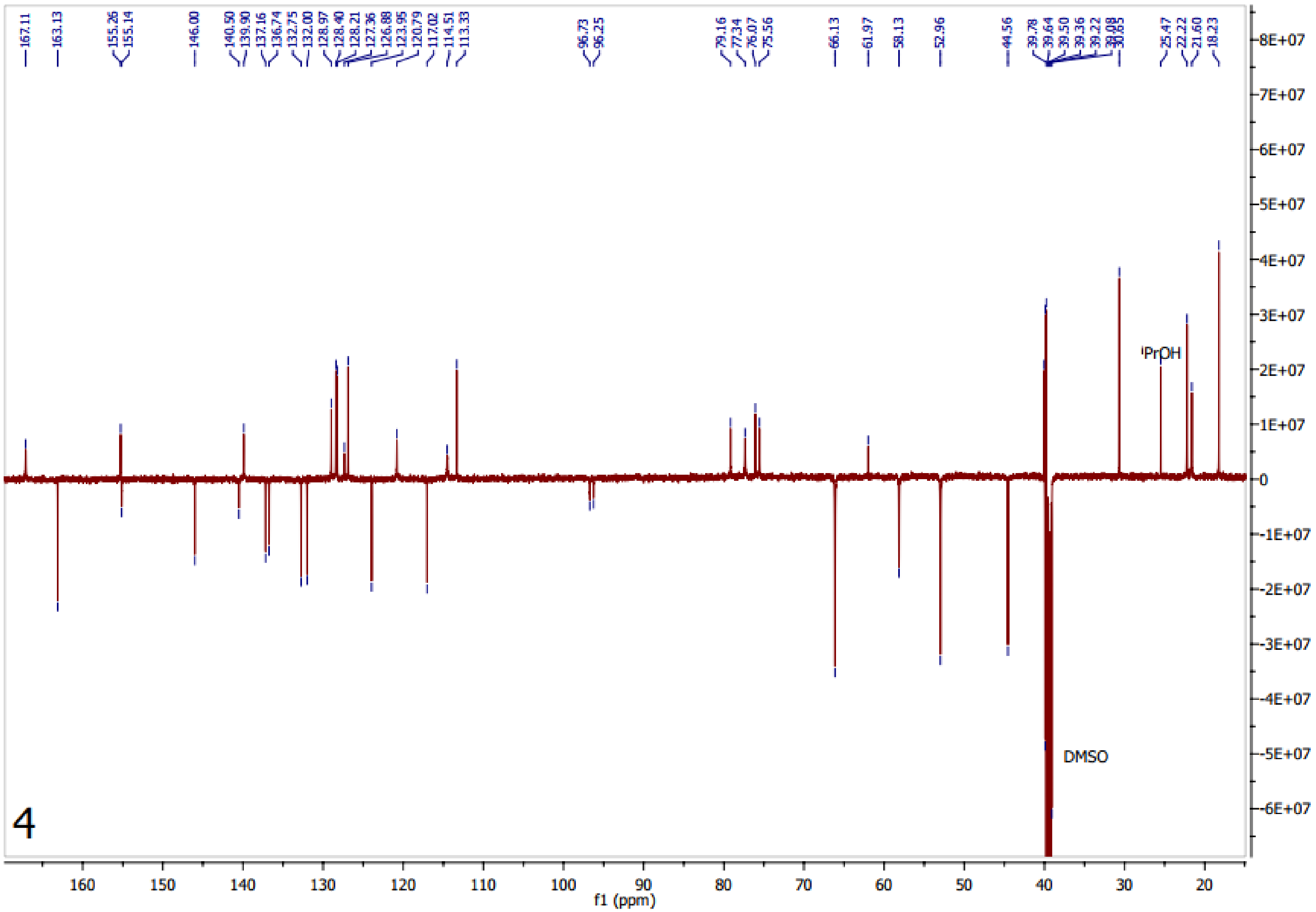

Figure S19. ^{13}C NMR spectrum of **4**

ESI-MS Data

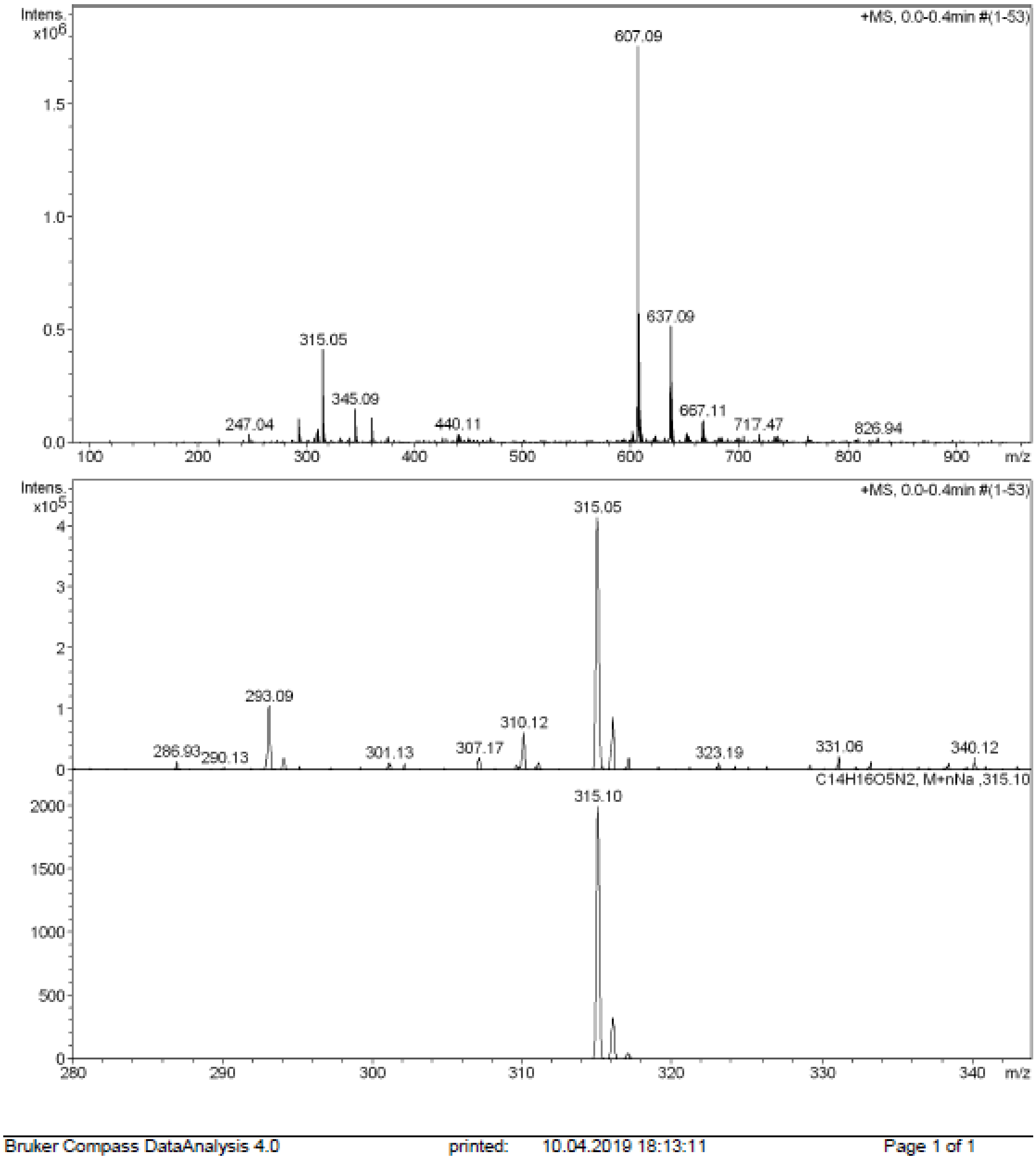

Figure S20. ESI mass spectrum of ethyl 5-nitro-1-(ethoxymethyl)-1*H*-indole-2-carboxylate (**J**).

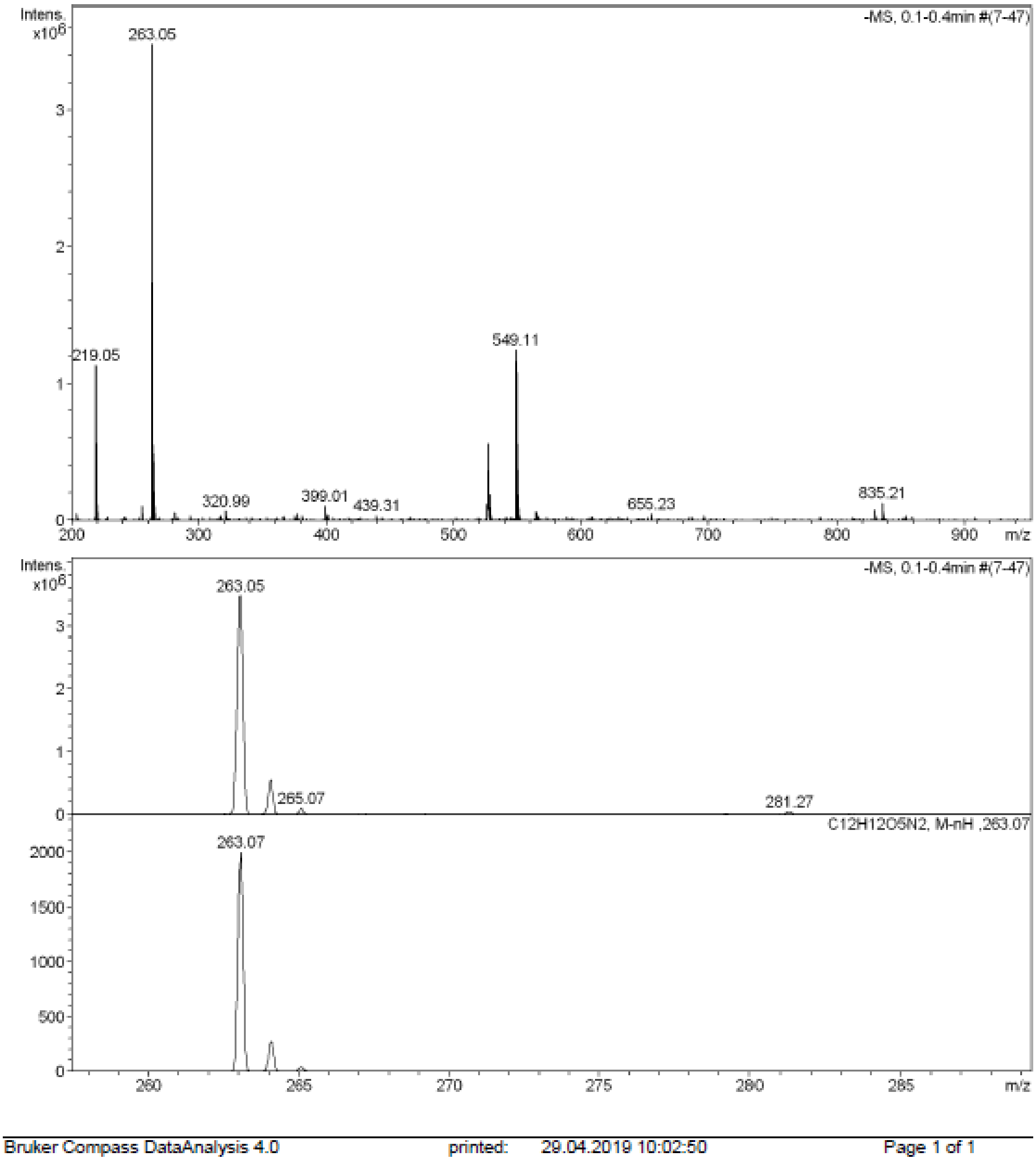

Figure S21. ESI mass spectrum of 5-nitro-1-(ethoxymethyl)-1*H*-indole-2-carboxylic acid (**K**).

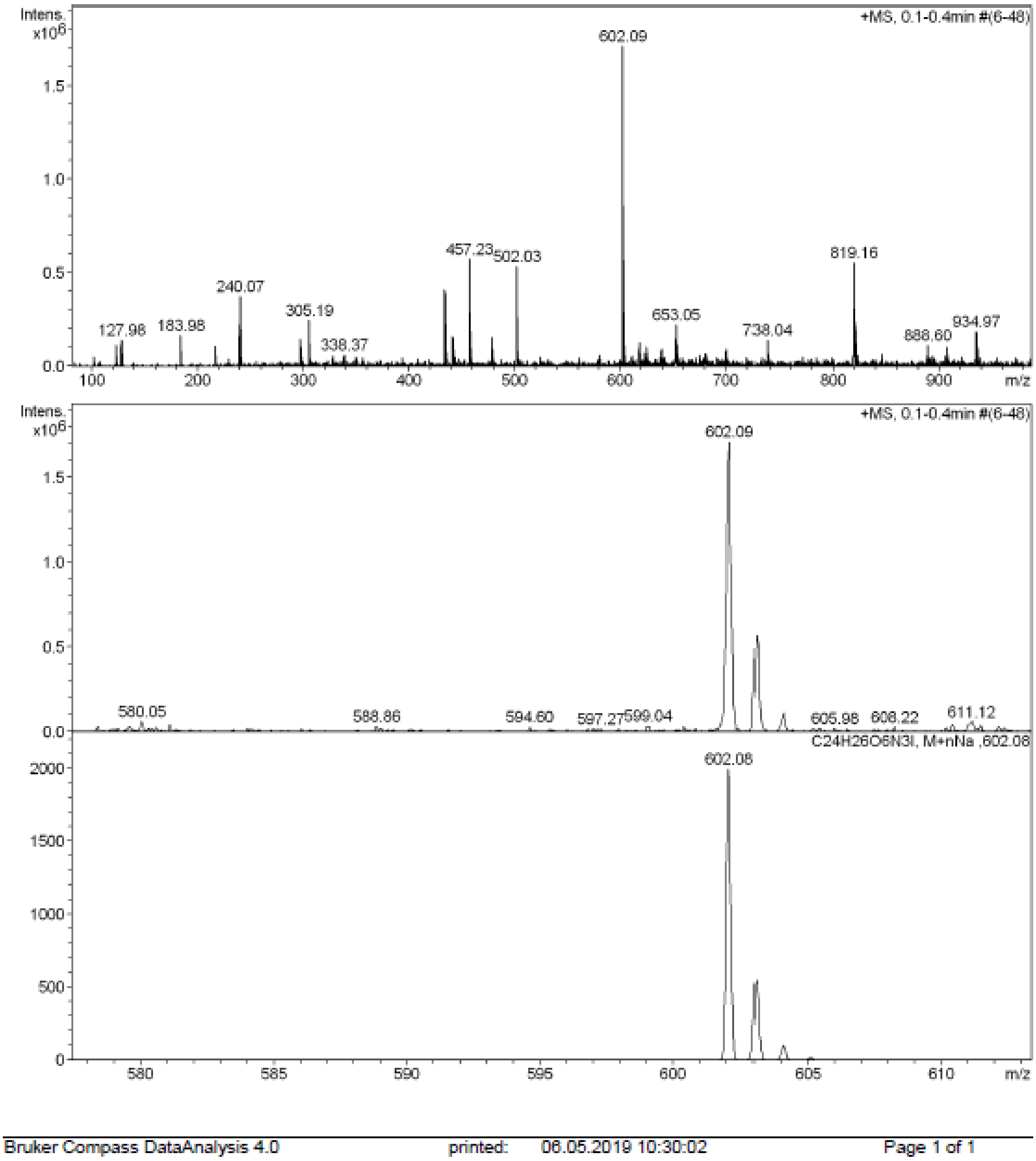

Figure S22. ESI mass spectrum of *tert*-butyl (5-nitro-1-(ethoxymethyl)-1*H*-indole-2-carbonyl)(2-iodobenzyl)carbamate (**L**).

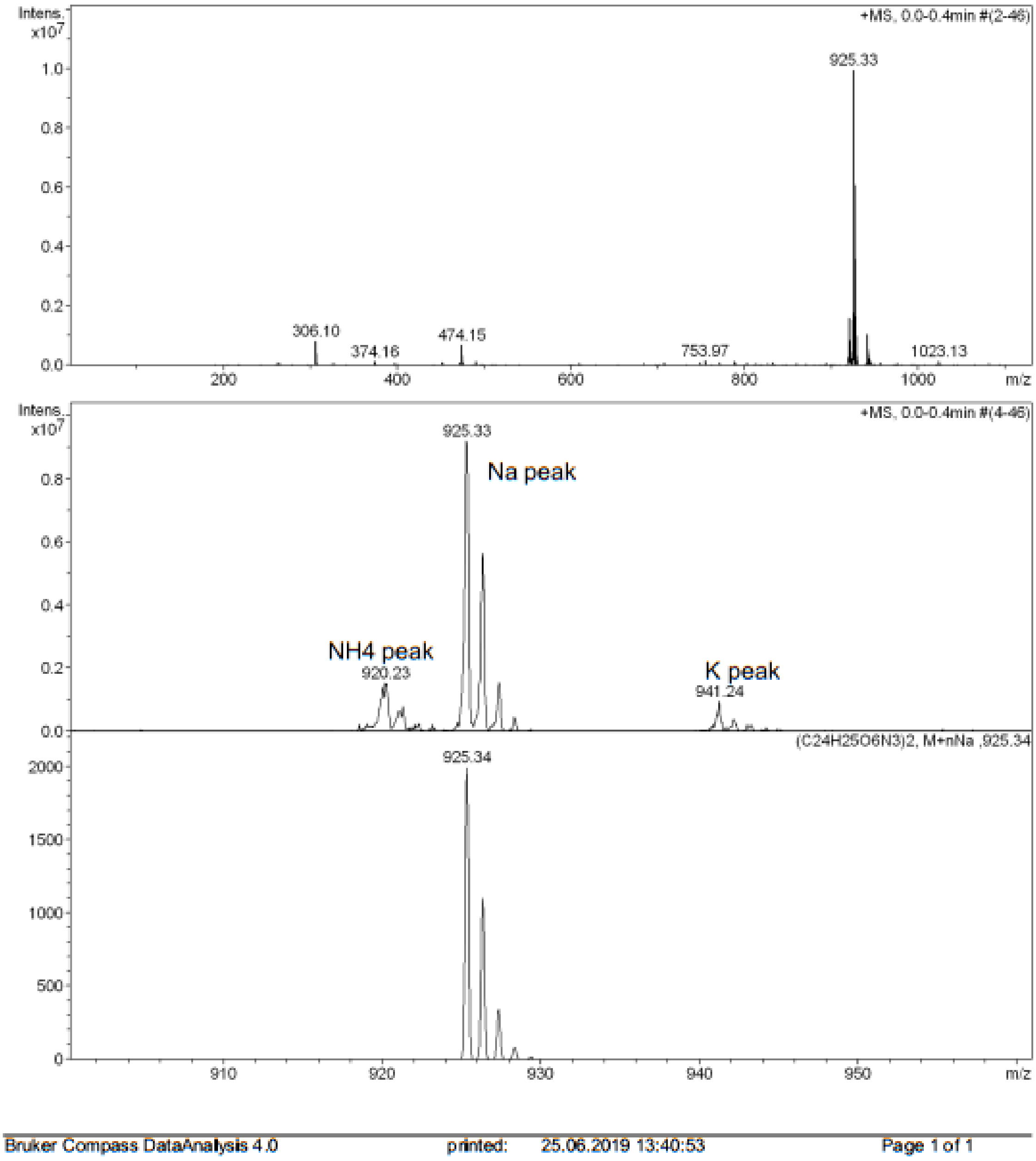

Figure S23. ESI mass spectrum of *tert*-butyl 11-nitro-8-(ethoxymethyl)-dihydroindolo[2,3-*d*]benzazepin-7-one (**M**).

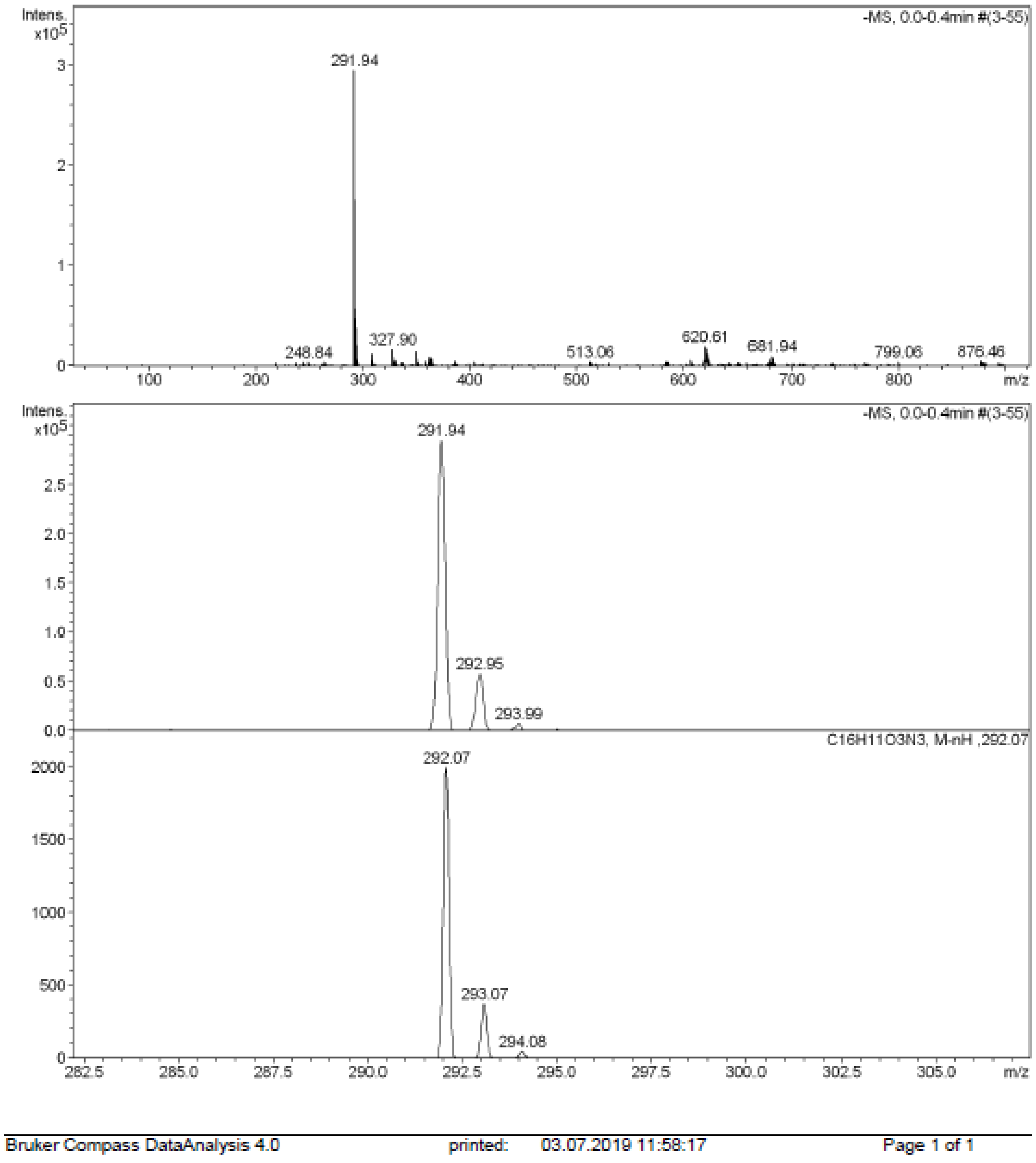

Figure S24. ESI mass spectrum of 11-nitro-5,8-dihydroindolo[2,3-*d*]benzazepin-7(6*H*)-one (**N**).

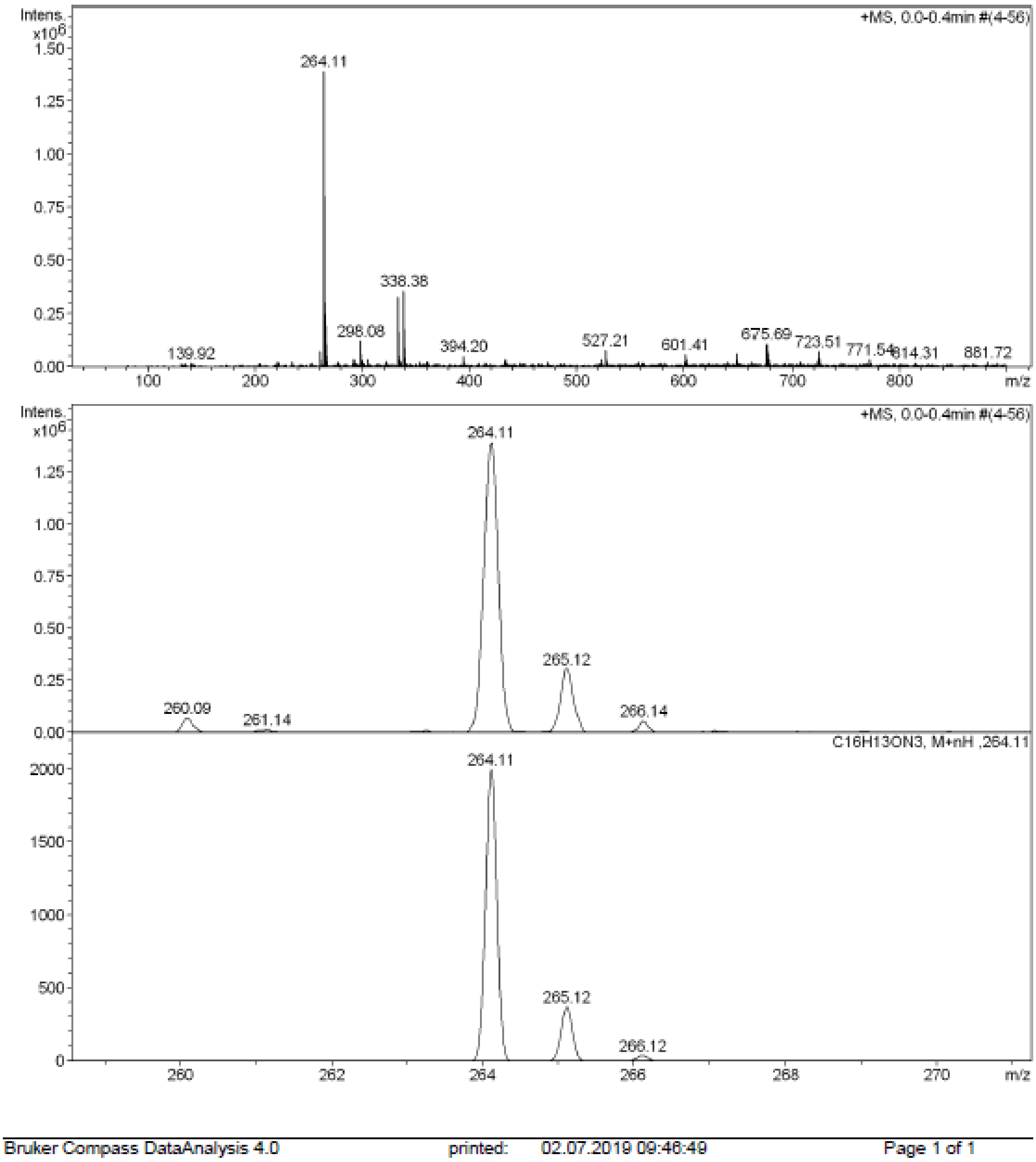

Figure S25. ESI mass spectrum of 11-amino-5,8-dihydroindolo[2,3-*d*]benzazepin-7(6*H*)-one (**O**).

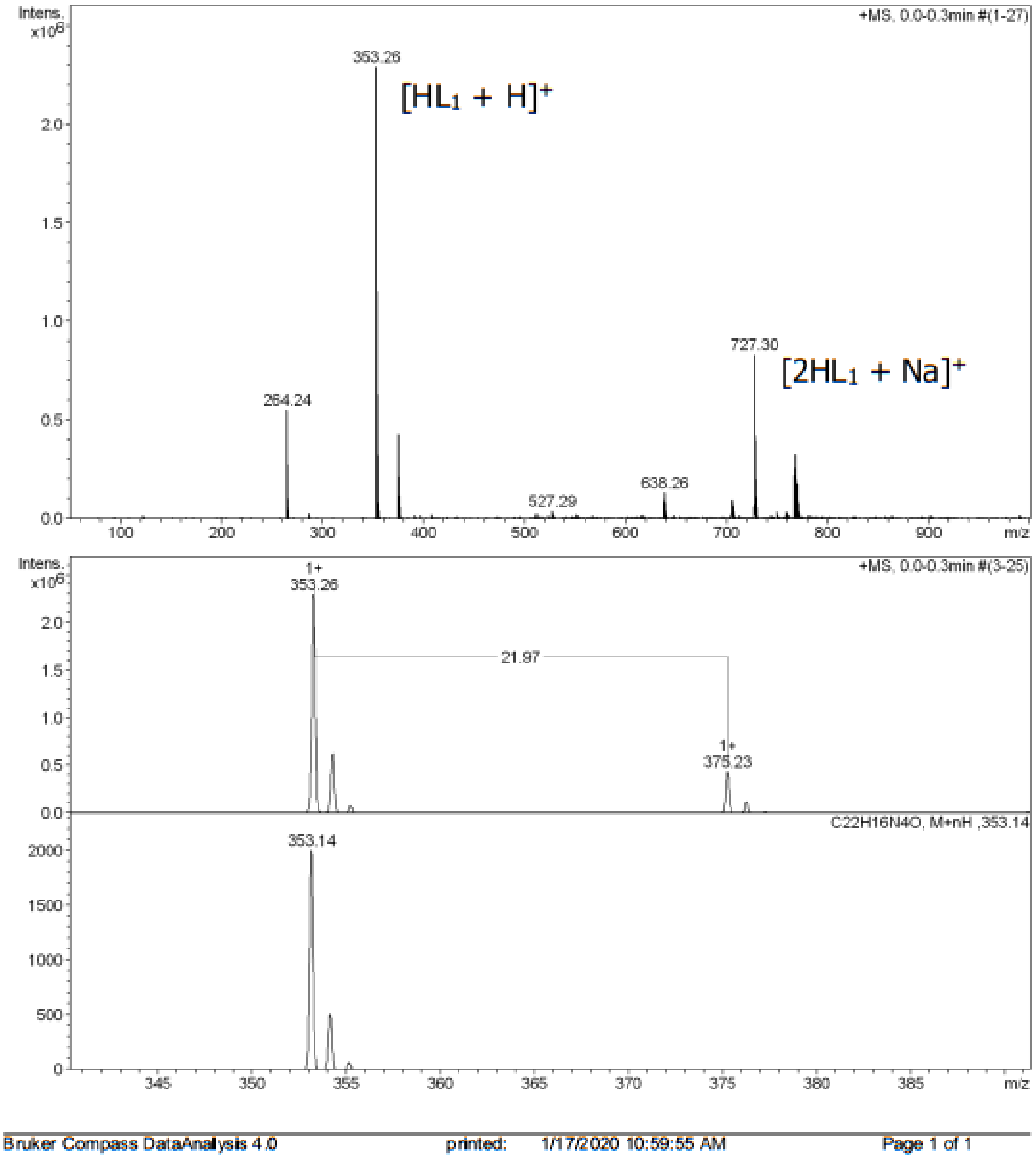

Figure S26. ESI mass spectrum of **HL^1**.

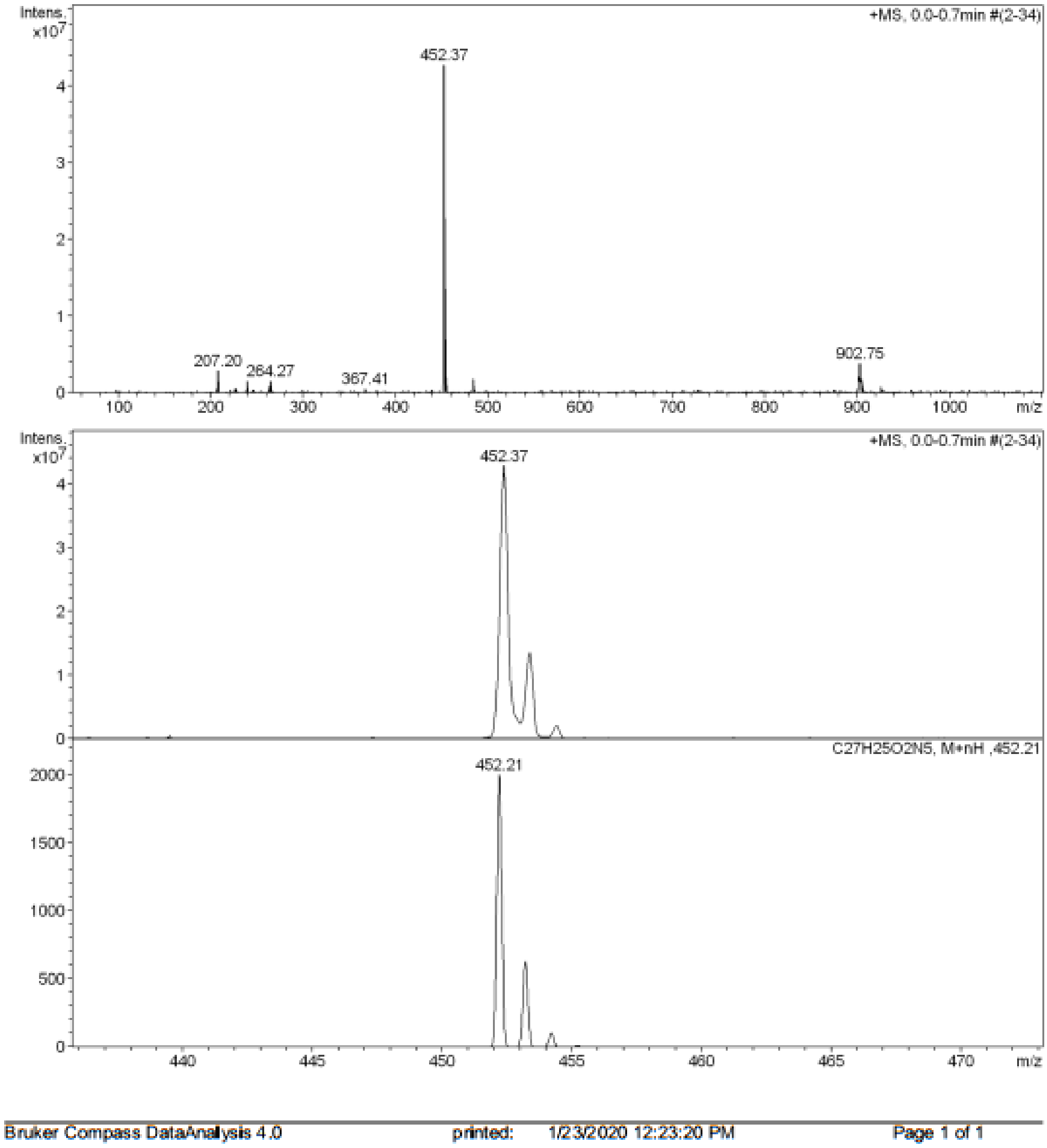

Figure S27. ESI mass spectrum of $\mathbf{HL^2}$.

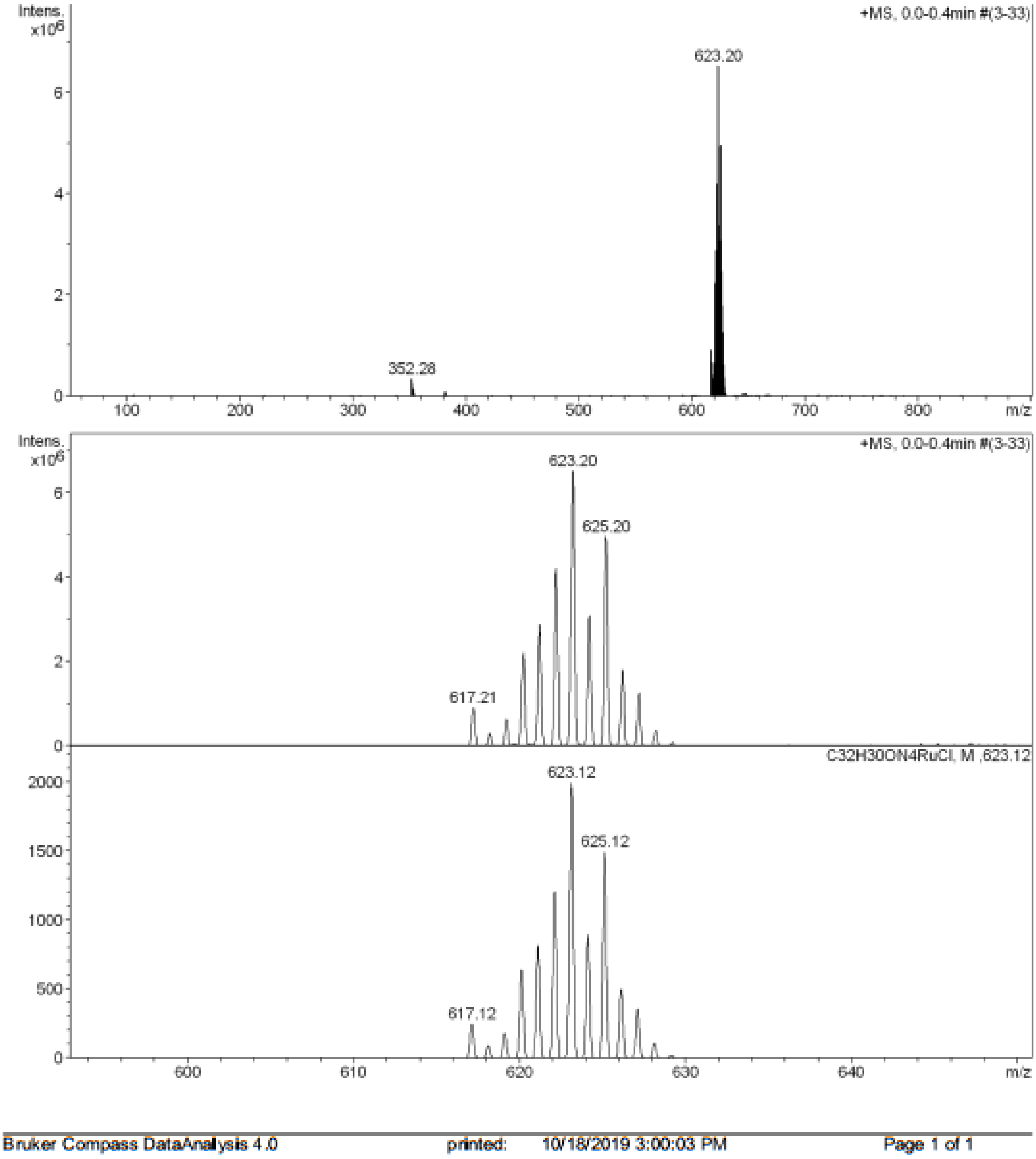

Figure S28. ESI mass spectrum of **1**.

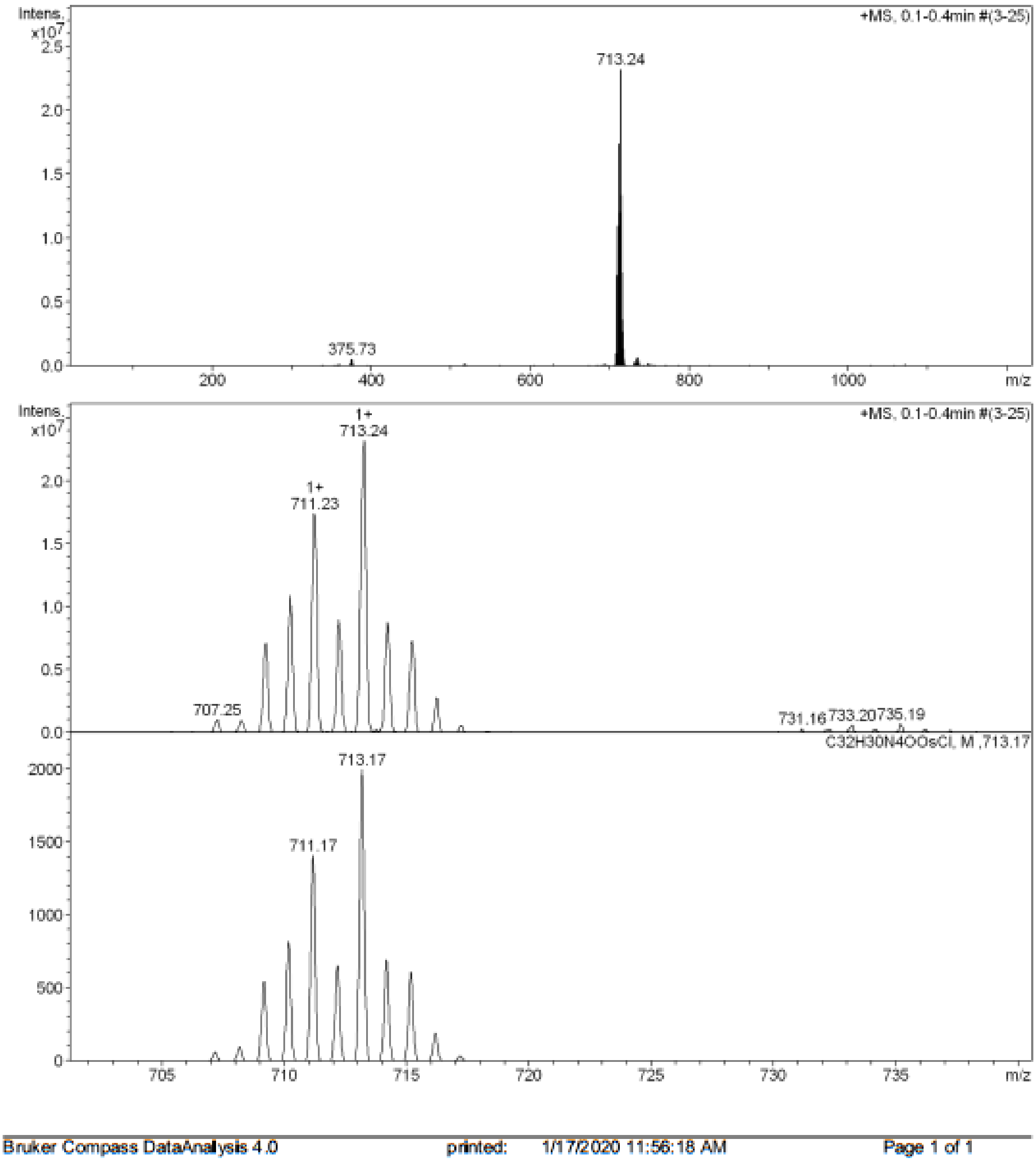

Figure S29. ESI mass spectrum of **2**.

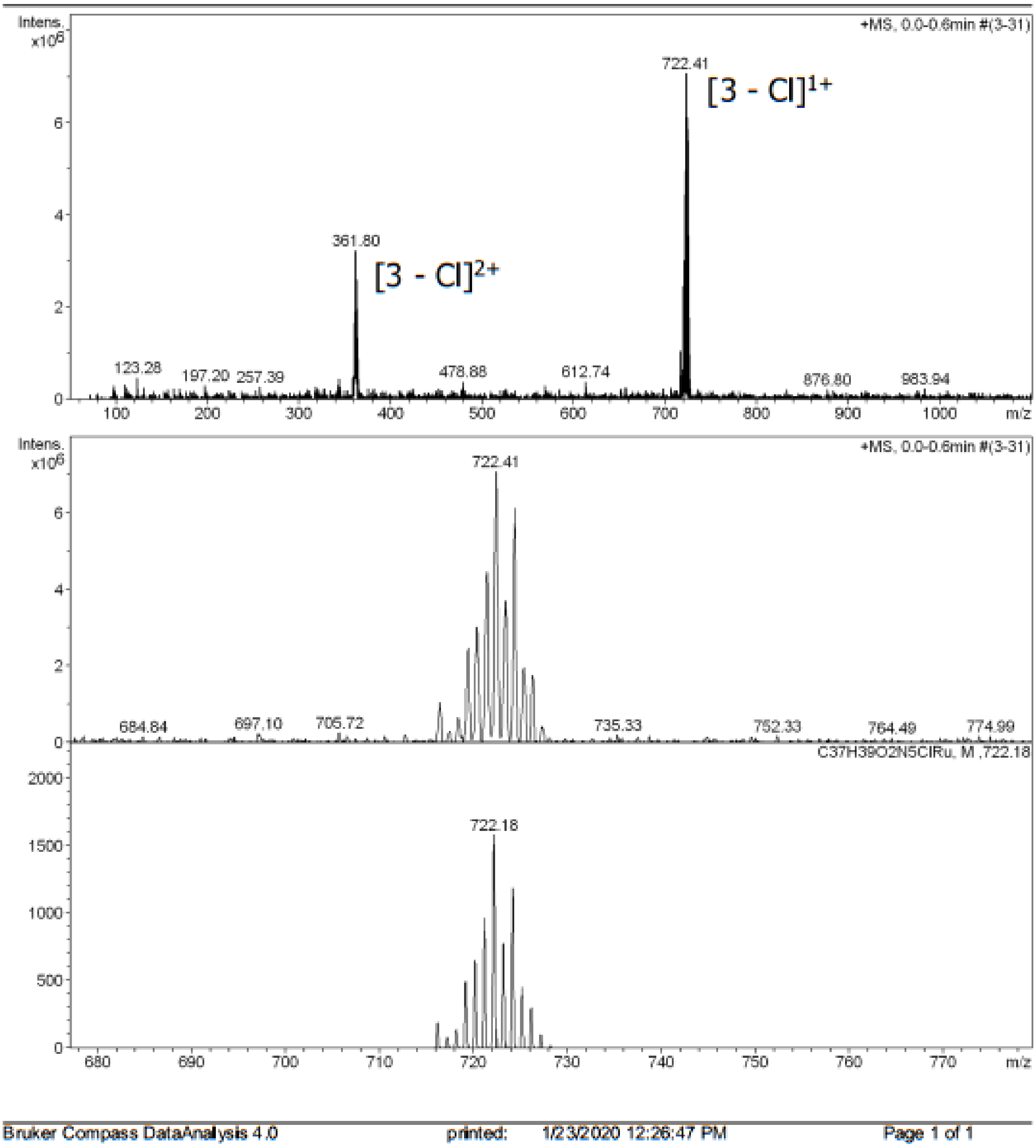

Figure S30. ESI mass spectrum of **3**.

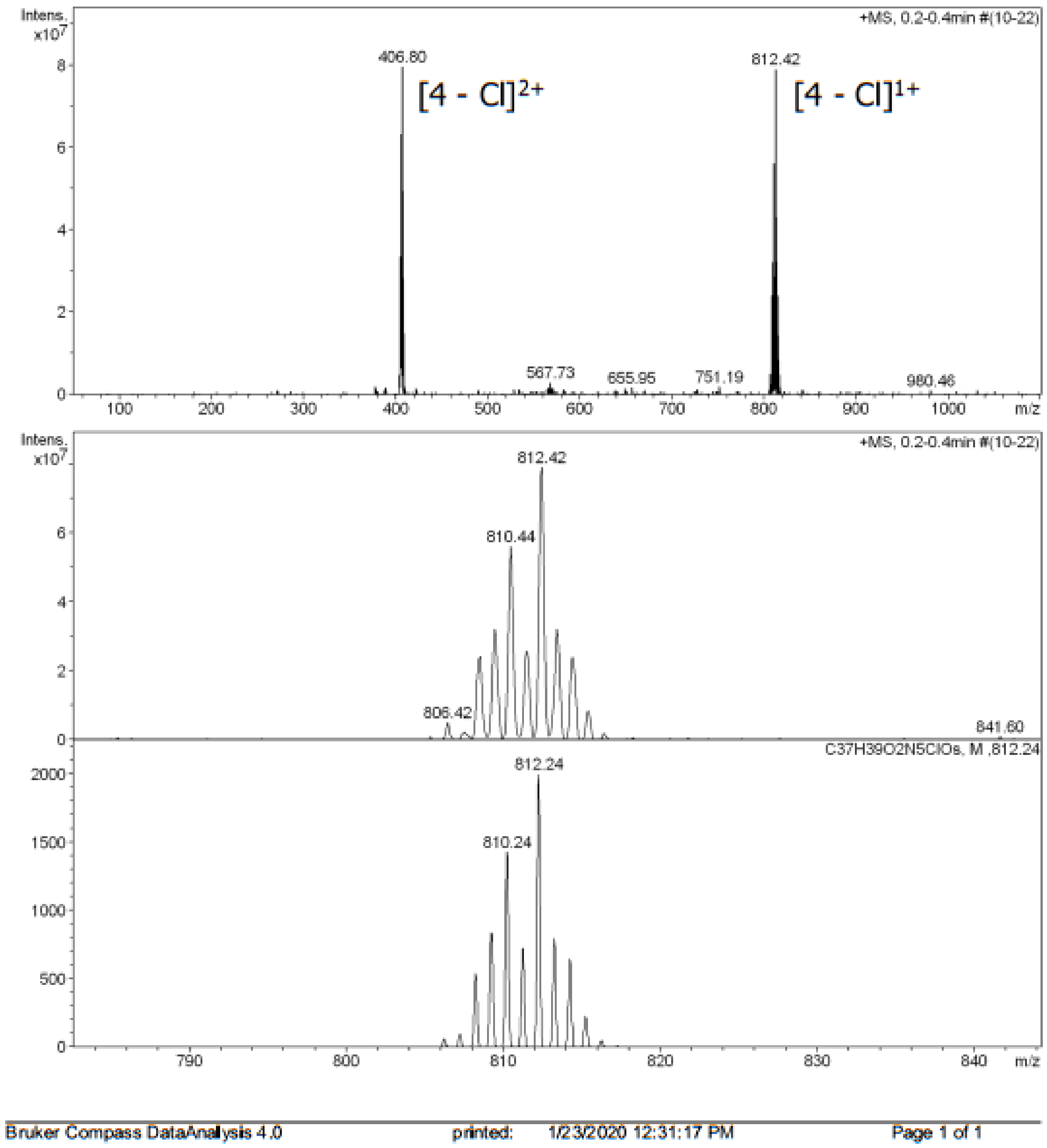

Figure S31. ESI mass spectrum of **4**.

Additional X-ray crystallographic data

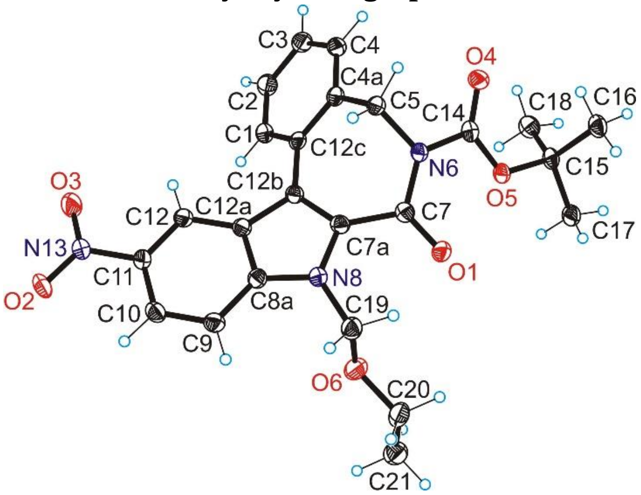

Figure S32. ORTEP view of **M**.

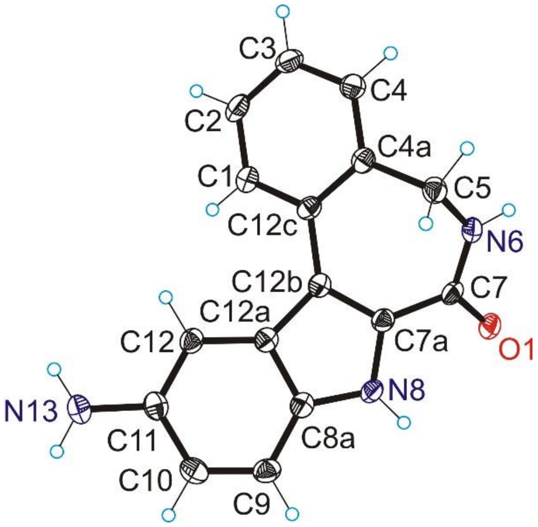

Figure S33. ORTEP view of **P**.

Time-dependent ^{1}H NMR spectra and UV–vis fluorescence data

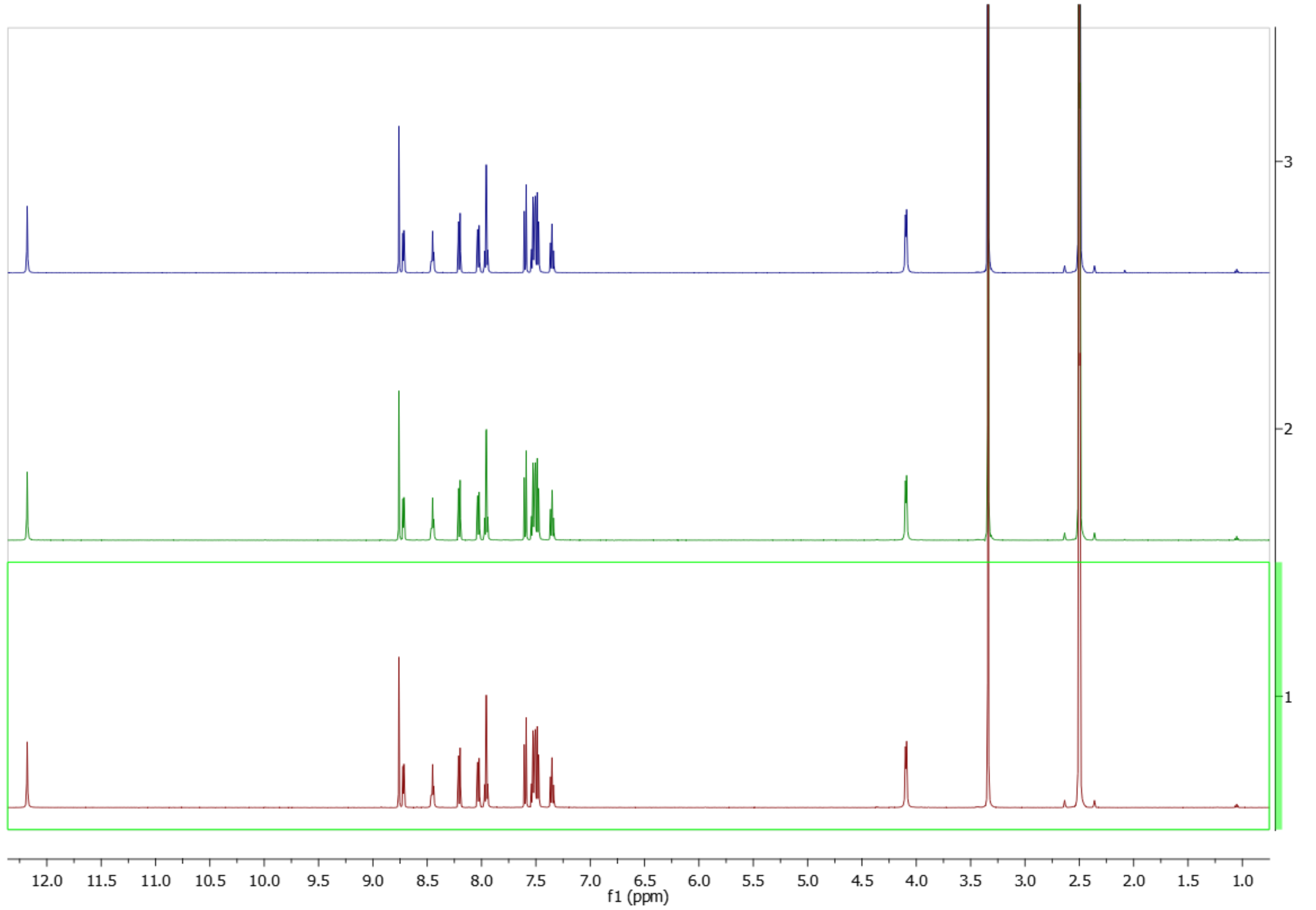

Figure S34. ^{1}H NMR spectra of **HL^1** in DMSO-d_6 measured over time; red: t = 0; green: t = 24 h; blue: t = 96 h.

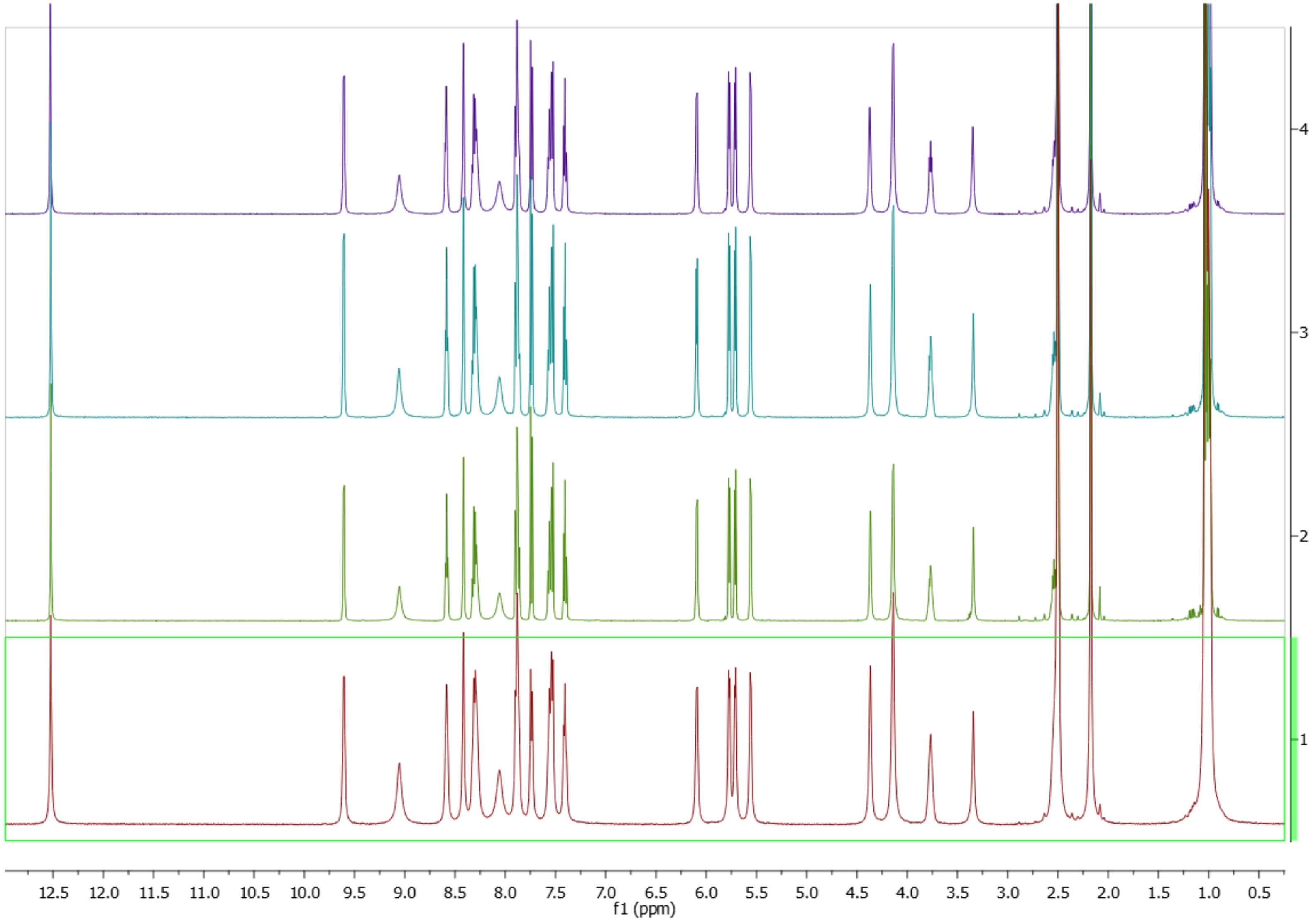

Figure S35. ^{1}H NMR spectra of **1** in DMSO-d_6 measured over time; red: t = 0; green: t = 24 h; turquoise: t =48 h; purple: t = 120 h.

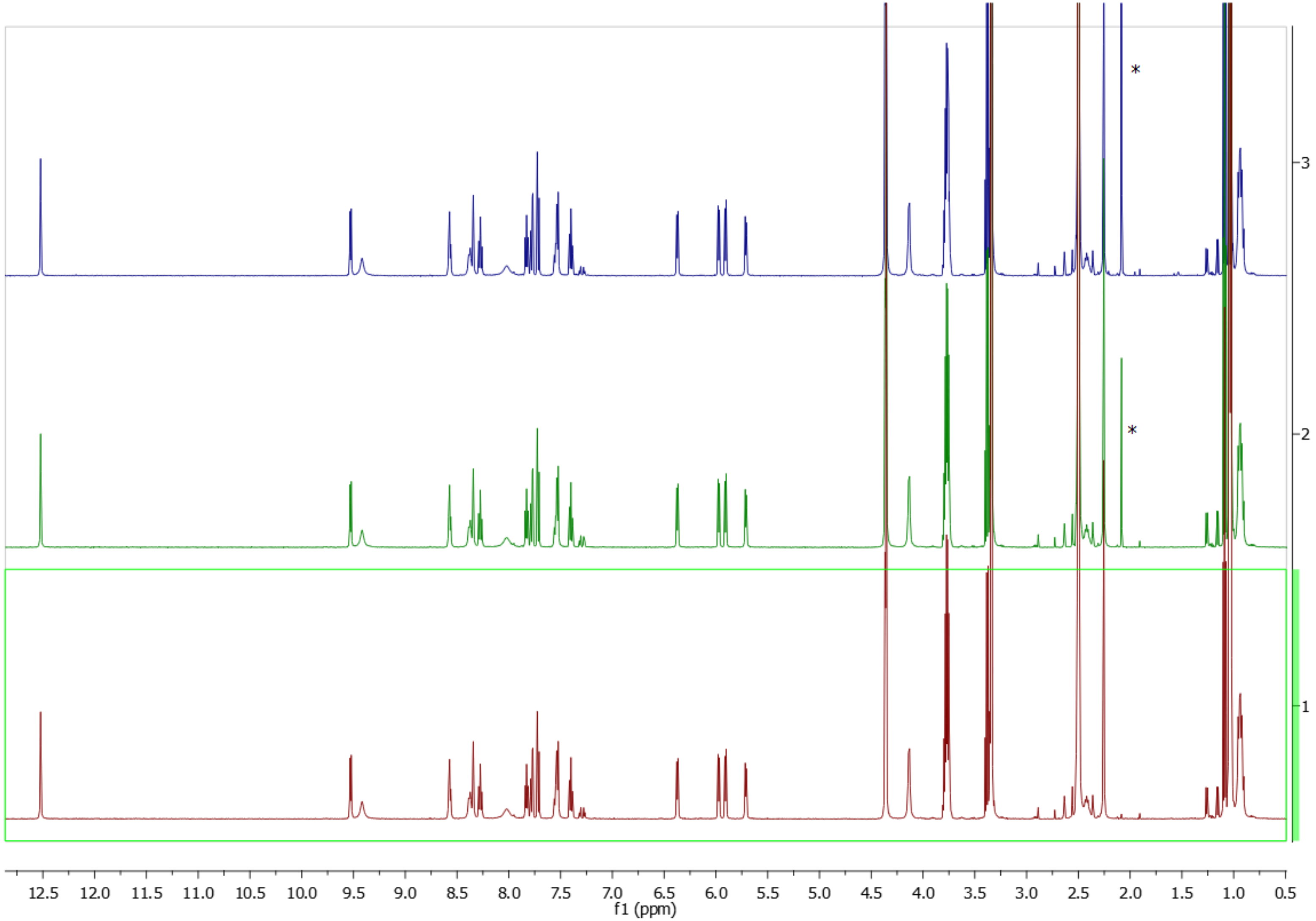

Figure S 36. ^{1}H NMR spectra of **2** in DMSO-d_6 measured over time: red: t = 0; green: t = 24 h; blue: t = 96 h. The peak marked with an asterisk at around 2 ppm is likely due to acetone as impurity.

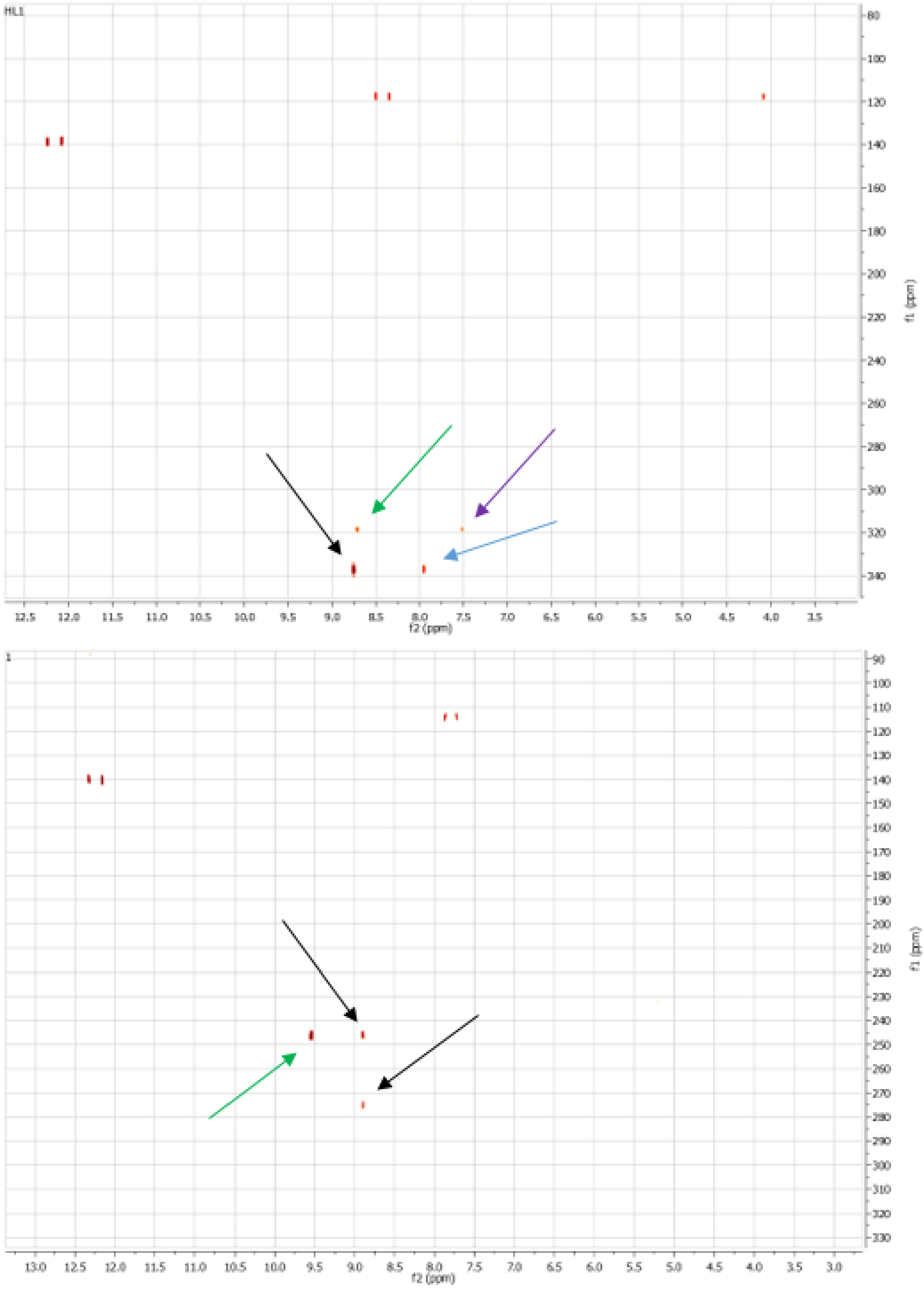

Figure S37. ^{1}H-^{15}N HMBC spectrum of **HL^{1}** (top) and **1** (bottom)in DMSO-d_6. Cross-peaks indicated by blue arrow belongs to proton H12, while those pointed out by black, green and purple arrows to protons H14, H17 and H18, respectively.

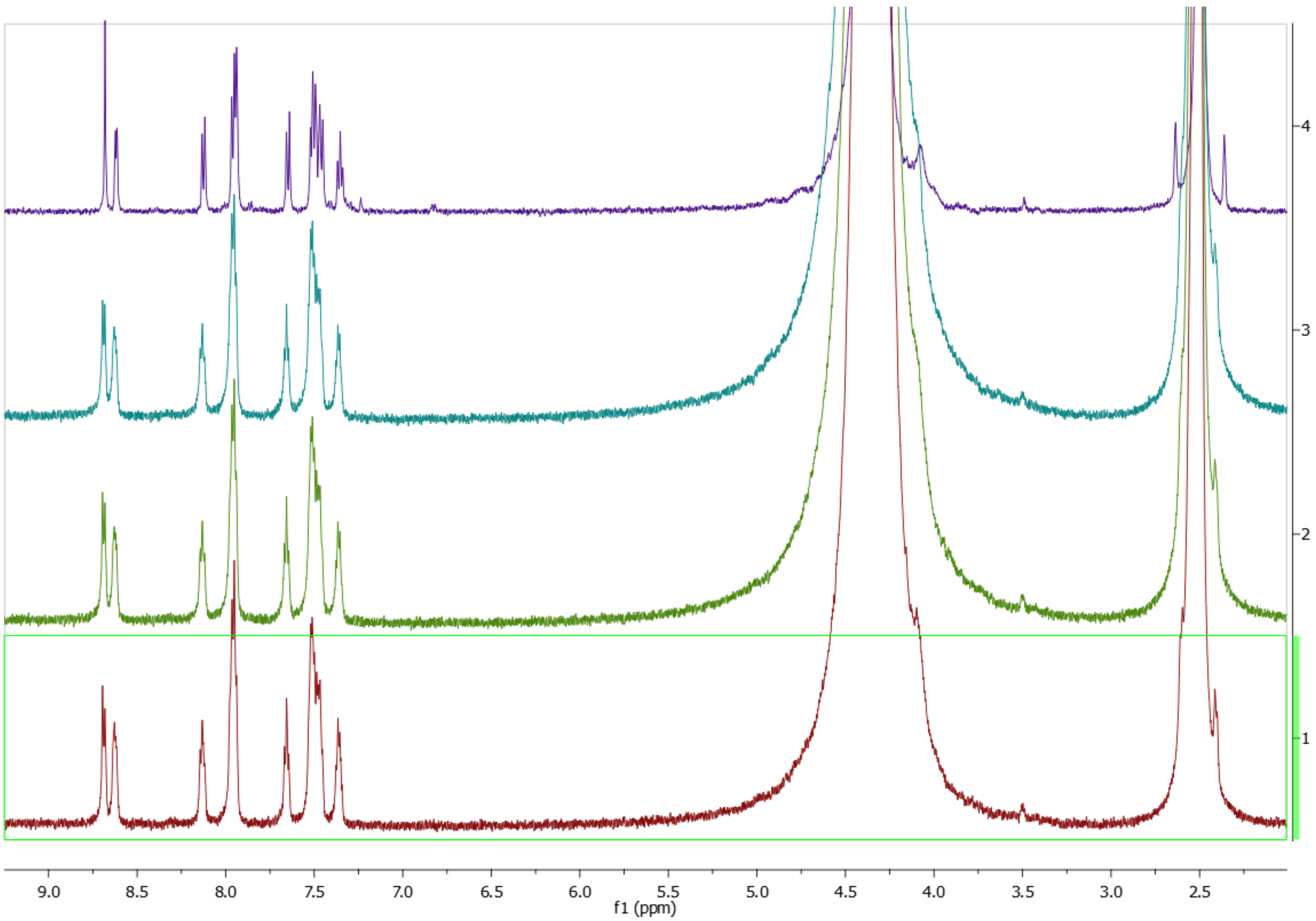

Figure S38. ^{1}H NMR spectra of **HL¹** in DMSO-d_6/D_2O 1:1 measured vs time. From bottom to top: red: t = 0 min; green: t = 30 min; blue: t = 60 min; purple: t = 24 h.

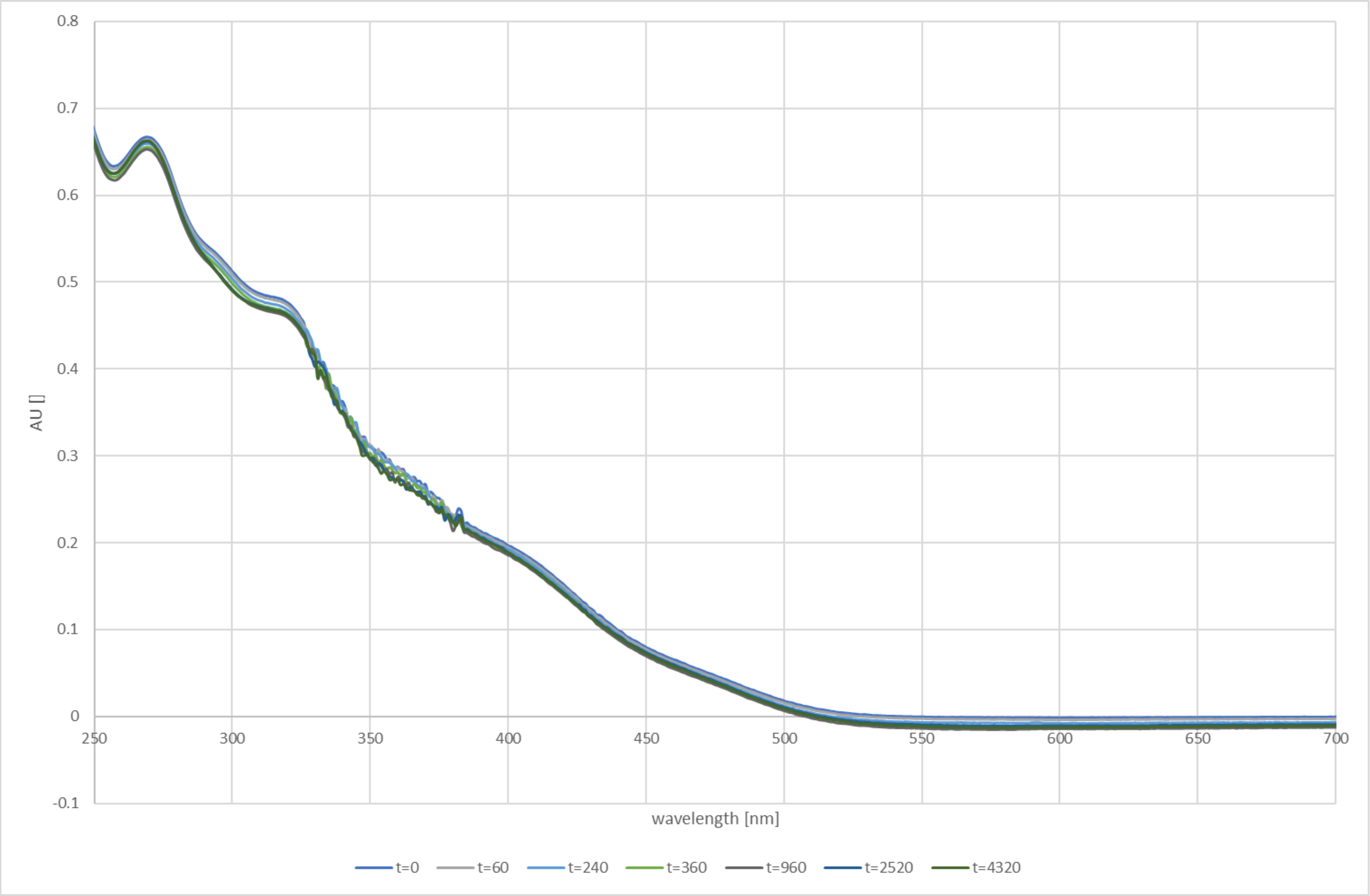

Figure S39. UV–vis stability measurements of **1** over 72 h in 1% DMSO/H_2O (c = 30 µM); time in min.

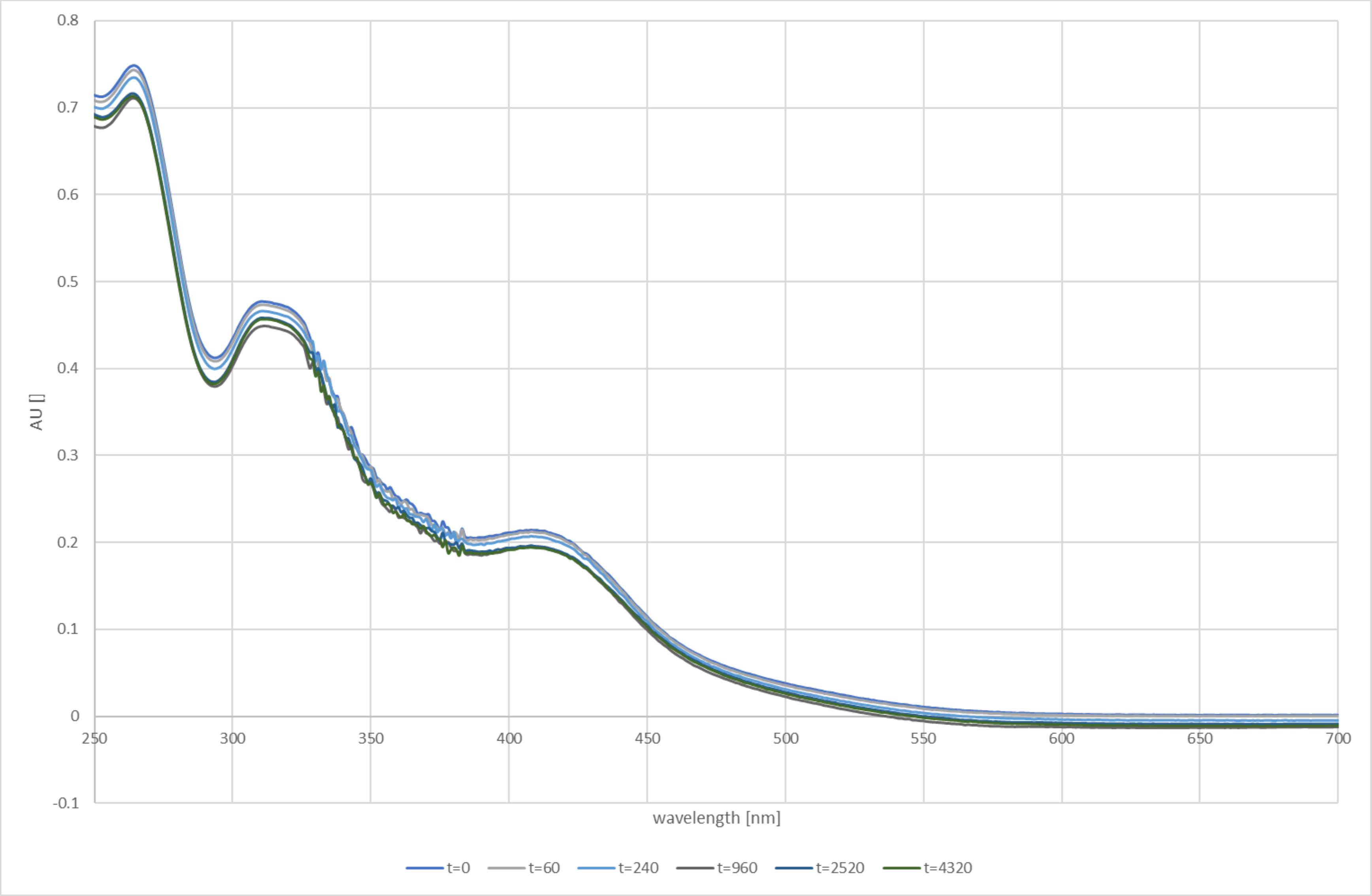

Figure S40. UV–vis stability measurements of **2** over 72 h in 1% DMSO/H_2O (c = 30 μM); time in min.

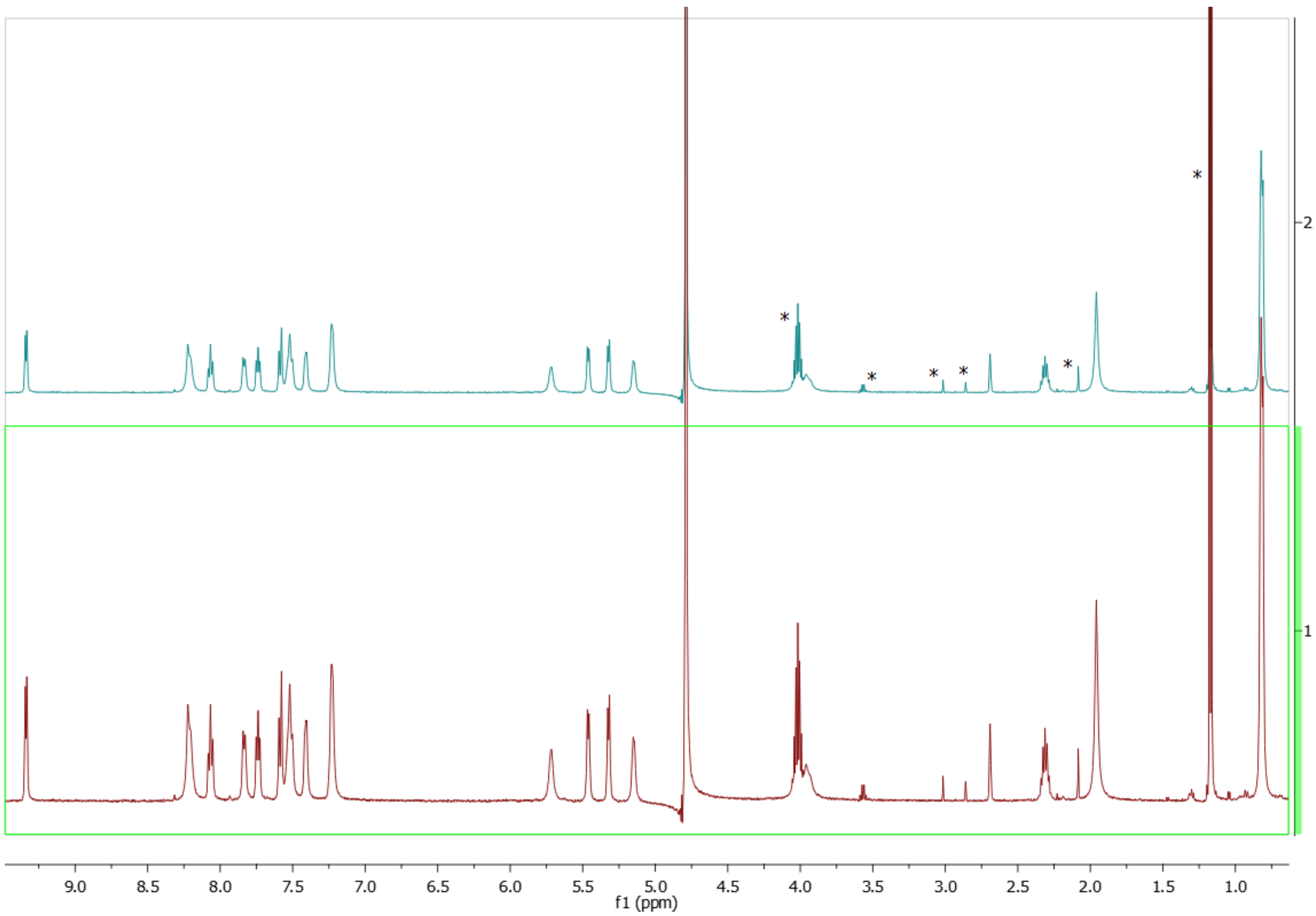

Figure S41. ^{1}H NMR spectra of of **1** in 5% DMSO-d_6/D_2O measured vs time. From bottom to top: red: t = 0 min; blue: t = 12 h. Residual solvents are highlighted with *, namely DMF, iPrOH and Et_2O.

High Performance Liquid Chromatography-MS report of 1

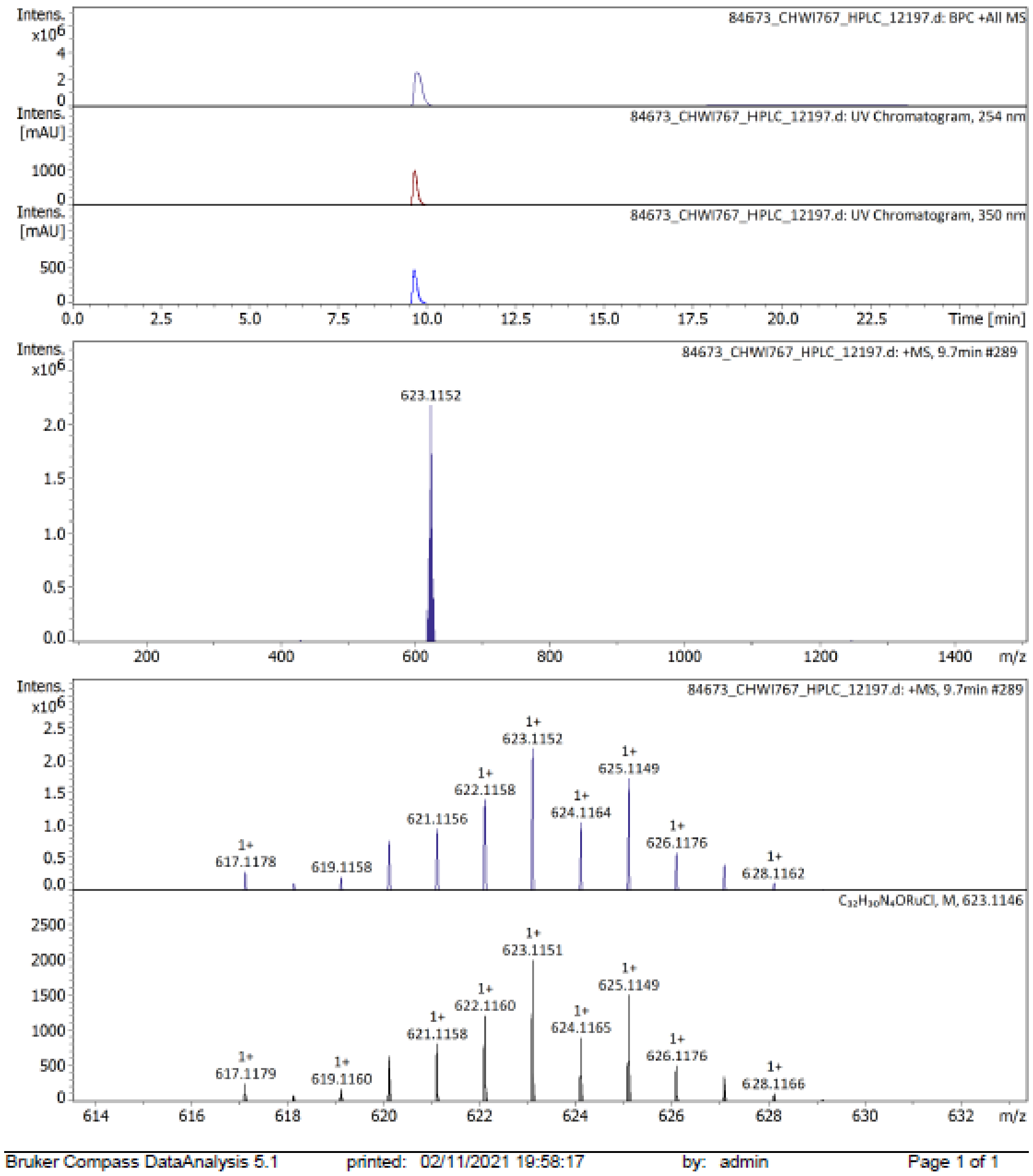

Figure S42. Purity control of complex **1** via HPLC and HR ESI MS for *in vivo* treatment of mice.

Concentration-effect curves

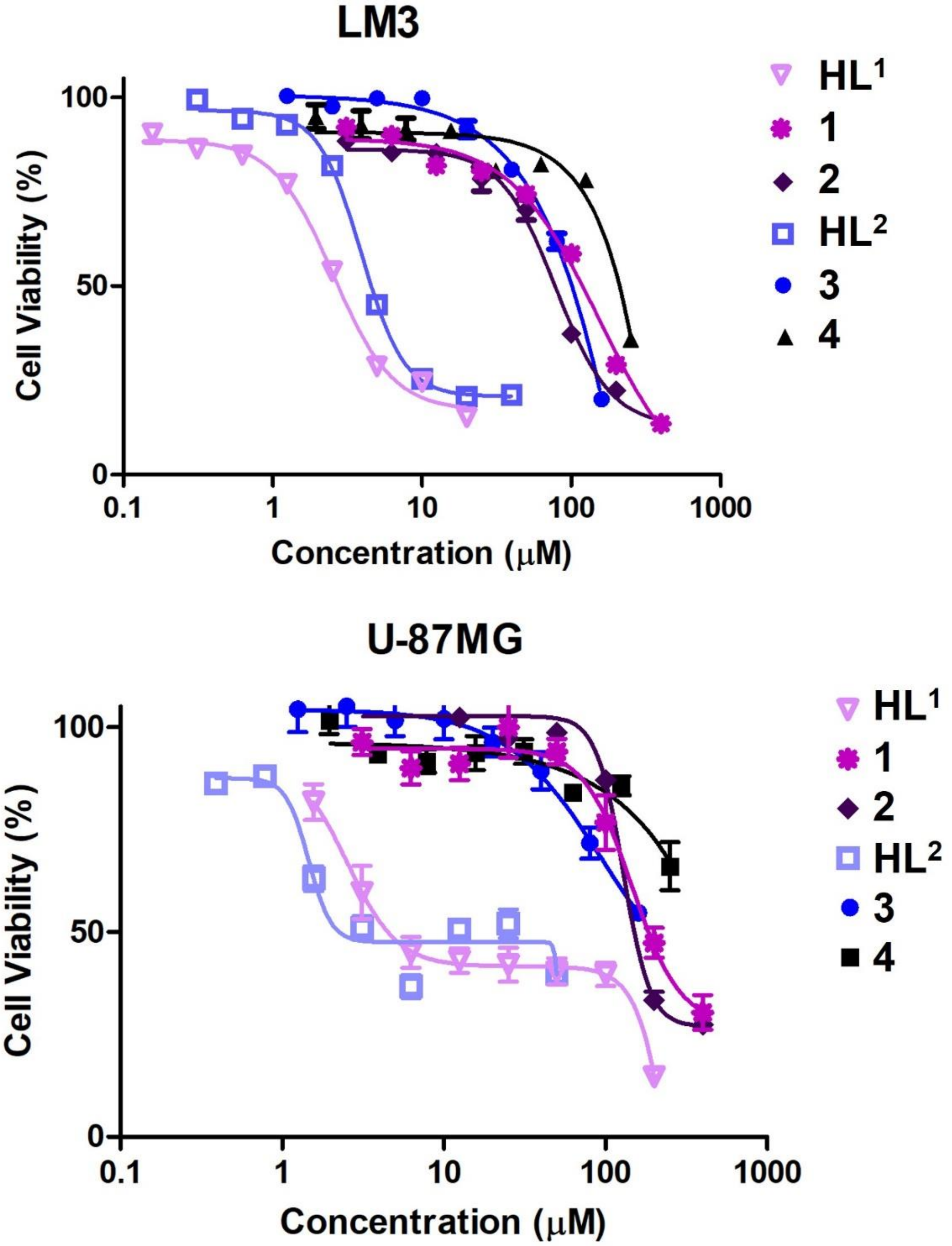

Figure S43. Concentration–effect curves of **HL¹** and **HL²** and corresponding Ru^{II} complexes **1** and **3** and Os^{II} complexes **2** and **4** in the human hepatocellular carcinoma cell line LM3 and human glioma cell line U-87 MG.

Table S1. The molecular descriptors based on the Lipinski´s rules[b] as calculated by SwissADME, as well as experimental solubility and pH-values.

Compound	MW	HD	HA	Log*P*[a]	Log *S* (*S*, mol/L) (ESOL)[c]	Log *S* (*S*, mol/L) (exptl)[d]	pH-values
**HL[1]**	352.39	2	3	3.31	–4.62 (2.38×10^{-5})	–2.94 (1.14×10^{-3})	7.8[e]
**HL[2]**	451.52	2	5	3.15	–4.65 (2.25×10^{-5})	–3.05 (8.86×10^{-4})	7.4[f]
**1**	654.55	2	1	3.74	–7.97 (1.07×10^{-8})	≥ –2.34 (≥ 4.56×10^{-3})	6.6[g]
**2**	743.71	2	1	3.85	–8.53 (2.98×10^{-9})	≥ –2.40 (≥ 4.01×10^{-3})	6.4[g]
**3**	753.68	2	3	3.31	–8.06 (8.77×10^{-9})	≥ –2.40 (≥ 3.96×10^{-3})	5.9[g]
**4**	842.84	2	3	3.42	–8.61 (2.46×10^{-9})	≥ –2.45 (≥ 3.54×10^{-3})	5.5[g]

[a]Log*P* = partition coefficient between octanol and water; [b]Lipinski: MW ≤ 500 g/mol; Log*P* ≤ 5; H-bond donors ≤ 5; H-bond acceptors ≤ 10; [c]Solubility class (Log *S* scale): insoluble < –10 < poorly < –6 < moderately < –4 < soluble < –2 very < 0 < highly; [d]experimental solubility parameters in 1% DMSO/H_2O; [e]uncorrected pH measured at the concentration of 1 mg/mL in 50% DMSO/H_2O at 20 °C; [f]uncorrected pH measured at the concentration of 1.2 mg/mL in 50% DMSO/H_2O at 20 ° C; [g]pH measured at 1.6 mg/mL in 1% DMSO/H_2O at 20 °C.

Analysis reports on mice treated with complex 1

Table S2. Blood chemistry report of mice treated with **1** (10 mg/kg) for MTD tests

Name	1LL10	2N10	Reference Range
Sex<br>Age	Female<br>37 days	Female<br>37 days	
BUN (mg/dL)	32	28	5.0-28
CREA (mg/dL)	0.23	0.28	0.2-0.5
ALP (U/L)	86	110	105-370
ALT (U/L)	34	23	27-195
AST (U/L)	110	99	54-77
GGT (U/L)	0	0	-
TBIL (U/L)	0.2	0.1	0.2-0.6
DBIL (U/L)	0	0	-
IBIL	0.2	0.1	-
TP (U/L)	5.3	5.4	4.8-7.2
ALB (U/L)	3.2	3.1	2.4-4.3
GLOB (U/L)	2.1	2.3	1.7-2.2
P (mg/dL)	7.5	7.5	7.3-14.5
Ca (mg/dL)	9.6	10.1	9.5-12.5
GLU (mg(dL)	155	221	172-372
CHOL (mg/dl)	86	98	55-169
TRIG (mg/dL)	194	173	67-289
CK (U/L)	240	266	428-1609
TCO2 (mEq/L)	33	22	-
Na (mEq/L)	153	152	145-181
K (mEq/L)	7.2	7	7.3-11.1
CL (mEq/L)	108	110	111-134
Anion Gap	19	27	-

Table S3. Blood chemistry report of mice bearing LX22 tumors treated with **1** (7.5 mg/kg mg/kg) every other day.

Name	1L	1R	1LR	2LL	Reference Range
Sex<br>Age	Female<br>75 days	Female<br>75 days	Female<br>75 days	Female<br>75 days	
BUN (mg/dL)	28	31	23	26	5.0-28
CREA (mg/dL)	0.26	0.25	0.2	0.25	0.2-0.5
ALP (U/L)	55	59	43	33	105-370
ALT (U/L)	26	35	44	28	27-195
AST (U/L)	187	335	115	100	54-77
GGT (U/L)	0	0	0	0	-
TBIL (U/L)	0.2	0.2	0.2	0.2	0.2-0.6
DBIL (U/L)	0	0	0	0	-
IBIL	0.2	0.2	0.2	0.2	-
TP (U/L)	4.8	4.4	4.6	5.3	4.8-7.2
ALB (U/L)	2.8	2.6	2.6	2.7	2.4-4.3
GLOB (U/L)	2	1.8	2	2.6	1.7-2.2
P (mg/dL)	7.5	8	8.6	7.4	7.3-14.5
Ca (mg/dL)	10.2	9.6	9.8	10.2	9.5-12.5
GLU (mg(dL)	186	146	171	173	172-372
CHOL (mg/dl)	128	105	138	131	55-169
TRIG (mg/dL)	301	190	208	136	67-289
CK (U/L)	335	1232	80	157	428-1609
TCO2 (mEq/L)	31	26	27	28	-
Na (mEq/L)	160	157	154	156	145-181
K (mEq/L)	7.6	7.5	7.9	7.5	7.3-11.1
CL (mEq/L)	112	114	110	108	111-134
Anion Gap	25	25	25	28	-
